# Panchromatic Absorbers Tethered for Bioconjugation or Surface Attachment

**DOI:** 10.3390/molecules27196501

**Published:** 2022-10-01

**Authors:** Rui Liu, Jie Rong, Zhiyuan Wu, Masahiko Taniguchi, David F. Bocian, Dewey Holten, Jonathan S. Lindsey

**Affiliations:** 1 Department of Chemistry, North Carolina State University, Raleigh, NC 27695-8204, USA; 2 Department of Chemistry, University of California, Riverside, CA 92521-0403, USA; 3 Department of Chemistry, Washington University, St. Louis, MO 63130-4889, USA

**Keywords:** array, artificial photosynthesis, building block, copper-free Sonogashira coupling, ethyne, perylene, porphyrin, solar

## Abstract

The syntheses of two triads are reported. Each triad is composed of two perylene-monoimides linked to a porphyrin via an ethyne unit, which bridges the perylene 9-position and a porphyrin 5- or 15-position. Each triad also contains a single tether composed of an alkynoic acid or an isophthalate unit. Each triad provides panchromatic absorption (350–700 nm) with fluorescence emission in the near-infrared region (733 or 743 nm; fluorescence quantum yield ~0.2). The syntheses rely on the preparation of *trans*-AB-porphyrins bearing one site for tether attachment (A), an aryl group (B), and two open meso-positions. The AB-porphyrins were prepared by the condensation of a 1,9-diformyldipyrromethane and a dipyrromethane. The installation of the two perylene-monoimide groups was achieved upon the 5,15-dibromination of the porphyrin and the subsequent copper-free Sonogashira coupling, which was accomplished before or after the attachment of the tether. The syntheses provide relatively straightforward access to a panchromatic absorber for use in bioconjugation or surface-attachment processes.

## 1. Introduction

Chromophores that absorb across the visible spectrum (400–700 nm) are of essential importance for diverse studies in the photosciences. The chlorophylls of plant photosynthesis exhibit strong absorption in the violet and red regions, with relatively weak absorption across the rest of the visible region [1]. Natural photosynthetic systems use carotenoids and/or other accessory chromophores in complementary fashion to fill the blue–orange region of the solar spectrum [2]. In this approach, the distinct absorption of a given accessory pigment is followed by excited-state energy transfer among a set of pigments, thereby increasing the wavelength expanse of absorption beyond that of chlorophyll alone. Work that began some 45 years ago [3] led to the realization in the early 1990s that the absorption spectrum of porphyrins could be substantially broadened upon the conjugation of ethynyl groups to the macrocycle [4,5,6,7]. Penetrating studies thereafter by the groups of Anderson [8] and Therien [9,10,11,12,13,14,15,16,17,18,19,20,21] chiefly focused on joining porphyrins via butadiyne and ethyne linkers, respectively, eliciting fascinating spectroscopic features of the resulting arrays. The joining of porphyrins and other chromophores via ethynyl linkers quickly became a prominent molecular motif [22,23,24,25], a topic that has been reviewed [26,27].

**Figure 1:**
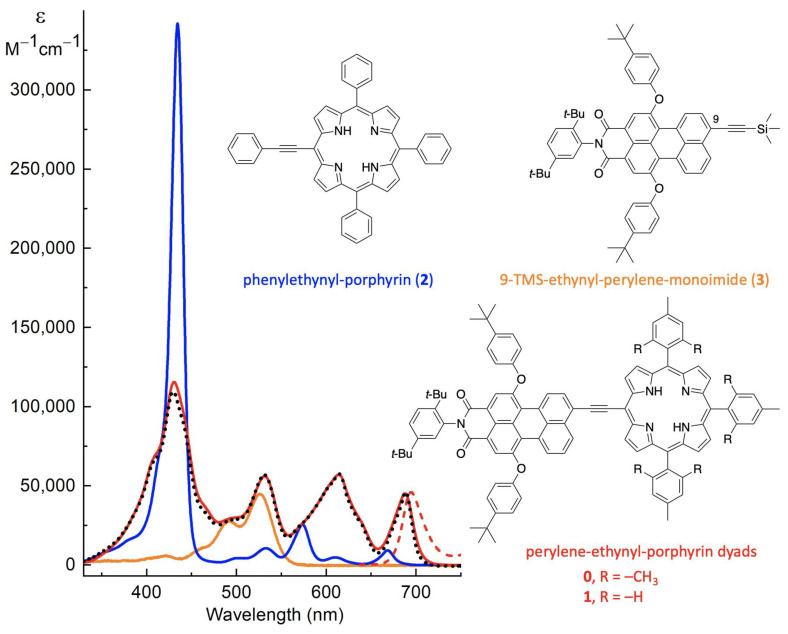
Absorption spectra of a perylene-ethynyl-porphyrin dyad (**1**, solid red line), to be compared with phenylethynyl-porphyrin (**2**, solid blue line) and perylene-ethyne (**3**, solid orange line) constituents (in toluene at room temperature). The fluorescence spectrum of **1** also is shown (dashed red line; λ_em_ 695 nm) [42]. The absorption spectrum of the perylene-ethynyl-porphyrin dyad **0** (dotted black trace [41]) is essentially identical with that of analogous dyad **1** (solid red line).

In a quite separate research thread, we had prepared and characterized a large number of tetrapyrrole-perylene constructs for studies of excited-state energy transfer as part of a program in artificial photosynthesis [28,29,30,31,32,33,34,35,36,37,38,39,40]. In such constructs, we had employed long linkers such as phenyl-ethynyl-phenyl to separate the tetrapyrrole and perylene or ethynylphenyl joining the pigments in the manner perylene-ethynylphenyl-tetrapyrrole. As an intended “last molecular design” in this area [41], we omitted any phenyl groups and prepared a perylene-ethynyl-porphyrin dyad (**0**). The dyad exhibited such broad and unusual absorption across the visible region that the characteristic Soret band, a hallmark of porphyrins, was almost unrecognizable (Figure 1) [41].

For exacting comparison, we then prepared an analogous perylene-ethynyl-porphyrin dyad (**1**) [42], which differed from the initial dyad (**0**) in the nature of the non-linking substituents (*p*-tolyl versus mesityl groups) on the porphyrin, and also the corresponding ethynyl-porphyrin (**2**) [43,44] and ethynyl-perylene (**3**) [40] benchmark compounds bearing nearly identical substituents. The change from mesityl to *p*-tolyl substituents was inconsequential, as shown in the overlay of the spectra of dyads **0** and **1**. While the absorption spectrum of each dyad resembles that of a strongly potentiated perylene and diminished porphyrin (Figure 1), the fluorescence emission spectrum resembles that of a porphyrin, albeit one shifted bathochromically (~40 nm) and with substantially increased intensity. Indeed, the fluorescence quantum yield (Φ_f_) of dyad **1** is 0.38 instead of 0.14 for the phenylethynyl-substituted porphyrin **2** [42].

**Chart 1:**
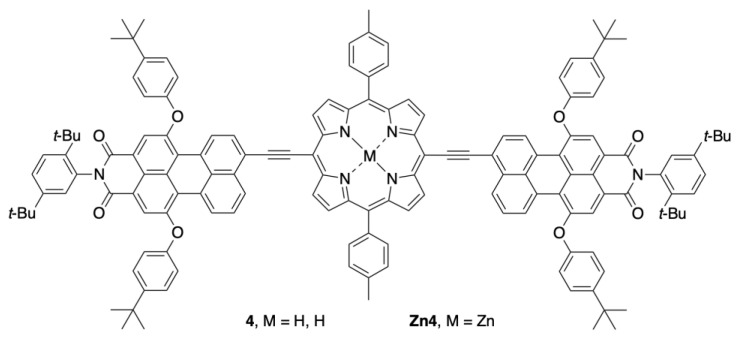
Structure of panchromatic triad (**4**) and the zinc chelate **Zn4**.

**Figure 2:**
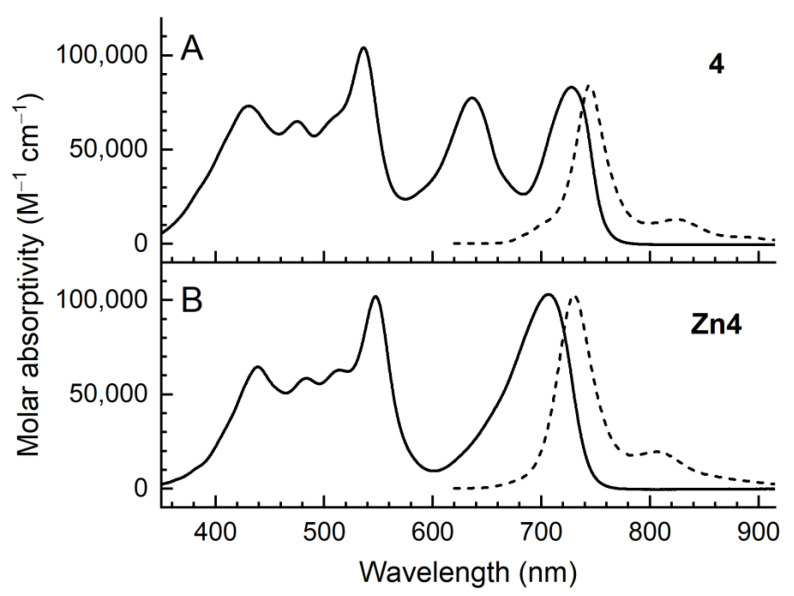
Absorption spectra (solid line) and fluorescence spectra (dashed line) of triads in toluene at room temperature. The molar absorption coefficient values for free base porphyrin triad **4** are as follows: 431 nm (73,200 M^−1^cm^−1^), 537 nm (104,000 M^−1^cm^−1^), 637 nm (77,400 M^−1^cm^−1^), and 728 nm (83,200 M^−1^cm^−1^) [42,45]. The molar absorption coefficient values for zinc porphyrin triad **Zn4** are as follows: 439 nm (64,700 M^−1^cm^−1^), 548 nm (102,000 M^−1^cm^−1^), and 707 nm (103,000 M^−1^cm^−1^) [45]. The Φ_f_ value of **4** is 0.26, whereas that of **Zn4** is 0.30 [45].

This unexpected finding in 2013 [41] did not close the experimental program but instead prompted an additional decade of research [45,46,47,48,49,50] to establish the physical basis for the origin of panchromaticity. An ideal panchromatic absorber was found to be a porphyrin bearing two perylene-monoimides each joined via a single ethyne unit bridging the perylene 9-position and a porphyrin meso-position (**4**, Chart 1). The absorption and fluorescence spectra of **4** are shown in Figure 2, upper panel. Conversion of the free base porphyrin to the zinc chelate (**Zn4**) alters the spectral features (Figure 2, lower panel). The free base porphyrin triad also has been incorporated as a crossbar unit in a pentad array for solar light capture and charge separation [48].

In this paper, we describe the extension of the panchromatic triad for studies in light-harvesting. One design incorporates a single carboxylic acid for bioconjugation, such as in biohybrid assemblies wherein additional solar light capture is advantageous. A second design incorporates an isophthalic acid terminal unit for attachment to surfaces, such as metal oxides for studies of photoinduced charge injection. The syntheses rely on established rational methods and provide relatively direct access to lipophilic panchromatic triads each bearing a single attachment handle.

## 2. Results and Discussion

### 2.1. Synthesis of a Bioconjugatable Panchromatic Triad

**Scheme 1:**
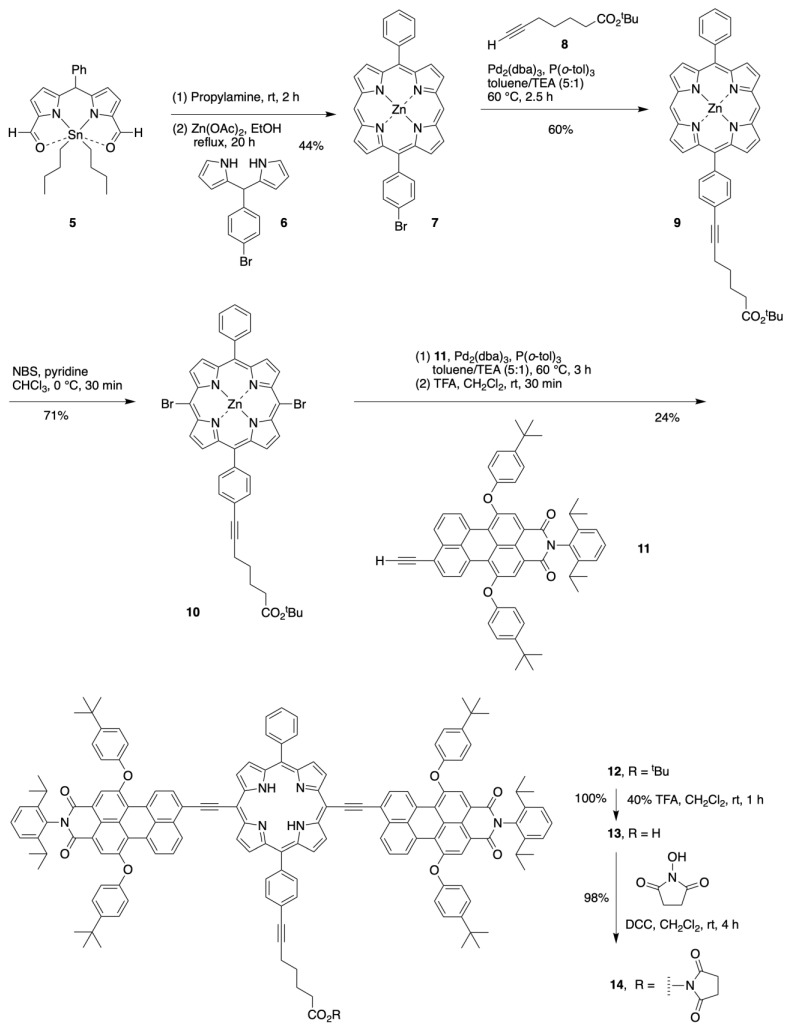
Synthesis of the bioconjugatable panchromatic absorber.

We sought to place a hexanoic acid chain on the central porphyrin for bioconjugation purposes. A *meso*-dibromo-substituted porphyrin building block was prepared for the construction of the bioconjugatable panchromatic triad. The core porphyrin contains an AB pattern of substituents with two open meso-positions and was prepared in a standard manner [51] from two dipyrromethanes (Scheme 1). The dibutyltin-complexed 1,9-diformyl-5-phenyldipyrromethane **5** [52] was treated with propylamine [51] and then reacted with 1,9-diunsubstituted 5-(4-bromophenyl)dipyrromethane **6** [53] in the presence of Zn(OAc)_2_ to afford the *trans*-AB zinc porphyrin **7**, which bears a bromine atom at a site for the introduction of a bioconjugatable tether. The bromo-porphyrin **7** thereafter reacted with ethynyl tether **8** [54] via a Sonogashira coupling reaction to form the bioconjugatable zinc porphyrin **9**. The Pd-mediated Sonogashira reaction [55] was carried out under copper-free conditions [56,57] using a solvent mixture of toluene and triethylamine (TEA). The bromination [58] of porphyrin **9** using *N*-bromosuccinimide (NBS) at 0 °C yielded the dibromoporphyrin building block **10**. The next step entailed attachment of two perylene-ethyne units.

Several points warrant comment concerning the choice of ethynyl-perylene for coupling with the dibromoporphyrin. The chosen perylene (**11**) bears two aryloxy groups (one in each bay region) and a 2,6-diisopropylphenyl group at the *N*-imide site, which together impart structural features that enable good solubility of the perylene in hydrocarbon solvents [46]. The ethyne is located at the perylene 9-position, a site of considerable electron density in the frontier molecular orbitals and a site known to afford substantial electronic communication upon covalent attachment to the porphyrin [42,45,46,47,48,49,50]. The ethynyl-perylene-monoimide **11** is an advanced functional dye that has emerged from extensive studies over several decades beginning with the pioneering work of Langhals [59,60,61,62,63,64,65]. A key distinction between **11** and many other rylene dyes [66,67,68] is the presence of a single imide versus two imides. The presence of two imides causes the resulting dye to be a good photooxidant, whereas the mono-imide is more electroneutral. The synthesis of **11** [46] follows methods [39,69,70,71] established earlier using a 2,5-di-*tert*-butylphenyl group at the *N*-imide site. The 2,6-diisopropylphenyl group of **11** is superior in not giving rise to stereoisomers as occur with the 2,5-di-*tert*-butylphenyl group (as in **1**) [60,61,65]. 

**Figure 3:**
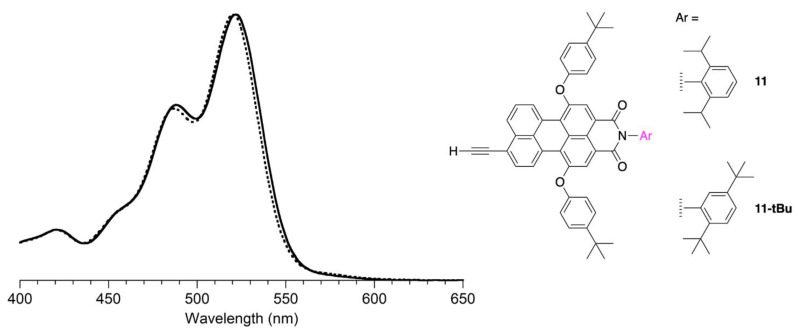
Absorption spectra (solid lines) in toluene at room temperature of ethynyl-perylene **11** (λ_abs_ 522 nm, ε_522 nm_ = 40,500 M^−1^cm^−1^, solid line) and ethynyl-perylene **11-tBu** (λ_abs_ 520 nm, dashed line).

The absorption spectra of ethynyl-perylene-monoimides bearing the 2,6-diisopropylphenyl group (**11**) or the 2,5-di-*tert*-butylphenyl (**11-tBu** [70,71]) are nearly identical, as shown in Figure 3. The similarity of spectra upon use of either solubilization motif is consistent with observation that the 2,6-diisopropyl *versus* 2,5-di-*tert*-butyl group has insignificant effects on perylene photophysics [40]. The similar spectra and photophysics are attributed to the presence of a node at the perylene-imide nitrogen atom in both the highest occupied molecular orbital (HOMO) and the lowest unoccupied molecular orbital (LUMO) [61]. Thus, the changes in porphyrin substituents from mesityl to *p*-tolyl groups, and perylene *N*-imide from a 2,5-di-*tert*-butylphenyl to a 2,6-diisopropylphenyl group, facilitate synthesis and chemical characterization but have hardly any effects on spectral and photophysical properties. 

The Sonogashira coupling [55] reaction of ethynyl-perylene **11** and dibromoporphyrin **10** was carried out under copper-free [56,57] Pd-mediated conditions (as in the reaction of **7** and **8**). Such a reaction of an ethynylphenyl-chromophore and a meso-bromoporphyrin is facile and has been carried out under nearly identical conditions to form porphyrin-phenylethynyl-porphyrin dyads [44]. The rationale for copper-free conditions in the synthesis was to avoid any transmetalation of the zinc porphyrin given (1) the avidity of porphyrins for Cu(II); (2) the very short-lived singdoublet excited-state lifetime of copper porphyrins (marked by the absence of fluorescence); and (3) the subsequent difficulty of removing copper from porphyrins, unlike the facile removal of zinc achieved upon treatment with mild acid. Thus, the subsequent acid-mediated demetalation of zinc [42] with trifluoroacetic acid (TFA) afforded the target panchromatic triad **12**. Purification was achieved via a three-chromatography sequence that includes use of size-exclusion chromatography (SEC) [42,72]. Removal of the *tert*-butyl protecting group using 40% TFA [73] in CH_2_Cl_2_ gave the carboxy-triad **13** (Scheme 1). For bioconjugation purposes, **13** was further transformed to an *N*-hydroxysuccinimide ester **14** via reaction [74] with *N*-hydroxysuccinimide in the presence of *N,N*-dicyclohexylcarbodiimide (DCC).

### 2.2. Synthesis of a Panchromatic Triad for Surface Attachment

**Chart 2:**
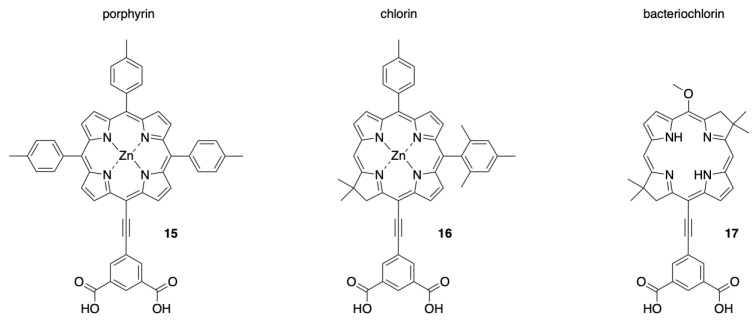
Tetrapyrrole dyes bearing an isophthalate tether.

We previously prepared a set of tetrapyrrole macrocycles (**15–17**) bearing an isophthalic acid tether [75] for surface attachment (Chart 2) [76]. The present work extends this design motif. 

**Scheme 2:**
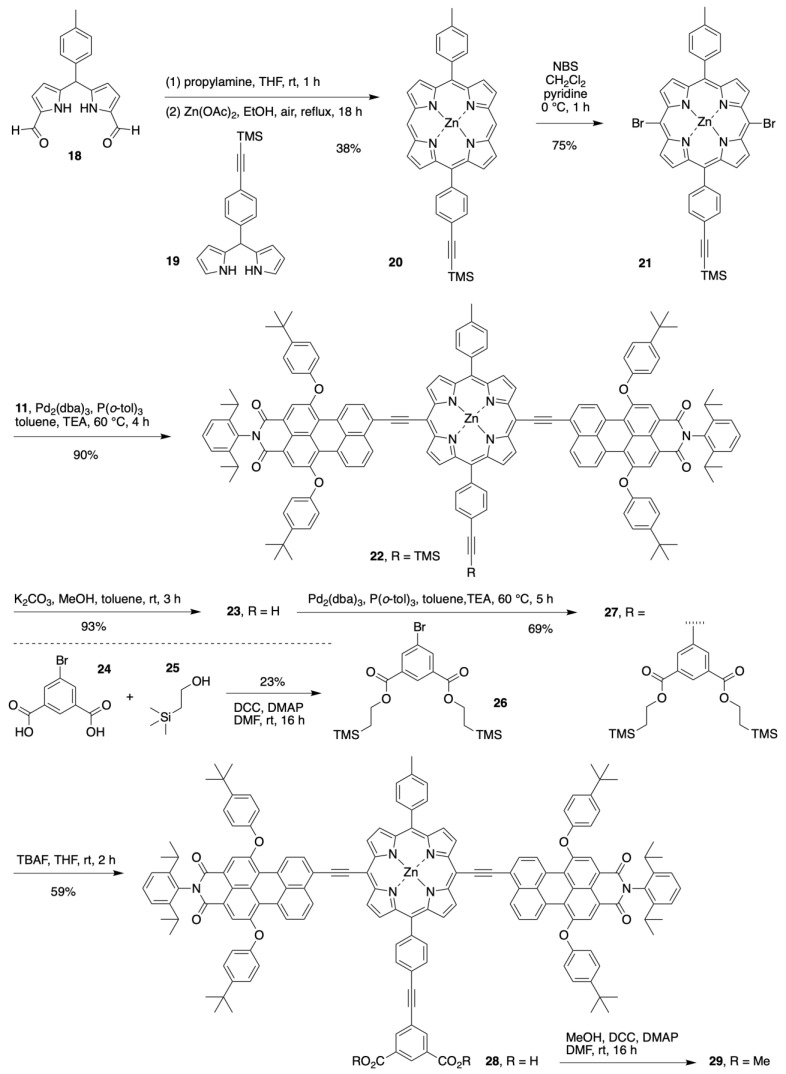
Synthesis of a perylene-porphyrin-perylene triad (**28**) for surface attachment.

**Figure 4:**
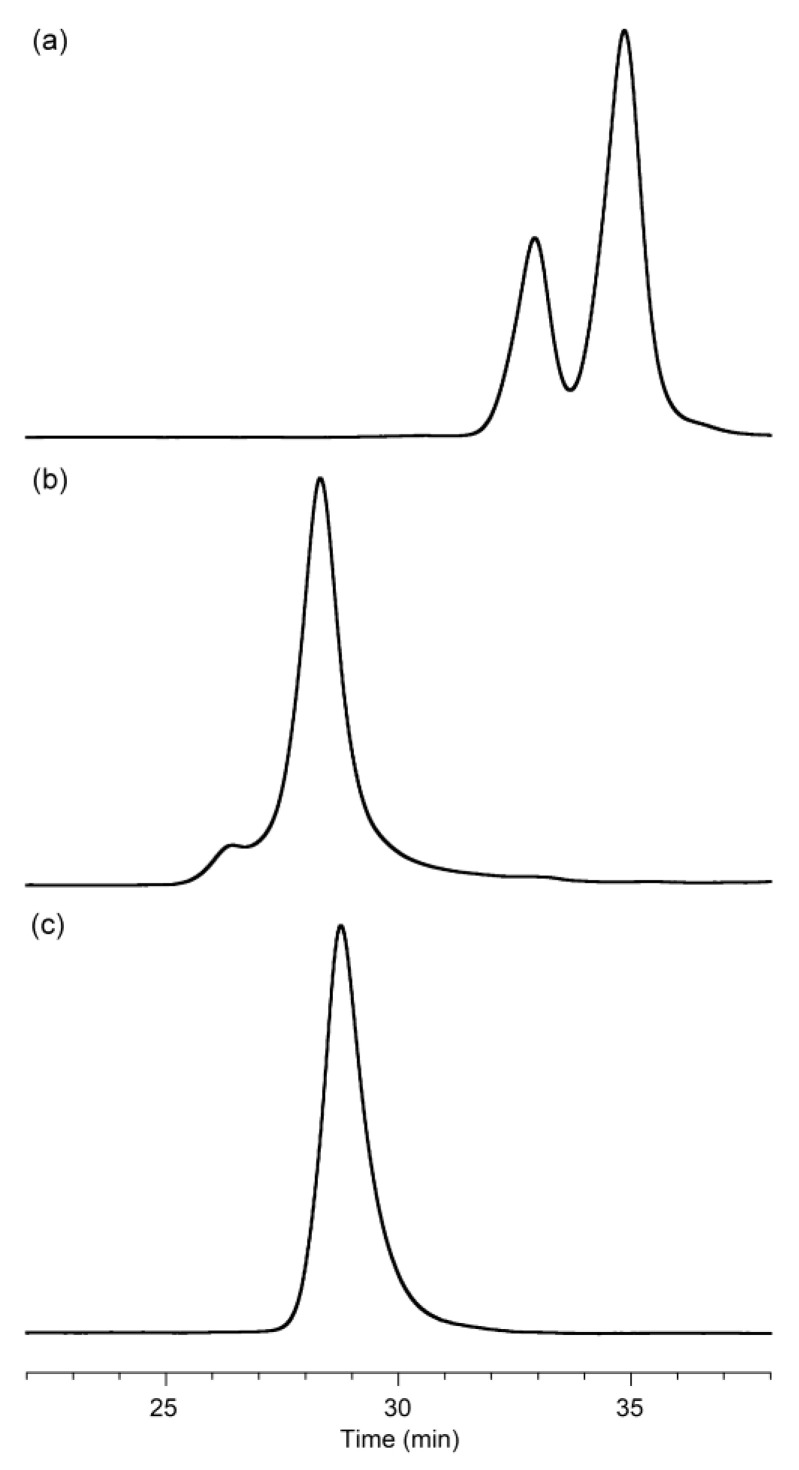
Analytical SEC traces (λ_det_ = 550 nm) for the synthesis of a triad array **22**. (**a**): Mixture of starting materials **11** and **21**. (**b**): Crude mixture after reaction for 4 h. (**c**): Purified triad **22**.

The synthesis of the core porphyrin follows that shown for the bioconjugatable triad. Thus, the 1,9-formyldipyrromethane **18** was reacted with propylamine [51] to form the bis(imine), which was then treated with the complementary dipyrromethane **19** [77] to afford the zinc porphyrin **20** in 38% yield (Scheme 2). The bromination [58] of zinc porphyrin **20** afforded the corresponding dibromo zinc porphyrin **21** in 75% yield. Sonogashira coupling [55] of the dibromo zinc porphyrin **21** and ethynyl-perylene **11** under copper-free conditions [56,57] afforded the triad **22** bearing two perylenes and one zinc porphyrin in 90% yield (~200 mg). The reaction progress was monitored by analytical SEC [72], as has been done previously with other panchromatic arrays [42,46]. The analytical SEC traces with absorption spectral determination show the starting materials (Figure 4, panel a), crude mixture after reaction for several hours (panel b), and the purified triad **22** (panel c) following preparative purification using the three-column chromatography process. The cleavage of the trimethylsilyl (TMS) group [48] of **22** afforded triad **23** bearing a free ethynyl group in 93% yield.

The surface-attachment motif was prepared through the DCC-mediated condensation of 5-bromoisophthalic acid (**24**) and 2-(trimethylsilyl)ethanol (**25**) in *N,N*-dimethylformamide (DMF) to give the protected 5-bromo-isophthalate **26** in 23% yield (Scheme 2). The Sonogashira coupling [55] of ethynyl-triad **23** and isophthalate **26** under copper-free conditions [56,57] afforded the protected tethered triad **27** in 69% yield. The removal of the 2-trimethylsilylethyl group [76] of **27** upon treatment with tetrabutylammonium fluoride (TBAF) in tetrahydrofuran (THF) afforded isophthalate-tethered triad **28** in 59% yield. While protected triad **27** was fully characterized, all efforts to characterize **28** by NMR spectroscopy and mass spectrometry were unsuccessful. For confirmation purposes, a small portion of **28** was treated with methanol in the presence of DCC and and 4-dimethylaminopyridine (DMAP) to form the corresponding dimethyl ester **29**, which gave the expected mass peak upon matrix-assisted laser-desorption ionization mass spectrometry (MALDI-MS) analysis. 

**Scheme 3:**
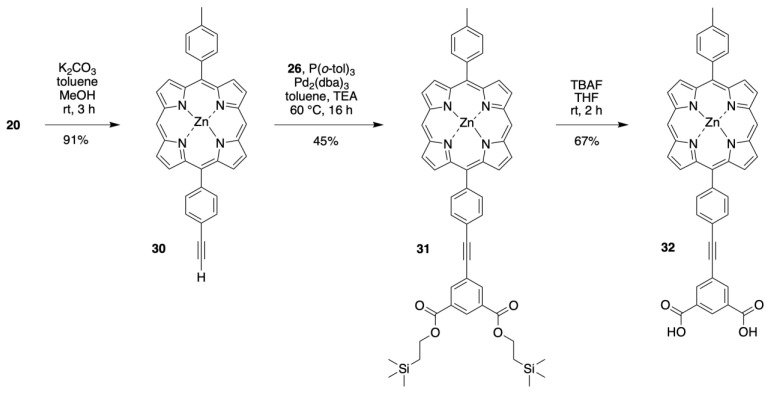
Synthesis of tethered porphyrin **32** lacking perylenes.

A tethered porphyrin analogue lacking the two perylene-ethynyl groups was prepared. The cleavage of the TMS group [48] of zinc porphyrin **20** afforded zinc porphyrin **30** bearing an ethynyl group in 91% yield (Scheme 3). The Sonogashira coupling [55] of **30** and isophthalate **26** under copper-free conditions [56,57] afforded the protected tethered porphyrin **31** in 45% yield. The removal of the 2-trimethylsilylethyl groups [76] of **31** upon treatment with TBAF afforded isophthalate-tethered porphyrin **32** in 78% yield.

### 2.3. Chemical Characterization

**Figure 5:**
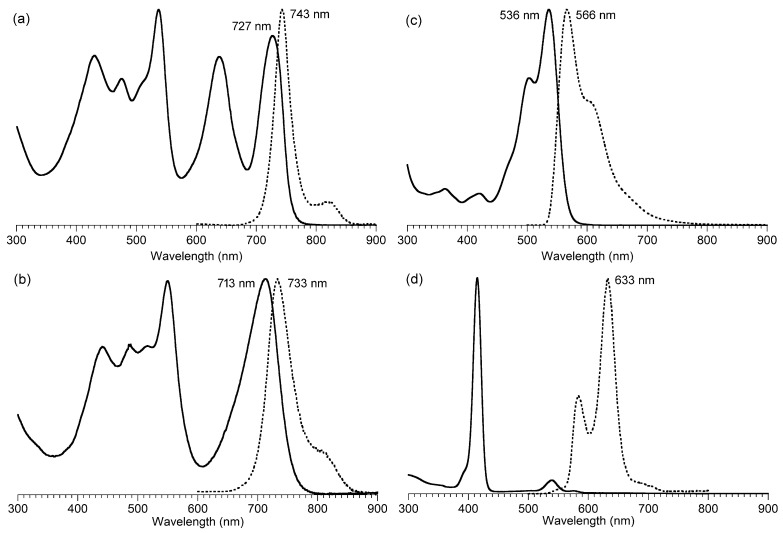
Absorption spectra (solid lines) and emission spectra (dashed lines) in toluene at room temperature (normalized displays). (**a**): free base porphyrin carboxy-triad **13** shows λ_abs_ 537 nm, ε_537 nm_ = 67,800 M^−1^cm^−1^; fluorescence λ_em_ 743 nm upon λ_ex_ = 521 nm). (**b**): zinc porphyrin isophthalate-triad **27** shows λ_abs_ 548 nm, ε_548 nm_ = 99,800 M^−1^cm^−1^; fluorescence λ_em_ 733 nm upon λ_ex_ = 549 nm). (**c**): perylene **33** (fluorescence λ_em_ 566 nm upon λ_ex_ ~500 nm). (**d**): *trans*-AB zinc porphyrin **31** (fluorescence λ_em_ 633 nm upon λ_ex_ = 415 nm).

**Chart 3:**
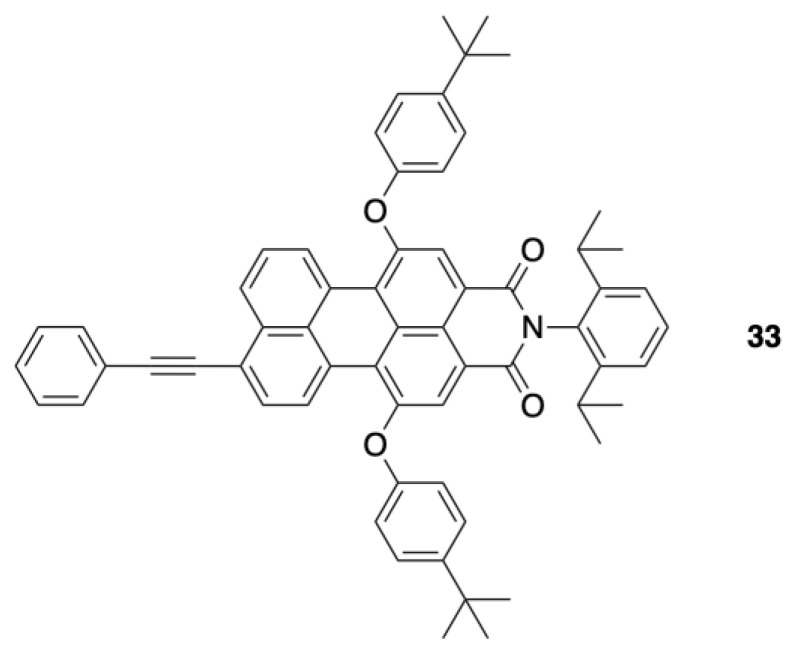
Benchmark perylene.

The triads were generally characterized by ^1^H NMR spectroscopy, absorption spectroscopy, and MALDI-MS analysis. Limited solubility precluded the collection of ^13^C NMR spectra for a number of the triads as well as other compounds. Accurate mass data were obtained by electrospray ionization mass spectrometry (ESI-MS) where possible. The absorption and fluorescence spectra of two tethered triads are shown in Figure 5. The carboxy-triad **13** contains a free base porphyrin, whereas the isophthalate-triad **27** contains a zinc porphyrin. The absorption spectrum of carboxy-triad **13** (panel a) is nearly identical to that of the untethered triad **4** shown in Figure 2. The absorption spectrum of isophthalate-triad **27** (panel b) is nearly identical to that of the untethered triad **Zn4** shown in Figure 2. For comparison purposes, the absorption and fluorescence spectra of a benchmark perylene (**33**) [46] and the *trans*-AB zinc porphyrin **31** are shown in panels c and d, respectively. Perylene **33** includes a phenyl group at the terminus of the ethyne and a 2,6-diisopropylphenyl substituent at the *N*-imide position (Chart 3) and is displayed correctly in the original report of synthesis and characterization [46], but is shown incorrectly with the 2,5-di-*tert*-butylphenyl substituent at the *N*-imide position in a subsequent report [50]. Molar absorption coefficient values are reported for triads **13**, **23**, **27**, and **28**, as well as for benchmark ethynyl-perylene-monoimide **11**. The Φ_f_ values measured in toluene for triad **13**, triad **27**, perylene **33,** and porphyrin **31** are 0.21, 0.24, 0.94 [50], and 0.011, respectively. For comparison, the Φ_f_ values for free base porphyrin triad **4** and zinc porphyrin triad **Zn4** are 0.26 and 0.30, respectively [45]. The fluorescence emission of each triad exhibits (as expected) the general spectral features of a porphyrin but with an enhanced quantum yield. ^1^H and ^13^C{^1^H} NMR spectra, mass spectra, and absorption spectra (where available) for new compounds are provided in the Supplementary Materials.

## 3. Materials and Methods

### 3.1. General Methods

All chemicals obtained commercially were used as received unless noted otherwise. Reagent-grade solvents (CH_2_Cl_2_, hexanes, methanol, toluene, ethyl acetate) and HPLC-grade solvents (toluene, CH_2_Cl_2,_ hexanes) were used as received. THF was freshly distilled from sodium/benzophenone ketyl and used immediately. MALDI-MS was performed with the matrix 1,4-bis(5-phenyl-2-oxazol-2-yl)benzene (POPOP) [78] or α-cyano-4-hydroxycinnamic acid (α-CHCA) as noted. ESI-MS data were obtained in positive-ion mode unless noted otherwise. Known building blocks **5** [52], **6** [53], **8** [54], **11** [46], **18** [51], and **19** [77] were prepared via reported methods.

### 3.2. Purification Following Sonogashira Coupling Reactions

Following a three-chromatography procedure [42,70], reaction mixtures of arrays were first chromatographed with adsorption column chromatography (flash silica, Baker) to remove catalysts and ligands from the coupling reaction. Then, preparative-scale size exclusion chromatography (SEC) was performed using BioRad Bio-Beads S-X1. A preparative-scale glass column (4.3 × 53 cm) was packed using BioRad Bio-Beads S-X1 in HPLC grade toluene. The chromatography was performed with gravity flow (~0.2 mL/min). Thereafter, a subsequent adsorption column chromatography (flash silica, Baker) procedure was performed (with HPLC-grade CH_2_Cl_2_ and hexanes unless noted otherwise) to remove material that may have leached from the SEC resin. 

The preparative purification procedure is generally most effective when the reaction affords a change in size; e.g., in instances where the product is substantially larger than the starting materials. Such is the case of forming triads via Sonogashira coupling procedures as described herein. The purification protocol is applicable to both zinc and free base porphyrins. Here, all Sonogashira coupling reactions were carried out under anaerobic, copper-free conditions [56,57] using zinc porphyrins in relatively dilute solution, as required for homogeneous solubilization of each porphyrin reactant. 

Analytical-scale SEC was performed to monitor the purification of arrays [42,56]. Analytical SEC columns (styrene-divinylbenzene copolymer) were purchased from Polymer Laboratories. Analytical SEC was performed with a Hewlett-Packard 1100 HPLC using PLgel 100 Å, Plgel 500 Å, and PLgel 1000 Å columns (each ~30-cm in length) in series, eluting with toluene (flow rate = 1.0 mL/min). Sample detection was achieved by absorption spectroscopy using a diode array detector with quantitation at 422, 521, 638, and 726 nm (±8 nm band width), which best captures the peaks of the arrays. In other cases, analytical SEC was performed using PLgel 50 Å, PLgel 100 Å × 2, and PLgel 500 Å columns (each ~30-cm in length) in series, eluting with THF (flow rate = 0.8 mL/min) at room temperature. Sample detection was achieved by absorption spectroscopy using a diode array detector with quantitation at 440, 488, 515, 550, and 710 nm.

### 3.3. Synthesis and Characterization

*Zinc(II)-5-(4-Bromophenyl)-15-phenylporphyrin* (**7**). Following a general procedure [51], a solution of **5** (126 mg, 247 μmol) in propylamine (0.4 mL, 5 mmol) was stirred at room temperature for 2 h. Then the mixture was concentrated and dried at high vacuum for 5 min. The resulting solid was dissolved in ethanol (30 mL) and then treated with **6** (75.0 mg, 250 μmol) and zinc acetate (0.52 g, 25 mmol). The mixture was refluxed at 90 °C open to the air for 20 h. The mixture was allowed to cool to room temperature and then was concentrated. Column chromatography [silica, hexanes/CH_2_Cl_2_ (1:2) to CH_2_Cl_2_] afforded a pink solid (67 mg, 45%): ^1^H NMR (THF-*d*_8_, 400 MHz) δ 10.29 (s, 2H), 9.44–9.41 (m, 4H), 9.04–9.02 (m, 4H), 8.26–8.24 (m, 2H), 8.18–8.16 (m, 2H), 7.98–7.96 (m, 2H), 7.80–7.78 (m, 3H); ^13^C{^1^H} NMR (THF-*d*_8_, 100 MHz) δ 150.7, 149.8, 136.4, 134.8, 132.0, 131.7, 131.5, 129.7, 127.3, 126.5, 105.8; MALDI-MS (POPOP) obsd 603.8, calcd 603.0 [M + H]^+^; ESI-MS obsd 602.0089, calcd 602.0079 (M^+^), M = C_32_H_19_BrN_4_Zn; λ_abs_ (toluene) 413, 538, 573 nm. 

*Zinc(II)-5-[4-(7-tert-Butoxy-7-oxohept-1-ynyl)phenyl]-15-phenylporphyrin* (**9**). Following a general procedure [56,57], a mixture of toluene/triethylamine (5:1, *v*:*v*) was deaerated with a continuous steam of argon for 1 h. A mixture of zinc porphyrin **7** (22 mg, 36 µmol), P(*o*-tol)_3_ (14 mg, 46 μmol), and Pd_2_(dba)_3_ (5 mg, 6 µmol) was placed into a 100 mL Schlenk flask and evacuated under high vacuum for 10 min. The flask was refilled with argon thereafter, and the procedure was repeated three times. Then the degassed solvent (10 mL) was added to the flask, whereupon three freeze–pump–thaw cycles were performed. The mixture was allowed to warm to temperature. Ethynyl coupling partner **8** (19 mg, 0.10 mmol) was added dropwise to the mixture under a continuous stream of argon. The mixture was then stirred for 3 h at 60 °C. The mixture was allowed to cool to room temperature and then was quenched by the addition of water. The mixture was extracted with CH_2_Cl_2_. The organic layer was washed with water, dried (Na_2_SO_4_), concentrated and chromatographed [silica, hexanes/CH_2_Cl_2_ (1:2) to CH_2_Cl_2_] to afford a dark purple solid (47 mg, 60%): ^1^H NMR (THF-*d*_8_, 400 MHz) δ 10.46 (s, 2H), 9.61–9.58 (m, 4H), 9.24–9.21 (m, 4H), 8.45–8.37 (m, 4H), 8.01–7.96 (m, 5H), 2.81 (t, *J* = 6.8 Hz, 2 H), 2.55 (t, *J* = 6.8 Hz, 2H), 2.08 (q, *J* = 7.2 Hz, 2H), 2.00–1.95 (m, 2H), 1.69 (s, 9H); ^13^C{^1^H} NMR (THF-*d*_8_, 100 MHz) δ 187.5, 172.0, 150.1, 149.9, 149.8, 149.7, 143.6, 143.0, 142.4, 135.6, 134.9, 134.8, 131.9, 131.7, 131.6, 131.5, 130.2, 129.7, 128.9, 128.4, 127.3, 126.5, 125.8, 123.5, 119.7, 118.8, 105.8, 90.6, 81.1, 79.3, 34.8, 28.6, 19.2; MALDI-MS (POPOP) obsd 705.2, calcd 705.2 [M + H]^+^; ESI-MS obsd 704.2134, calcd 704.2124 (M^+^), M = C_43_H_36_N_4_O_2_Zn; λ_abs_ (toluene) 413, 539, 573 nm. 

*Zinc(II)-10,20-Dibromo-5-[4-(7-tert-butoxy-7-oxohept-1-ynyl)phenyl]-15-phenylporphyrin* (**10**). Following a general procedure [58], a solution of **9** (47 mg, 67 μmol) in CHCl_3_ (23 mL) was stirred at 0 °C in ice bath, then treated with NBS (38 mg, 21 μmol). The mixture was stirred at 0 °C for 30 min, whereupon acetone was added to quench the reaction. Then the mixture was washed with water and extracted with CH_2_Cl_2_. The organic extract was dried (Na_2_SO_4_), concentrated, and chromatographed [silica, CH_2_Cl_2_] to afford a green solid (41 mg, 71%): ^1^H NMR (THF-*d*_8_, 400 MHz) δ 9.69–9.67 (m, 4H), 8.90–8.87 (m, 4H), 8.19 (d, *J* = 6.4 Hz, 2H), 8.13 (d, *J* = 8.0 Hz, 2H), 7.83–7.79 (m, 5H), 2.64 (t, *J* = 6.8 Hz, 2H), 2.38 (t, *J* = 8.0 Hz, 2H), 1.91 (p, *J* = 6.8 Hz, 2H), 1.82 (p, *J* = 7.2 Hz, 2H), 1.52 (s, 9H); ^13^C{^1^H} NMR (THF-*d*_8_, 100 MHz) δ 172.0, 151.0, 150.7, 150.2, 142.9, 142.2, 134.7, 134.6, 133.2, 133.0, 132.9, 132.7, 129.6, 127.7, 126.6, 123.9, 122.3, 121.5, 104.6, 90.9, 80.9, 79.3, 34.8, 28.6, 27.6, 19.2; ESI-MS obsd 860.0335, calcd 860.03245 (M^+^), M = C_43_H_34_N_4_O_2_Br_2_Zn; λ_abs_ (toluene) 429, 539, 598 nm.

*5-[4-(7-tert-Butoxy-7-oxohept-1-ynyl)phenyl]-10,20-bis[2-(3,4-(N-(2,6-diisopropylphenyl)iminodicarbonyl)-1,6-bis(4-tert-butylphenoxy)perylen-9-yl)ethynyl]-15-phenylporphyrin* (**12**). Following a standard procedure [44,46,56,57], a mixture of zinc dibromoporphyrin **10** (3.0 mg, 3.5 μmol), ethynyl-perylene **11** (6.0 mg, 7.5 μmol), P(*o*-tol)_3_ (2.2 mg, 7.2 μmol), and Pd_2_(dba)_3_ (0.8 mg, 1.0 µmol) in degassed toluene/triethylamine (2.2 mL, 5:1, *v*:*v*) was stirred at 60 °C for 3 h. The mixture was allowed to cool at room temperature. The mixture was then washed with water and extracted with CH_2_Cl_2_. The organic extract was dried (Na_2_SO_4_) and concentrated. The resulting solid was dissolved in CH_2_Cl_2_ (2.0 mL) and treated with TFA (14 μL). The mixture was stirred at room temperature for 1 h, whereupon excess triethylamine was added to quench the reaction. The solution was then washed with water, dried (Na_2_SO_4_), concentrated, and chromatographed using the standard three-chromatography procedure to afford a black solid (2.3 mg, 29%): ^1^H NMR (CDCl_3_, 300 MHz) δ 9.78–9.73 (m, 4H), 9.31 (d, *J* = 7.8 Hz, 2H), 9.20 (d, *J* = 8.1 Hz, 2H), 9.06 (d, *J* = 8.1 Hz, 2H), 8.89–8.83 (m, 4H), 8.34 (s, 2H), 8.26–8.04 (m, 8H), 7.91 (d, *J* = 8.1 Hz, 2H), 7.84–7.80 (m, 3H), 7.74–7.68 (m, 2H), 7.46–7.39 (m, 10H), 7.32 (d, *J* = 7.8 Hz, 4H), 7.08 (d, *J* = 9.0 Hz, 4H), 6.98–6.95 (m, 4H), 2.77 (q, *J* = 6.6 Hz, 4H), 2.63 (t, *J* = 6.8 Hz, 2H), 2.39 (t, *J* = 7.2 Hz, 2H), 1.92 (q, *J* = 7.2 Hz, 2H), 1.83 (q, *J* = 6.6 Hz, 2H), 1.52 (s, 9H), 1.34 (s, 36H), 1.18 (d, *J* = 6.6 Hz, 24H), –2.01 (br, 2H); MALDI-MS (POPOP) obsd 2242.7, calc 2241.0 [M + H]^+^, M = C_155_H_136_N_6_O_10_; λ_abs_ (toluene) 431, 476, 537, 638, 727 nm.

*5-[4-(7-Hydroxy-7-oxohept-1-ynyl)phenyl]-10,20-bis[2-(3,4-(N-(2,6-diisopropylphenyl)iminodicarbonyl)-1,6-bis(4-tert-butylphenoxy)perylen-9-yl)ethynyl]-15-phenylporphyrin* (**13**). A solution of the *tert*-butyl protected triad **12** (2.3 mg, 1.0 μmol) in CH_2_Cl_2_ (1.2 mL) was treated with TFA (0.8 mL). The mixture was stirred at room temperature for 1 h, whereupon excess triethylamine was added to quench the reaction. The mixture was then washed with water, dried (Na_2_SO_4_), and concentrated to afford a black solid (2.2 mg, 100%): ^1^H NMR (CDCl_3_, 300 MHz) δ 9.73–9.66 (m, 4H), 9.24 (d, *J* = 8.1 Hz, 2H), 9.18 (d, *J* = 8.4 Hz, 2H), 8.99 (d, *J* = 8.4 Hz, 2H), 8.83–8.78 (m, 4H), 8.32–8.28 (m, 2H), 8.16–8.07 (m, 8H), 7.96 (d, *J* = 8.4 Hz, 2H), 7.83–7.76 (m, 3H), 7.66–7.61 (m, 2H), 7.43–7.38 (m, 10H), 7.31 (d, *J* = 7.5 Hz, 4H), 7.06 (d, *J* = 9.0 Hz, 4H), 6.99 (d, *J* = 9.0 Hz, 4H), 2.77 (q, *J* = 6.6 Hz, 4H), 2.65 (t, *J* = 6.6 Hz, 2H), 2.41 (t, *J* = 7.5 Hz, 2H), 1.86 (q, *J* = 7.2 Hz, 2H), 1.83 (q, *J* = 6.9 Hz, 2H), 1.33 (s, 36H), 1.17 (d, *J* = 7.2 Hz, 24H), –1.97 (br, 2H), a signal due to the carboxylic acid was not observed; MALDI-MS (POPOP) obsd 2187.9, calc 2185.0 (M^+^), M = C_151_H_128_N_6_O_10_; λ_abs_ (toluene) 430, 476, 537, 638, 727 nm, ε_537 nm_ = 67,800 M^−1^cm^−1^, ε_727 nm_ = 56,100 M^−1^cm^−1^; λ_em_ (toluene, λ_ex_ = 521 nm) 743 nm.

*5-[4-(7-(N-succinimidooxy)-7-oxohept-1-ynyl)phenyl]-10,20-bis[2-(3,4-(N-(2,6-diisopropylphenyl)iminodicarbonyl)-1,6-bis(4-tert-butylphenoxy)perylen-9-yl)ethynyl]-15-phenylporphyrin* (**14**). Following a general procedure [74], a solution of carboxy-triad **13** (5.5 mg, 2.5 μmol), *N*-hydroxysuccinimide (2.8 mg, 25 μmol), and DCC (5.0 mg, 25 μmol) in CH_2_Cl_2_ (0.25 mL) was stirred under argon at room temperature for 4 h. Then the mixture was washed with water, dried (Na_2_SO_4_), concentrated, and chromatographed [silica, CH_2_Cl_2_] to afford a black solid (5.6 mg, 98%): ^1^H NMR (CDCl_3_, 300 MHz) *δ* 9.78 (d, *J* = 4.5 Hz, 4H), 9.47–9.42 (m, 4H), 9.10 (d, *J* = 8.4, 2H), 8.89–8.87 (m, 4H), 8.36 (d, *J* = 4.2 Hz, 4H), 8.31 (d, *J* = 8.4 Hz, 2H), 8.13 (d, *J* = 8.1 Hz, 2H), 7.86–7.80 (m, 7H), 7.46–7.43 (m, 10H), 7.31 (d, *J* = 7.8 Hz, 4H), 7.14–1.09 (m, 8H), 2.87 (s, 4H), 2.84–2.71 (m, 6H), 2.68 (t, *J* = 6.6 Hz, 2H), 2.17–2.06 (m, 2H), 1.95–1.85 (m, 4H), 1.35 (s, 36H), 1.17 (d, *J* = 6.6 Hz, 24H), –1.78 (br, 2H); MALDI-MS (POPOP) obsd 2283.5, calc 2283.0 (M^+^), M = C_155_H_131_N_7_O_12_.

*Zinc(II) 15-p-Tolyl-5-[4-(2-(trimethylsilyl)ethynyl)phenyl]porphyrin* (**20**). Following a reported method [51], a solution of diformyldipyrromethane **18** (438 mg, 1.5 mmol) in THF (5 mL) was treated with propylamine (1.8 g, 30 mmol) at room temperature for 1 h. The solution was concentrated to dryness. The resulting solid was dissolved in ethanol (150 mL) and treated with dipyrromethane **19** (480 mg, 1.5 mmol) and Zn(OAc)_2_ (2.8 g, 15 mmol). The mixture was refluxed open to the air for 18 h. The reaction mixture was allowed to cool to room temperature, then concentrated to dryness and purified by chromatography [silica, hexanes/CH_2_Cl_2_ (4:1 to 1:1)] to afford a purple solid (351 mg, 38%): ^1^H NMR (600 MHz, CDCl_3_) *δ* 10.28 (s, 2H), 9.41 (d, *J* = 4.4 Hz, 4H), 9.16 (d, *J* = 4.4 Hz, 2H), 9.08 (d, *J* = 4.4 Hz, 2H), 8.19 (d, *J* = 7.7 Hz, 2H), 8.14 (d, *J* = 7.4 Hz, 2H), 7.91 (d, *J* = 7.7 Hz, 2H), 7.61 (d, *J* = 7.4 Hz, 2H), 2.75 (s, 3H), 0.41 (s, 9H); ^13^C{^1^H} NMR (151 MHz, CDCl_3_) *δ* 150.2, 149.6, 149.4, 149.3, 142.9, 137.1, 134.5, 134.4, 132.6, 132.0, 131.8, 131.6, 130.2, 127.3, 122.2, 120.4, 118.9, 106.2, 21.4; MALDI-MS (α-CHCA) obsd 634.2, calcd 634.2 (M^+^), M = C_38_H_30_N_4_SiZn; λ_abs_ (toluene) 414, 540 nm. 

*Zinc(II) 5,15-Dibromo-20-p-tolyl-10-[4-(2-(trimethylsilyl)ethynyl)phenyl]porphyrin* (**21**). Following a standard bromination method [58], a solution of zinc porphyrin **20** (127 mg, 0.20 mmol) in CH_2_Cl_2_ (70 mL) containing pyridine (1.4 mL) was treated with NBS (85 mg, 0.48 mmol) at 0 °C for 1 h. The reaction mixture was quenched by the addition of acetone (3.0 mL). The mixture was washed with water (80 mL) and brine (80 mL), dried, concentrated to dryness, and purified by chromatography [silica, hexanes/CH_2_Cl_2_ (10:1 to 3:1)] to afford a greenish purple solid (119 mg, 75%): ^1^H NMR (600 MHz, CDCl_3_) *δ* 9.68–9.62 (m, 4H), 8.89 (d, *J* = 4.6 Hz, 2H), 8.82 (d, *J* = 4.6 Hz, 2H), 8.09 (d, *J* = 7.9 Hz, 2H), 8.02 (d, *J* = 7.5 Hz, 2H), 7.86 (d, *J* = 7.9 Hz, 2H), 7.55 (d, *J* = 7.4 Hz, 2H), 2.72 (s, 3H), 0.39 (s, 9H); MALDI-MS (α-CHCA) obsd 790.2, calcd 790.0 (M^+^), M = C_38_H_28_Br_2_N_4_SiZn; λ_abs_ (toluene) 432, 560, 600 nm. 

*Zinc(II) 10,20-Bis[2-(3,4-(N-(2,6-diisopropylphenyl)iminodicarbonyl)-1,6-bis(4-tert-butylphenoxy)perylen-9-yl)ethynyl]-5-p-tolyl-15-(4-(2-trimethylsilylethynyl)phenyl)porphyrin* (**22**). Following a standard procedure [44,46,56,57], a Schlenk flask containing zinc porphyrin **21** (79 mg, 0.10 mmol), ethynyl-perylene **11** (192 mg, 0.24 mmol), and tri(*o*-tolyl)phosphine (73 mg, 0.24 mmol) was flushed with argon, treated with degassed toluene/TEA (50 mL, 5:1, *v*:*v*), and subjected to three freeze–pump–thaw cycles. The mixture was then treated with Pd_2_(dba)_3_ (27 mg, 0.030 µmol) and subjected to two additional freeze–pump–thaw cycles. The resulting mixture was heated to 60 °C for 4 h. The crude mixture was purified following the standard three-chromatography procedure [silica (hexanes/CH_2_Cl_2_ (2:1 to 1:1)), SEC (toluene), silica (hexanes to hexanes/CH_2_Cl_2_ (1:1))] to afford a purple solid (161 mg, 72%): ^1^H NMR (600 MHz, CDCl_3_) δ 9.80–9.69 (m, 4H), 9.11–8.98 (m, 4H), 8.87 (d, *J* = 4.2 Hz, 2H), 8.81 (d, *J* = 4.3 Hz, 2H), 8.58 (s, 2H), 8.26 (s, 4H), 8.06–7.99 (m, 2H), 7.99–7.93 (m, 2H), 7.91–7.85 (m, 4H), 7.82 (br, 2H), 7.58 (d, *J* = 7.2 Hz, 2H), 7.47–7.38 (m, 8H), 7.31 (d, *J* = 8.1 Hz, 4H), 7.27 (s, 2H), 6.97 (d, *J* = 8.3 Hz, 4H), 6.49 (s, 4H), 2.82–2.72 (m, 7H), 1.35 (s, 18H), 1.30 (s, 18H), 1.23–1.13 (m, 24H), 0.43 (s, 9H); MALDI-MS (α-CHCA) obsd 2232.7, calcd 2232.9 (M^+^), M = C_150_H_128_N_6_O_8_SiZn; λ_abs_ (toluene) 440, 488, 515, 549, 706 nm. 

*Zinc(II) 10,20-Bis[2-(3,4-(N-(2,6-diisopropylphenyl)iminodicarbonyl)-1,6-bis(4-tert-butylphenoxy)perylen-9-yl)ethynyl]-5-p-tolyl-15-(4-ethynylphenyl)porphyrin* (**23**). A solution of triad **22** (197 mg, 88 µmol) in toluene (75 mL) and methanol (75 mL) was treated with K_2_CO_3_ (1.21 g, 8.8 mmol) at room temperature for 3 h. The reaction mixture was poured into water (300 mL) and extracted with CH_2_Cl_2_ (200 mL × 2). The combined organic extract was washed with brine (200 mL), dried, and concentrated to dryness to afford a dark red solid (178 mg, 93%): ^1^H NMR (600 MHz, CDCl_3_) *δ* 9.81 (d, *J* = 4.4 Hz, 4H), 9.24 (s, 2H), 9.12 (d, *J* = 8.0 Hz, 2H), 8.96 (d, *J* = 4.4 Hz, 2H), 8.88 (d, *J* = 4.4 Hz, 2H), 8.32 (s, 4H), 8.16 (d, *J* = 8.0 Hz, 2H), 8.06 (d, *J* = 7.5 Hz, 2H), 8.03 (s, 2H), 7.99 (d, *J* = 7.2 Hz, 2H), 7.93 (d, *J* = 7.6 Hz, 2H), 7.65–7.59 (m, 4H), 7.46–7.42 (m, 6H), 7.34 (d, *J* = 8.6 Hz, 4H), 7.31 (d, *J* = 8.0 Hz, 4H), 7.05 (d, *J* = 8.5 Hz, 4H), 6.73 (s, 4H), 3.37 (s, 1H), 2.80–2.74 (m, 7H), 1.35 (s, 18H), 1.32 (s, 18H), 1.21–1.13 (m, 24H); MALDI-MS (α-CHCA) obsd 2160.9 (M^+^), calcd 2160.8 (M^+^), M = C_147_H_120_N_6_O_8_Zn; λ_abs_ (toluene) 440, 488, 515, 548, 706 nm, ε_548 nm_ = 103,000 M^−1^cm^−1^.

*Bis(2-(trimethylsilyl)ethyl) 5-bromoisophthalate* (**26**). A solution of 5-bromoisophthalic acid (**24**, 490 mg, 2.0 mmol) and 2-(trimethylsilyl)ethanol (**25**, 497 mg, 4.2 mmol) in DMF (12 mL) was treated with DCC (866 mg, 4.2 mmol) and DMAP (489 mg, 4.0 mmol) at room temperature for 16 h. The reaction mixture was diluted with ethyl acetate (80 mL) and filtered. The filtrate was washed with water (50 mL) and a saturated NH_4_Cl aqueous solution (50 mL), dried, concentrated to dryness, and then purified by chromatography [silica, hexanes to hexanes/CH_2_Cl_2_ (10:1)] to afford a white amorphous solid (204 mg, 23%): ^1^H NMR (600 MHz, CDCl_3_) *δ* 8.59 (t, *J* = 1.5 Hz, 1H), 8.33 (d, *J* = 1.5 Hz, 2H), 4.47–4.42 (m, 4H), 1.18–1.13 (m, 4H), 0.09 (s, 18H); ^13^C{^1^H} NMR (151 MHz, CDCl_3_) *δ* 166.2, 137.9, 134.3, 130.5, 123.9, 65.6, 18.9, 1.5; ESI-MS (negative-ion mode) obsd 442.92573, calcd 443.0715 [M – H]^–^, M = C_18_H_29_BrO_4_Si_2_. 

*Zinc(II) 10,20-Bis[2-(3,4-(N-(2,6-diisopropylphenyl)iminodicarbonyl)-1,6-bis(4-tert-butylphenoxy)perylen-9-yl)ethynyl]-5-(4-(2-(3,5-bis((2-(trimethylsilyl)ethoxy)carbonyl)phenyl)ethynyl)phenyl)-15-p-tolylporphyrin* (**27**). Following a standard procedure [56,57], a Schlenk flask containing zinc porphyrin triad **23** (51.9 mg, 24 µmol), bromoisophthalate **26** (16.0 mg, 36 µmol), and tri(*o*-tolyl)phosphine (11.0 mg, 36 µmol) was flushed with argon, treated with degassed toluene/TEA (12 mL, 5:1, *v*:*v*) and subjected to three freeze–pump–thaw cycles, and then treated with Pd_2_(dba)_3_ (6.6 mg, 7.2 µmol) and subjected to two additional freeze–pump–thaw cycles. The resulting mixture was heated to 60 °C for 5 h. The crude mixture was purified following the standard three-chromatography procedure [silica (hexanes/CH_2_Cl_2_ (2:1 to 1:1)), SEC (toluene), silica (hexanes to hexanes/CH_2_Cl_2_ (1:1))] to afford a dark red solid (42.0 mg, 69%): ^1^H NMR (600 MHz, CDCl_3_) *δ* 9.85–9.75 (m, 4H), 9.21–9.14 (m, 2H), 9.11 (d, *J* = 8.1 Hz, 2H), 8.99–8.94 (m, 2H), 8.91–8.87 (m, 2H), 8.67 (s, 2H), 8.51–8.43 (m, 3H), 8.30 (s, 4H), 8.16–8.11 (m, 2H), 8.10–8.06 (m, 2H), 8.03–7.99 (m, 2H), 7.98–7.94 (m, 2H), 7.61 (d, *J* = 7.4 Hz, 2H), 7.59–7.54 (m, 2H), 7.47–7.40 (m, 6H), 7.35–7.28 (m, 8H), 7.02 (d, *J* = 8.3 Hz, 4H), 6.69 (s, 4H), 4.53–4.46 (m, 4H), 2.82–2.72 (m, 7H), 1.34 (s, 18H), 1.31 (s, 18H), 1.22–1.12 (m, 28H), 0.14 (s, 18H); MALDI-MS (α-CHCA) obsd 2525.8, calcd 2525.0 (M^+^), M = C_165_H_148_N_6_O_12_Si_2_Zn; λ_abs_ (toluene) 441, 486, 516, 550, 713 nm, ε_549 nm_ = 99,800 M^−1^cm^−1^; λ_em_ (toluene, λ_exc_ = 549 nm) 733 nm.

*Zinc(II) 10,20-Bis[2-(3,4-(N-(2,6-diisopropylphenyl)iminodicarbonyl)-1,6-bis(4-tert-butylphenoxy)perylen-9-yl)ethynyl]-5-(4-(2-(3,5-dicarboxyphenyl)ethynyl)phenyl)-15-p-tolylporphyrin* (**28**). Following a reported method [76], a solution of **27** (30 mg, 12 µmol) in THF (30 mL) was treated with a tetrabutylammonium fluoride solution (50 µL, 1.0 M in THF) at room temperature for 2 h. The reaction mixture was diluted with CH_2_Cl_2_ (100 mL) and then washed with water (100 mL × 2) and brine (100 mL). The organic extract was dried, concentrated to dryness, and purified by chromatography [silica, CH_2_Cl_2_ to CH_2_Cl_2_/MeOH (5:1)] to afford a purple solid (16 mg, 59%): λ_abs_ (toluene) 454, 515, 756 nm, ε_454 nm_ = 44,400 M^−1^cm^−1^ (broad spectrum).

*10,20-Bis[2-(3,4-(N-(2,6-diisopropylphenyl)iminodicarbonyl)-1,6-bis(4-tert-butylphenoxy)perylen-9-yl)ethynyl]-5-(4-(2-(3,5-dimethoxycarbonylphenyl)ethynyl)phenyl)-15-p-tolylporphyrin* (**29**). A solution of **28** (2.0 mg, 0.86 µmol) and methanol (100 µL) in DMF (900 µL) was treated with DCC (1.0 mg, 4.8 µmol) and DMAP (1 mg, 8 µmol) at room temperature for 16 h. The reaction mixture was diluted with CH_2_Cl_2_ (20 mL) and then filtered. The filtrate was washed with water (20 mL) and a saturated NH_4_Cl aqueous solution (20 mL), dried, concentrated to dryness, and then dissolved in CH_2_Cl_2_ (500 µL). The resulting solution was used for MALDI-MS characterization without further purification: MALDI-MS (α-CHCA) obsd 2352.6, calcd 2352.9 (M^+^), M = C_157_H_128_N_6_O_12_Zn.

*Zinc(II) 5-(4-Ethynyl)phenyl-15-p-tolylporphyrin* (**30**). A solution of porphyrin **20** (63.6 mg, 0.10 mmol) in toluene (30 mL) and methanol (30 mL) was treated with K_2_CO_3_ (1.38 g, 10 mmol) at room temperature for 3 h. The reaction mixture was poured into water (120 mL) and extracted with CH_2_Cl_2_ (100 mL × 2). The combined organic extract was washed with brine (100 mL), dried, and then concentrated to dryness to afford a purple solid (51.1 mg, 91%): ^1^H NMR (600 MHz, CDCl_3_) *δ* 10.32 (s, 2H), 9.46–9.41 (m, 4H), 9.18 (d, *J* = 4.4 Hz, 2H), 9.11 (d, *J* = 4.4 Hz, 2H), 8.23 (d, *J* = 7.8 Hz, 2H), 8.15 (d, *J* = 7.4 Hz, 2H), 7.94 (d, *J* = 7.7 Hz, 2H), 7.61 (d, *J* = 7.4 Hz, 2H), 3.34 (s, 1H), 2.75 (s, 3H); MALDI-MS (α-CHCA) obsd 562.2, calcd 562.1 (M^+^), M = C_35_H_22_N_4_Zn; λ_abs_ (toluene) 414, 539, 576 nm. 

*Zinc(II) 5-(4-(2-(3,5-Bis((2-(trimethylsilyl)ethoxy)carbonyl)phenyl)ethynyl)phenyl)-15-p-tolylporphyrin* (**31**). Following a standard procedure [56,57], a Schlenk flask containing zinc porphyrin **30** (37.9 mg, 65 µmol), bromoisophthalate **26** (44.0 mg, 98 µmol), and tri(*o*-tolyl)phosphine (29.8 mg, 98 µmol) was flushed with argon, treated with degassed toluene/TEA (35 mL, 5:1, *v*:*v*), and subjected to three freeze–pump–thaw cycles, and then treated with Pd_2_(dba)_3_ (18.3 mg, 20 µmol) and subjected to two additional freeze–pump–thaw cycles. The resulting mixture was heated to 60 °C for 16 h. The crude mixture was concentrated to dryness and then purified by chromatography [silica, hexanes/CH_2_Cl_2_ (1:1) to CH_2_Cl_2_] to afford a purple solid (22.4 mg, 45%): ^1^H NMR (600 MHz, CDCl_3_) *δ* 10.34 (s, 2H), 9.47 (d, *J* = 4.4 Hz, 2H), 9.45 (d, *J* = 4.4 Hz, 2H), 9.19 (d, *J* = 4.4 Hz, 2H), 9.16 (d, *J* = 4.4 Hz, 2H), 8.69 (t, *J* = 1.6 Hz, 1H), 8.52 (d, *J* = 1.6 Hz, 2H), 8.29 (d, *J* = 7.9 Hz, 2H), 8.16 (d, *J* = 7.6 Hz, 2H), 8.00 (d, *J* = 7.9 Hz, 2H), 7.61 (d, *J* = 7.6 Hz, 2H), 4.55–4.48 (m, 4H), 2.75 (s, 3H), 1.25–1.20 (m, 4H), 0.14 (s, 18H); MALDI-MS (α-CHCA) obsd 926.6, calcd 926.3 (M^+^), M = C_53_H_50_N_4_O_4_Si_2_Zn; λ_abs_ (toluene) 415, 540, 575 nm; λ_em_ (λ_exc_ = 415 nm) 584, 633 nm. 

*Zinc(II) 5-(4-(2-(3,5-Dicarboxyphenyl)ethynyl)phenyl)-15-p-tolylporphyrin* (**32**). Following a reported method [76], a solution of porphyrin **31** (18.5 mg, 20 µmol ) in THF (6.0 mL) was treated with a tetrabutylammonium fluoride solution (200 µL, 1.0 M in THF) at room temperature for 2 h. The reaction mixture was diluted with CH_2_Cl_2_ (20 mL) and then washed with water (20 mL × 2) and brine (20 mL). The organic extract was dried, concentrated to dryness, and purified by chromatography [silica, CH_2_Cl_2_/MeOH (20:1 to 1:1) to afford a purple solid (9.8 mg, 67%): ^1^H NMR (600 MHz, dimethylsulfoxide-*d*_6_) δ 10.38 (s, 2H), 9.54 (d, *J* = 4.4 Hz, 2H), 9.50 (d, *J* = 4.3 Hz, 2H), 9.01 (d, *J* = 4.3 Hz, 2H), 8.97 (d, *J* = 4.4 Hz, 2H), 8.27 (d, *J* = 7.7 Hz, 2H), 8.23 (d, *J* = 7.4 Hz, 1H), 8.15–8.09 (m, 4H), 8.04 (d, *J* = 7.6 Hz, 2H), 7.66 (d, *J* = 7.5 Hz, 2H), 2.71 (s, 3H); ESI-MS (negative-ion mode) obsd 725.1157, calcd 725.1173 [M – H]^–^; ESI-MS obsd 727.1301, calcd 727.1318 [M + H]^+^, M = C_43_H_26_N_4_O_4_Zn; λ_abs_ (dimethylsulfoxide) 419, 548, 587 nm.

### 3.4. Fluorescence Yield Determinations

The Φ_f_ values were determined in the standard manner by a comparison of integrated spectra (corrected for instrument sensitivity) with a known fluorophore of similar absorption and fluorescence spectral features. For **13**, the standard was dyad **1** (Φ_f_ = 0.38) [42]. For **27**, the standard was triad **4** (Φ_f_ = 0.41) [42]. For **31**, the standard was *meso*-tetraphenylporphyrin (Φ_f_ = 0.070) [79]. The resulting Φ_f_ values for **13**, **27,** and **31** are 0.21, 0.24, and 0.011, respectively.

## 4. Outlook

Building block routes have been established for the preparation of triads comprised of two perylene-monoimides, one porphyrin, and a single tether. All of this work has emanated from the unexpected observation a decade ago that a perylene-ethynyl-porphyrin (Dyad **0**) exhibits an absorption spectrum essentially lacking a characteristic porphyrin Soret band [41], as shown in Figure 1 [45,46,47,49,50]. The panchromatic absorption provided by the perylene-ethynyl-porphyrin construct differs profoundly from that of the constituent parts. Among all other chromophore-tetrapyrrole constructs subsequently examined [42,45,46,47,48,49,50], including the exploration of the type and number of chromophores, the attachment site on the tetrapyrrole, and the composition of the tetrapyrrole, the linear (i.e., *trans*) arrangement of perylene-ethynyl-porphyrin-ethynyl-perylene with attachment at the porphyrin meso-positions has proven superior for panchromaticity and photophysical features. The triads described herein provide absorption across the 350–750 nm region and fluorescence in the near-infrared region. Such spectral features closely resemble those of triads prepared previously that lack tethers. The triads described herein are hydrophobic and may be best suited for use in membraneous assemblies and other lipophilic environments. For perspective, a phenazinyl-ethynyl-porphyrin that bears a benzoic acid tether has been prepared [80]. Triads **13** and **27** provide broader spectral coverage but also are substantially larger. The building block chemistry described herein should enable the preparation of a variety of porphyrin constructs with a range of tethers for studies of panchromatic absorbers in diverse applications.

## Data Availability

All data are contained within the paper and Supplementary Materials.

## Supplementary Materials

The following supporting information can be downloaded at: https://www.mdpi.com/article/10.3390/molecules27196501/s1, ^1^H and ^13^C{^1^H} NMR spectra, mass spectra, and absorption spectra (where available) for new compounds, comprising 59 pages.

## Sample Availability

Samples have generally been consumed during the course of research.
