# Supplementary material for: Panchromatic Absorbers Tethered for Bioconjugation or Surface Attachment
Source: Molecules. 2022 Oct 1;27(19):6501. doi: 10.3390/molecules27196501 (PMC9573448; doi:10.3390/molecules27196501)
Supplement: Supplementary file 1 [file molecules-27-06501-s001.zip › molecules-1930350-supplementary.pdf]

Supplementary Materials for:

Panchromatic Absorbers Tethered for Bioconjugation or Surface Attachment

Rui Liu,[1,a] Jie Rong,[1,a] Zhiyuan Wu,[1,a] Masahiko Taniguchi,[1]
David F. Bocian,[2,*] Dewey Holten,[3,*] and Jonathan S. Lindsey[1,*]

[1] Department of Chemistry
North Carolina State University
Raleigh, North Carolina 27695-8204

[2] Department of Chemistry
University of California
Riverside, California 92521-0403

[3] Department of Chemistry
Washington University
St. Louis, Missouri 63130-4889

[a] Equal contributions

^{1}H NMR spectra, ^{13}C{^{1}H} spectra, MALDI-MS spectra, ESI-MS spectra, and absorption spectra (where available) for new compounds are provided in the following pages.

7: ^{1}H NMR, THF-d_8, 400 MHz

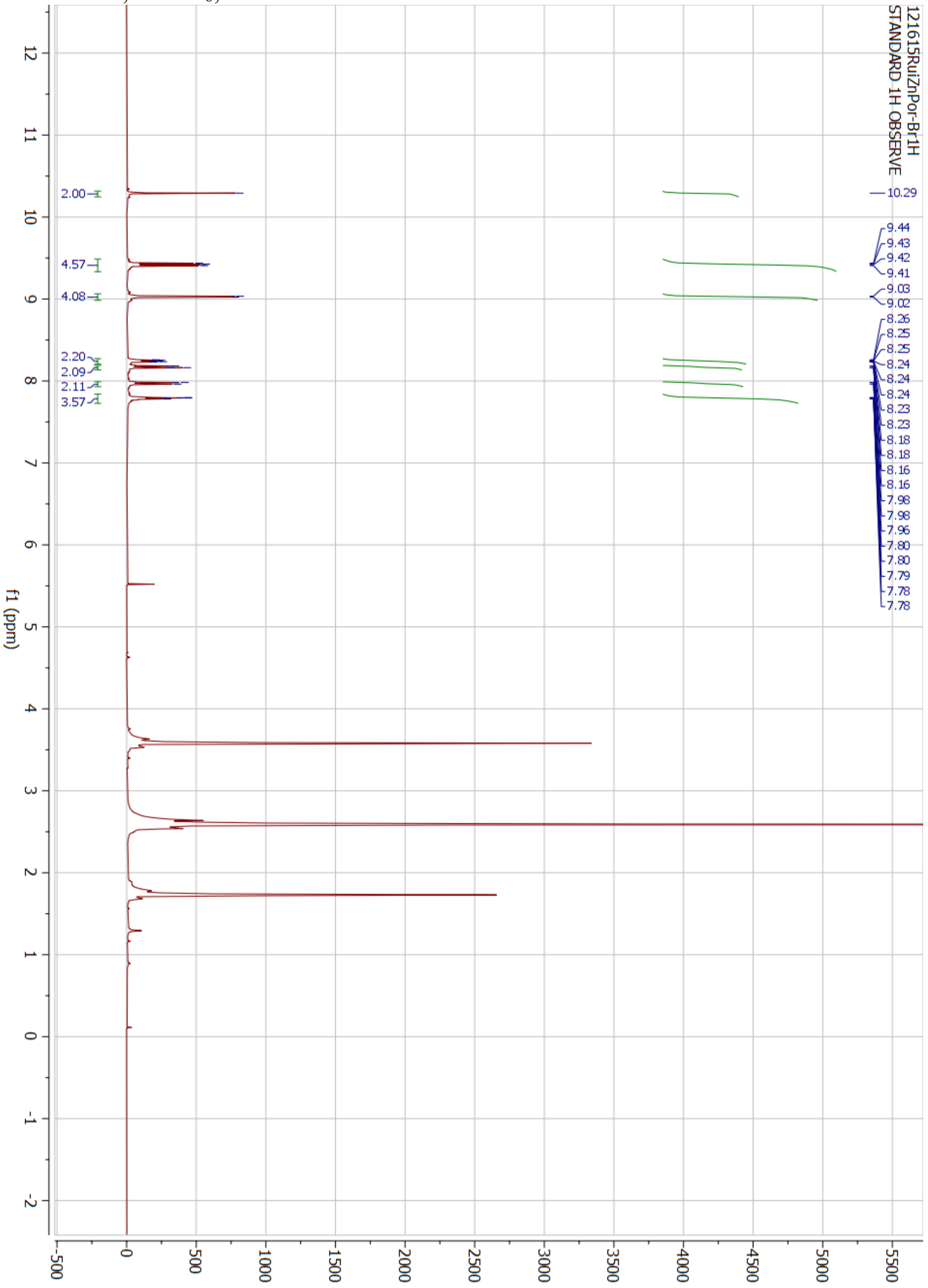

7: ^{13}C NMR, THF-d_8, 100 MHz

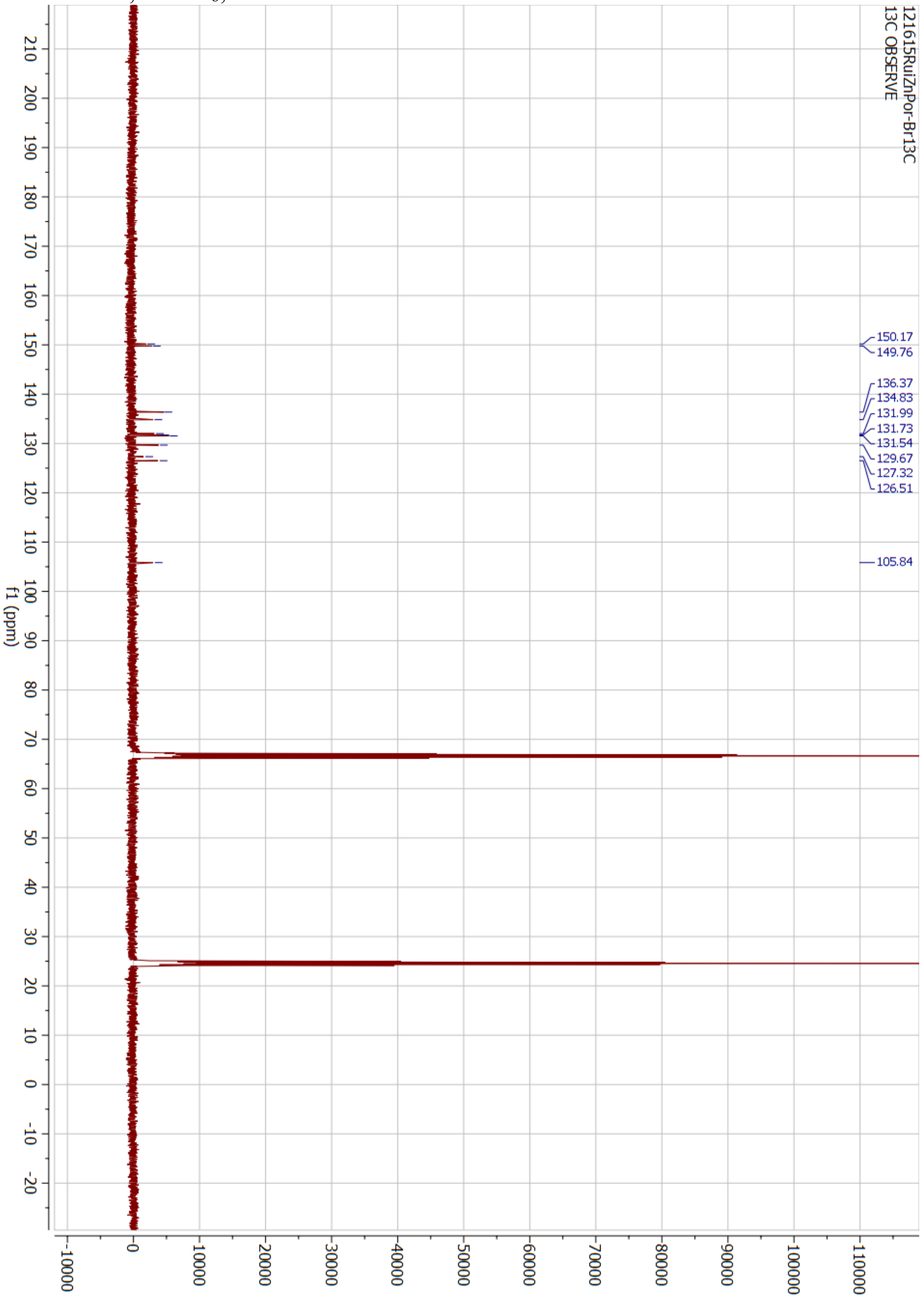

7: MALDI-MS

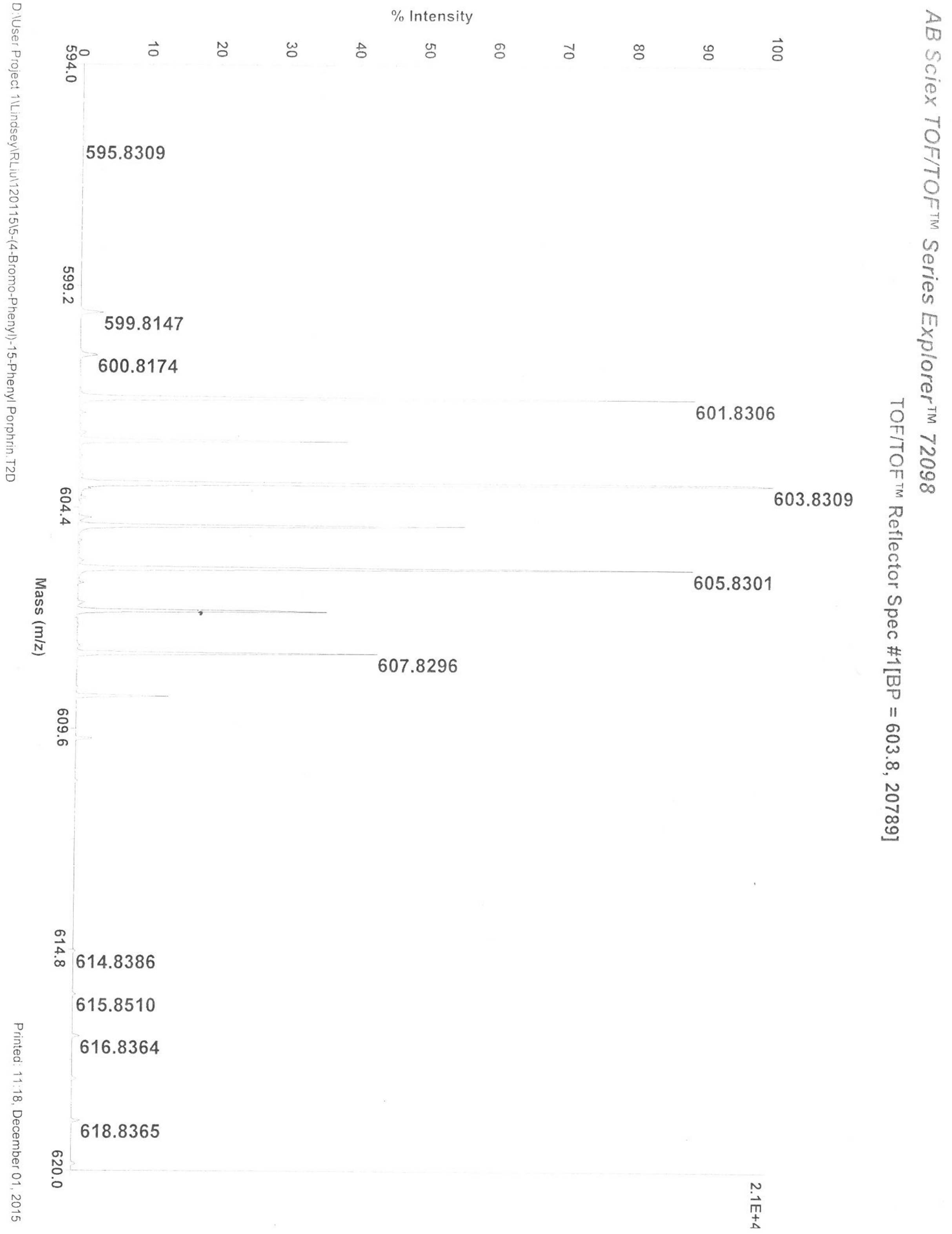
AB Sciex TOF/TOF™ Series Explorer™ 72098
TOF/TOF™ Reflector Spec #1[BP = 603.8, 20789]
% Intensity
0
10
20
30
40
50
60
70
80
90
100
595.8309
599.8147
600.8174
601.8306
603.8309
605.8301
607.8296
614.8386
615.8510
616.8364
618.8365
594.0
599.2
604.4
609.6
614.8
620.0
Mass (m/z)
2.1E+4
D:\User Project 1\Lindsey\RLiu\12011515-(4-Bromo-Phenyl)-15-Phenyl Porphrin.T2D
Printed: 11:18, December 01, 2015

7: ESI-MS

NC STATE UNIVERSITY
Mass Spectrometry Facility
Department of Chemistry
P.O. Box 8204
Raleigh, NC 27695-8204
http://www.ncsu.edu/chemistry/msf

120815 RL04 Full Scan

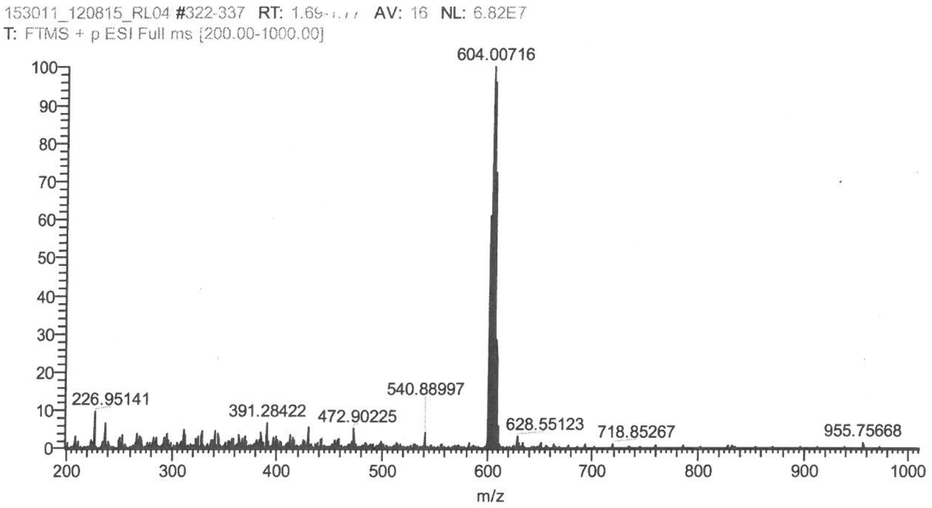

120815 RL04 Experimetnal and Theoretical Isotopic Distribution for $C_{32}H_{19}BrN_4Zn$ [M]⁺

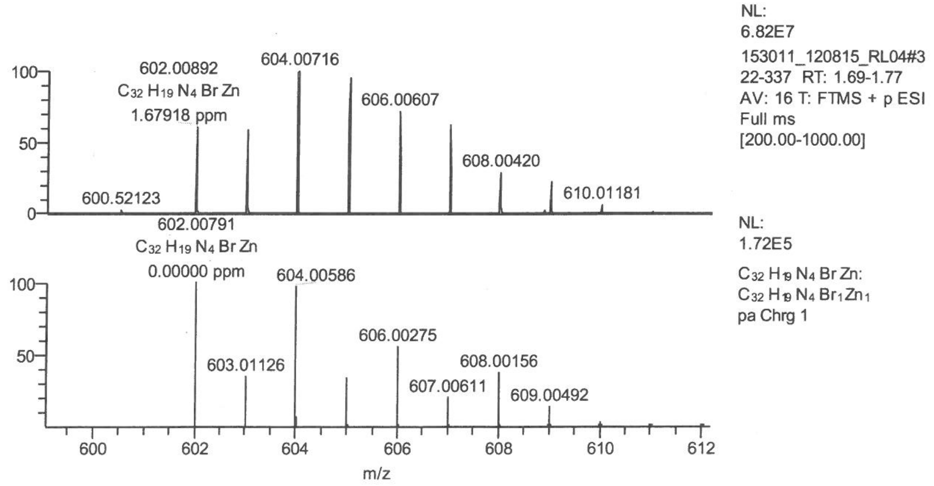

7: Absorption spectrum (toluene)

Spectrum/Peak Report Date 6/9/2016 Time 13:04:52 Page 1 of 1

Method file : <untitled>
Information : Default Method
Data File : C:\Chem32\1\DATA\Rui\060916ZNPORBR.SD Created : 6/9/16 12:46:53

Overlaid Spectra:

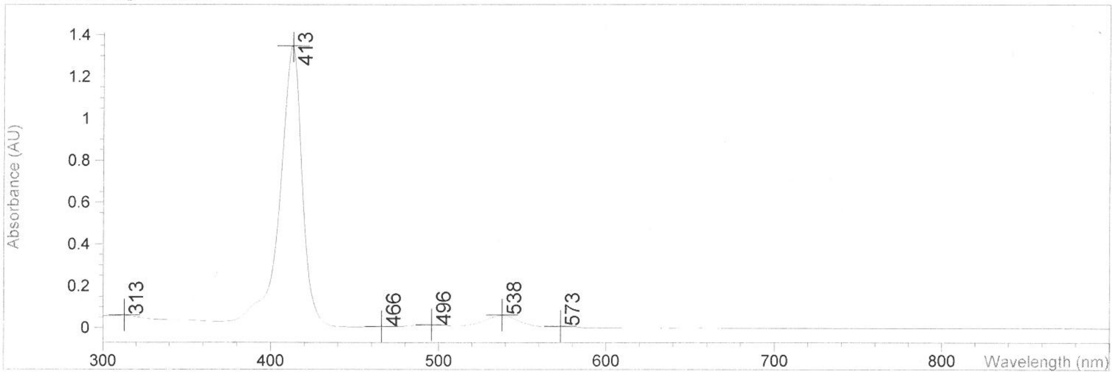

#	Name	Peaks (nm)	Abs (AU)
1		413.0	1.34620
1		538.0	6.1114E-2
1		313.0	5.9897E-2
1		496.0	1.4721E-2
1		573.0	7.0415E-3
1		466.0	6.7382E-3

Report generated by : Lindsey Lab Signature: ....

*** End Spectrum/Peak Report ***

9: ^{1}H NMR, THF-d_8, 400 MHz

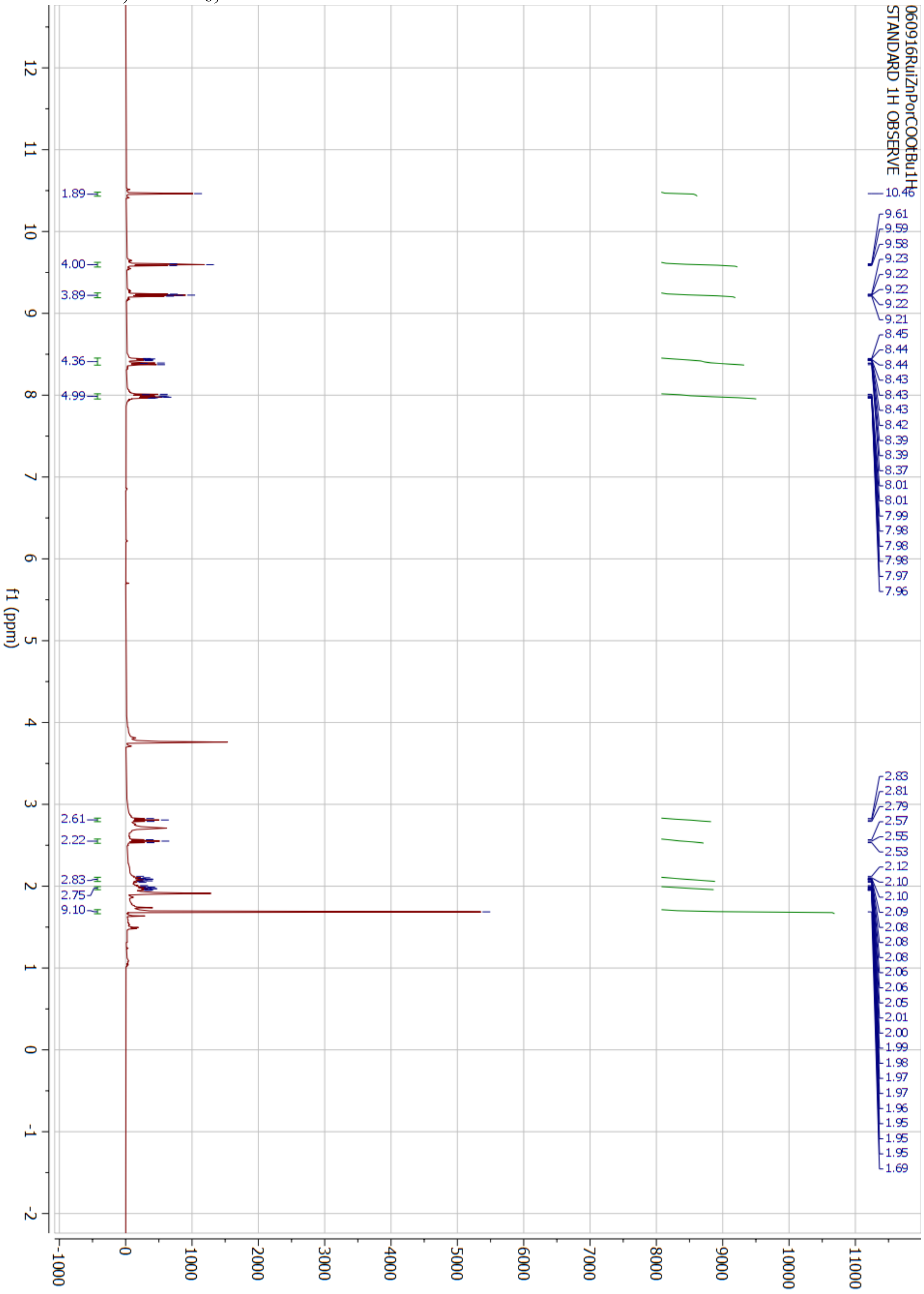

9: ^{13}C NMR, THF-d_8, 100 MHz

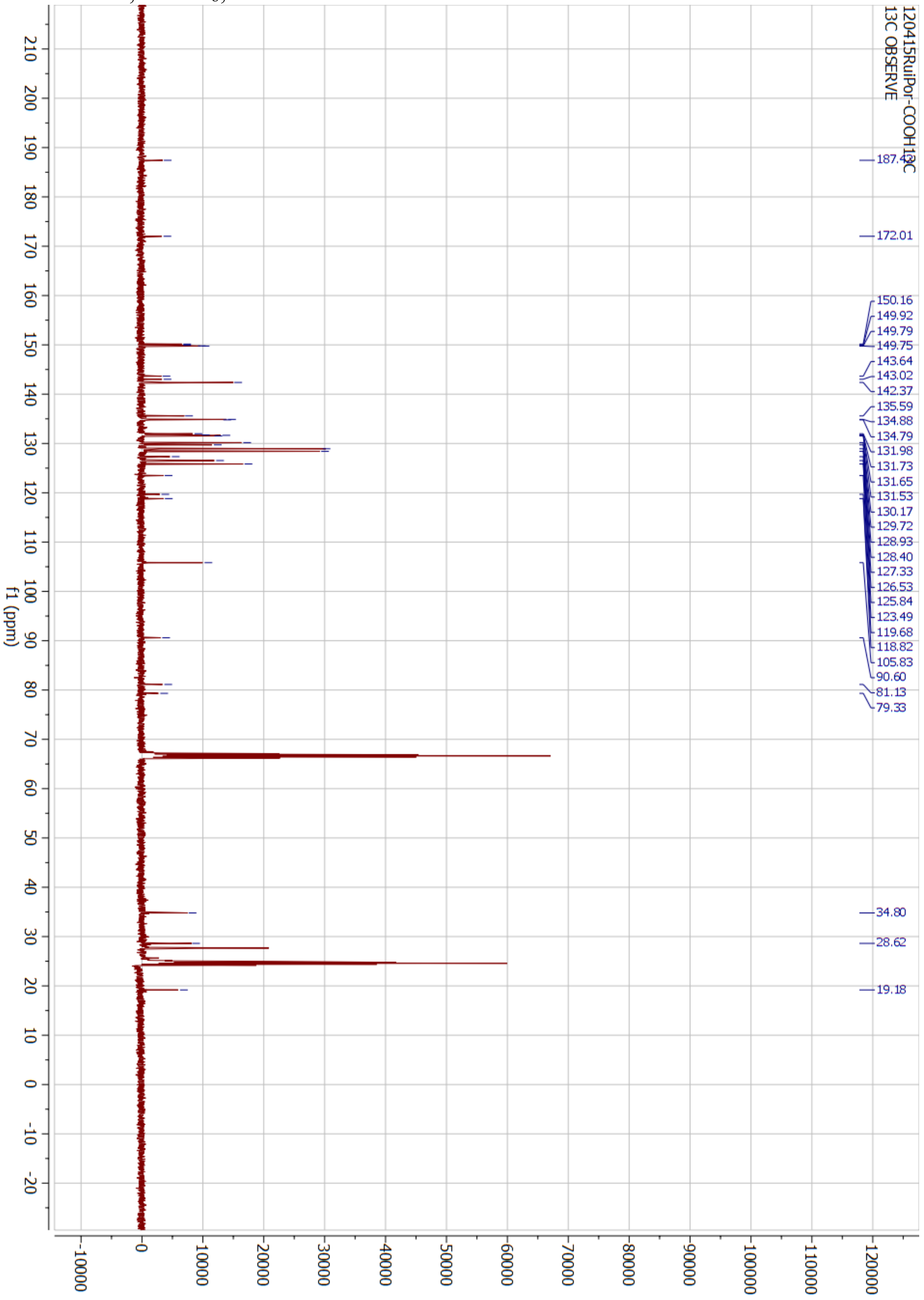

9: ESI-MS

NC STATE UNIVERSITY
Mass Spectrometry Facility
Department of Chemistry
P.O. Box 8204
Raleigh, NC 27695-8204
http://www.ncsu.edu/chemistry/msf

120815 RL03 Full Scan

153010_120815_RL03 #558-573 RT: 2.93-3.[illegible] 16 NL: 3.71E7
T: FTMS + p ESI Full ms [200.00-1000.00]

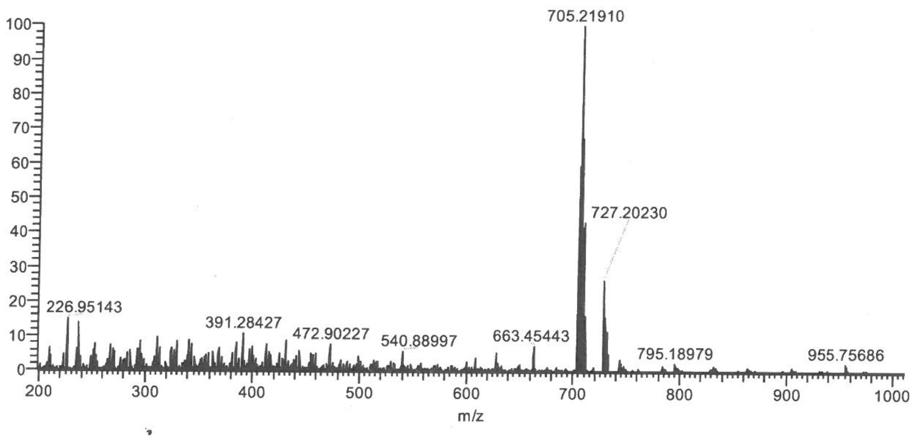

120815 RL03 Experimetnal and Theoretical Isotopic Distribution for $C_{43}H_{36}N_4O_2Zn$ $[M]^+$

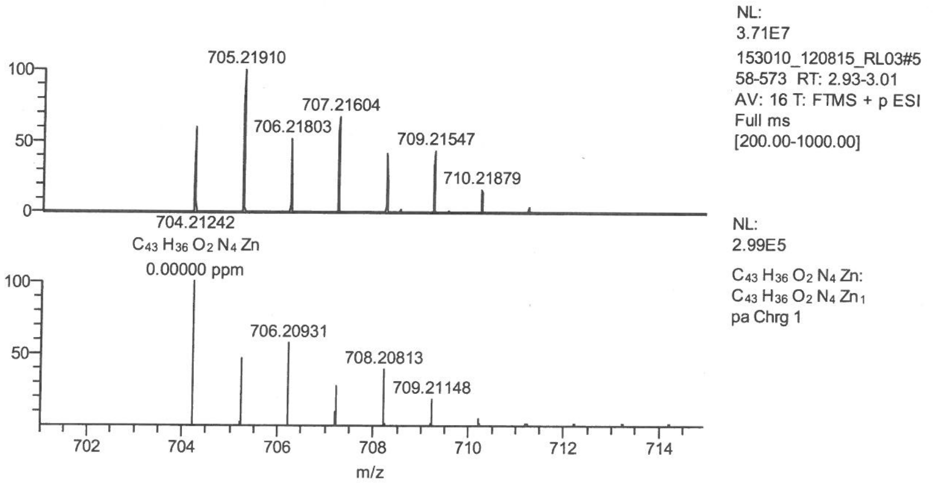

9: Absorption spectrum (toluene)

Spectrum/Peak Report   Date 6/9/2016 Time 13:05:15   Page 1 of 1

Method file : <untitled>
Information : Default Method
Data File : C:\Chem32\1\DATA\Rui\060916ZNPORCOOTBU.SD   Created : 6/9/16 12:50:34

Overlaid Spectra:

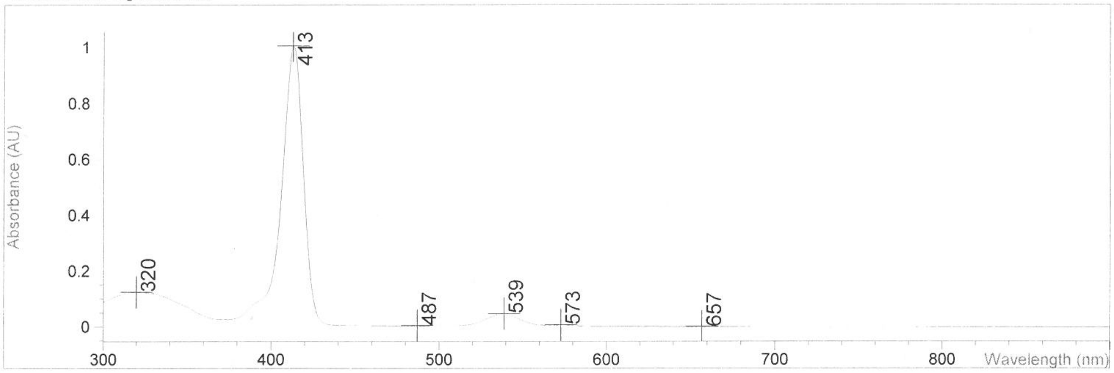

#	Name	Peaks (nm)	Abs (AU)
1		413.0	1.00640
1		320.0	0.12468
1		539.0	4.7800E-2
1		573.0	7.1979E-3
1		487.0	4.3850E-3
1		657.0	1.4977E-3

Report generated by : Lindsey Lab   Signature: ....

*** End Spectrum/Peak Report ***

10: ^{1}H NMR, THF-d_8, 400 MHz

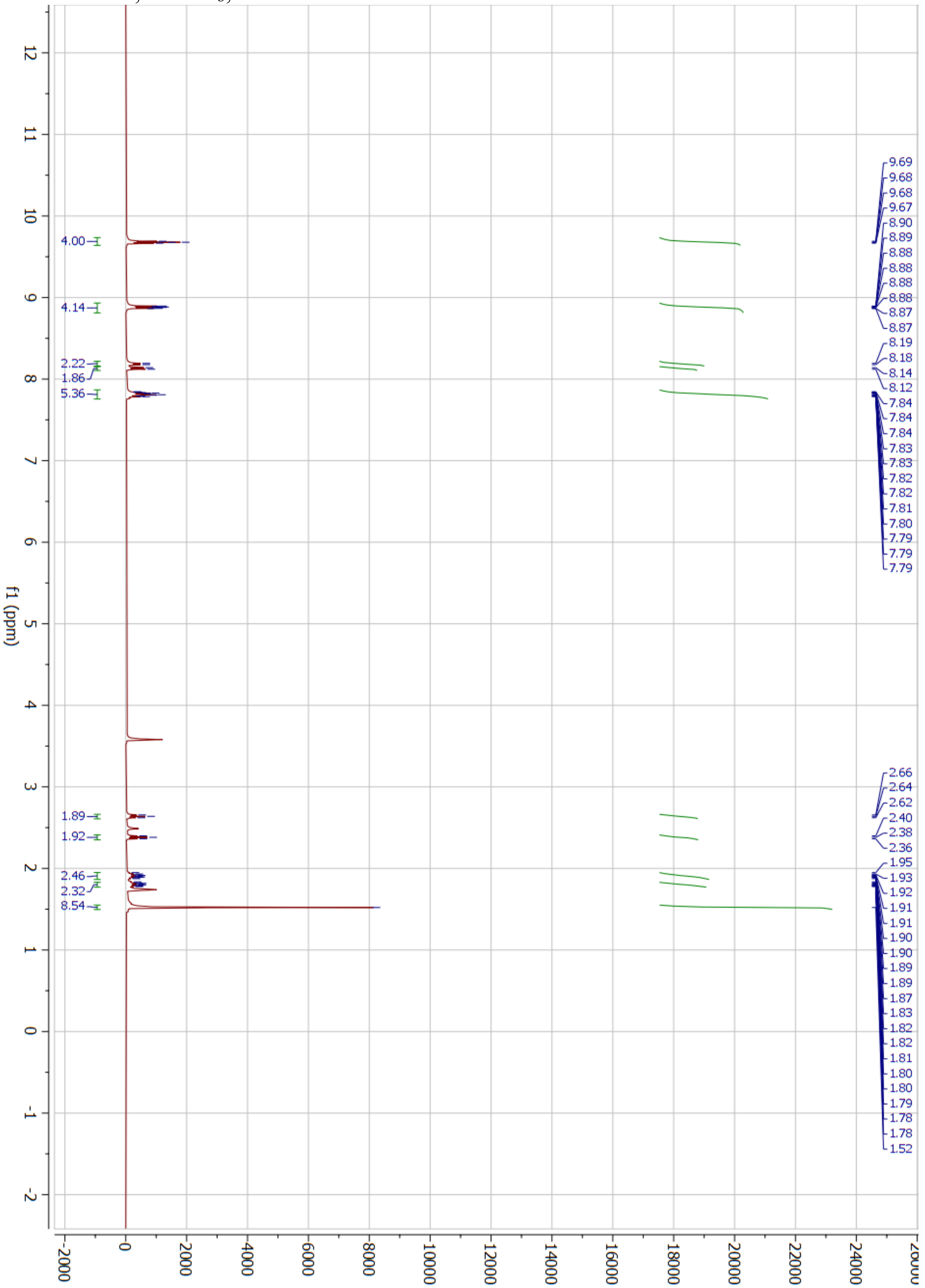

10: ^{13}C NMR, THF-d_8, 100 MHz

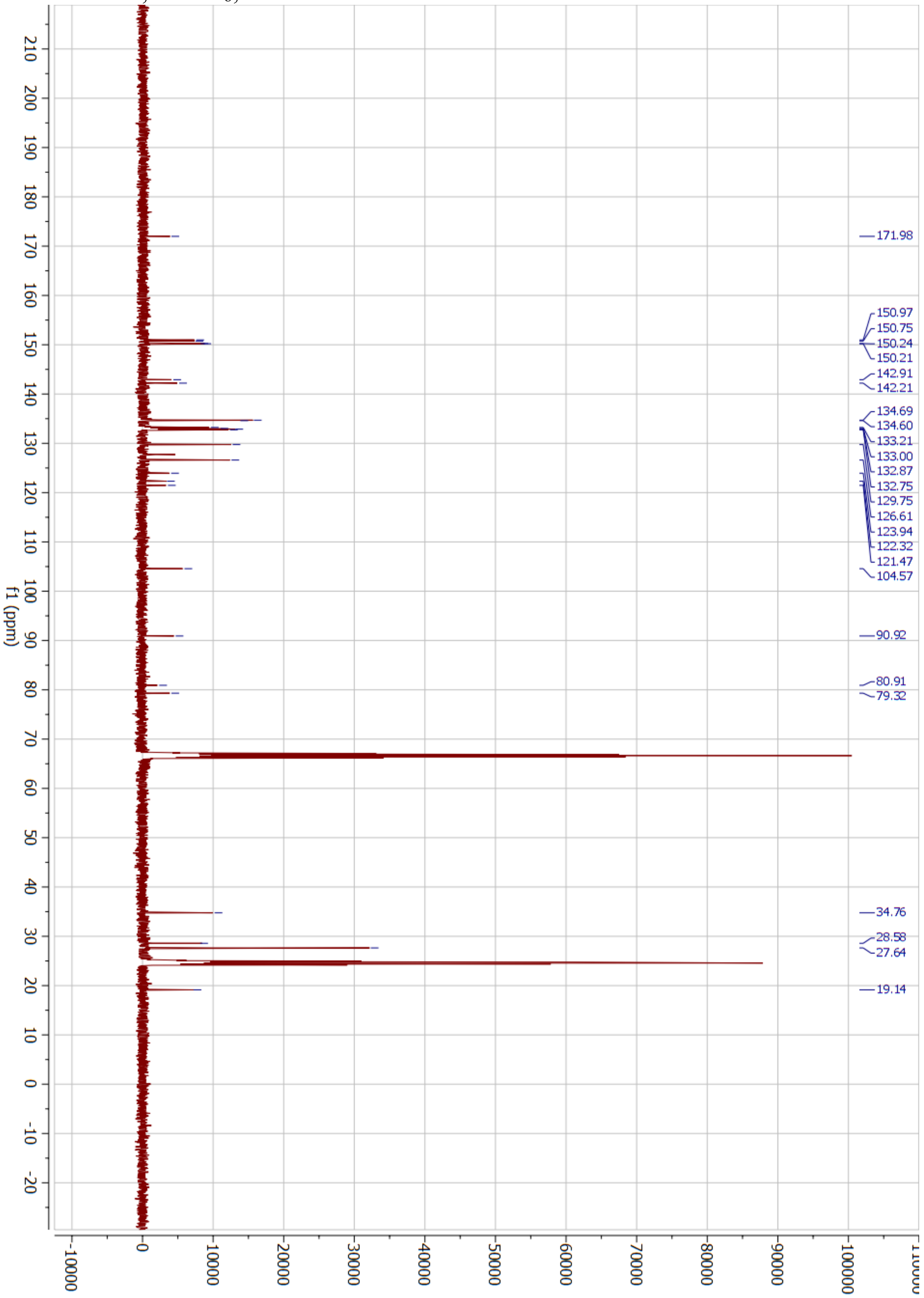

10: ESI-MS

NC STATE UNIVERSITY
Mass Spectrometry Facility
Department of Chemistry
P.O. Box 8204
Raleigh, NC 27695-8204
http://www.ncsu.edu/chemistry/msf

120815 RL02 Full Scan

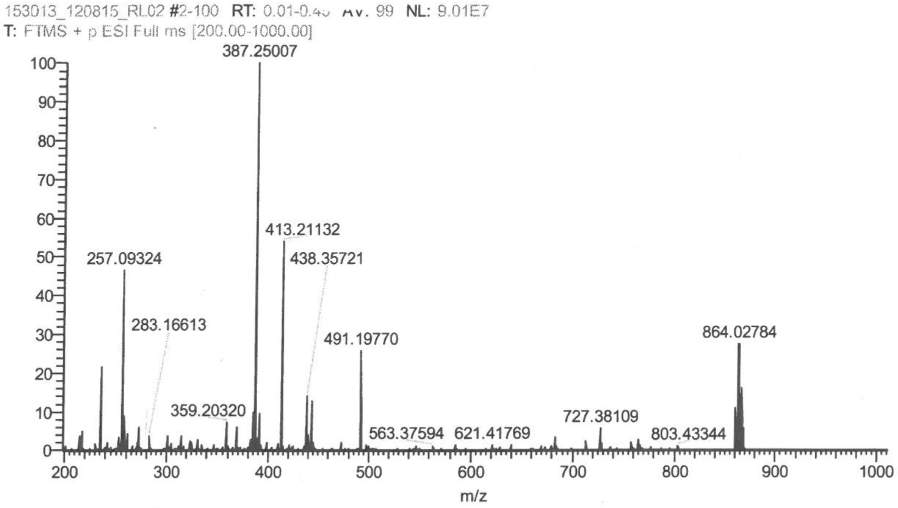

120815 RL02 Experimetnal and Theoretical Isotopic Distribution for $C_{43}H_{34}Br_2N_4O_2Zn$ [M]⁺

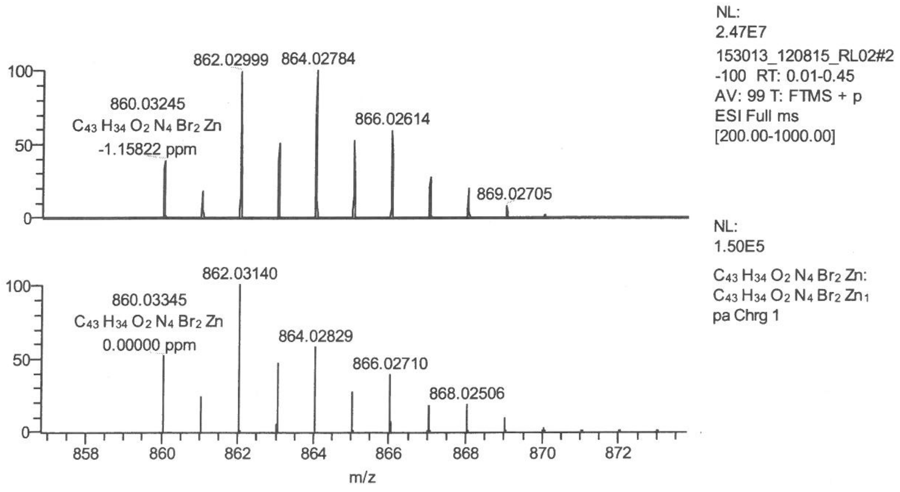

Page **4** of **7**

10: Absorption spectrum (toluene)

```
================================================================================
Spectrum/Peak Report                        Date 6/9/2016 Time 13:04:32   Page   1 of 1
================================================================================

Method file :         <untitled>
Information :         Default Method
Data File   :         C:\CHEM32\1\DATA\RUI\060916ZNPORBR2COOTBU.SD     Created : 6/9/16
                      13:03:26

Overlaid Spectra:
```

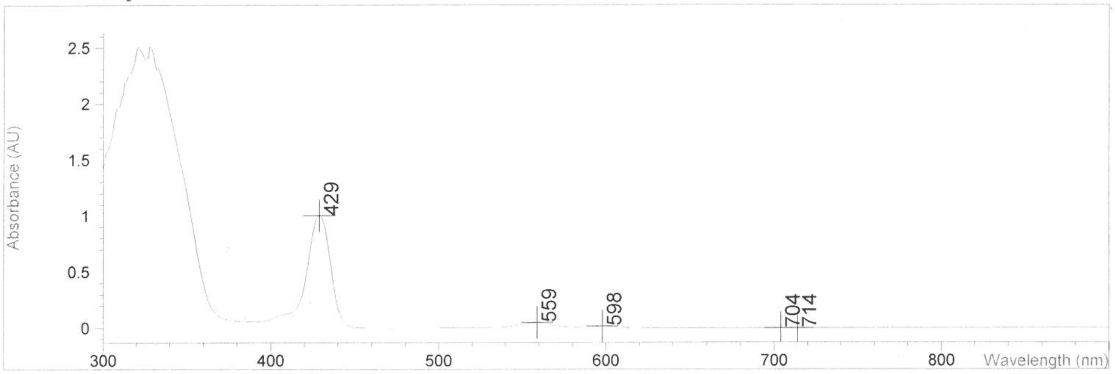


```
 #  Name                 Peaks(nm)      Abs(AU)
-------------------------------------------------
 1                           429.0      1.00520
 1                           559.0   4.8405E-2
 1                           598.0   1.7118E-2
 1                           714.0  -9.1267E-4
 1                           704.0  -9.2983E-4
 1                     000000000.0 -1.7977E308

Report generated by : Lindsey Lab                          Signature: ................

--------------------------------------------------------------------------------
                       *** End Spectrum/Peak Report ***
--------------------------------------------------------------------------------
```

12: ^{1}H NMR, $CDCl_3$, 300 MHz

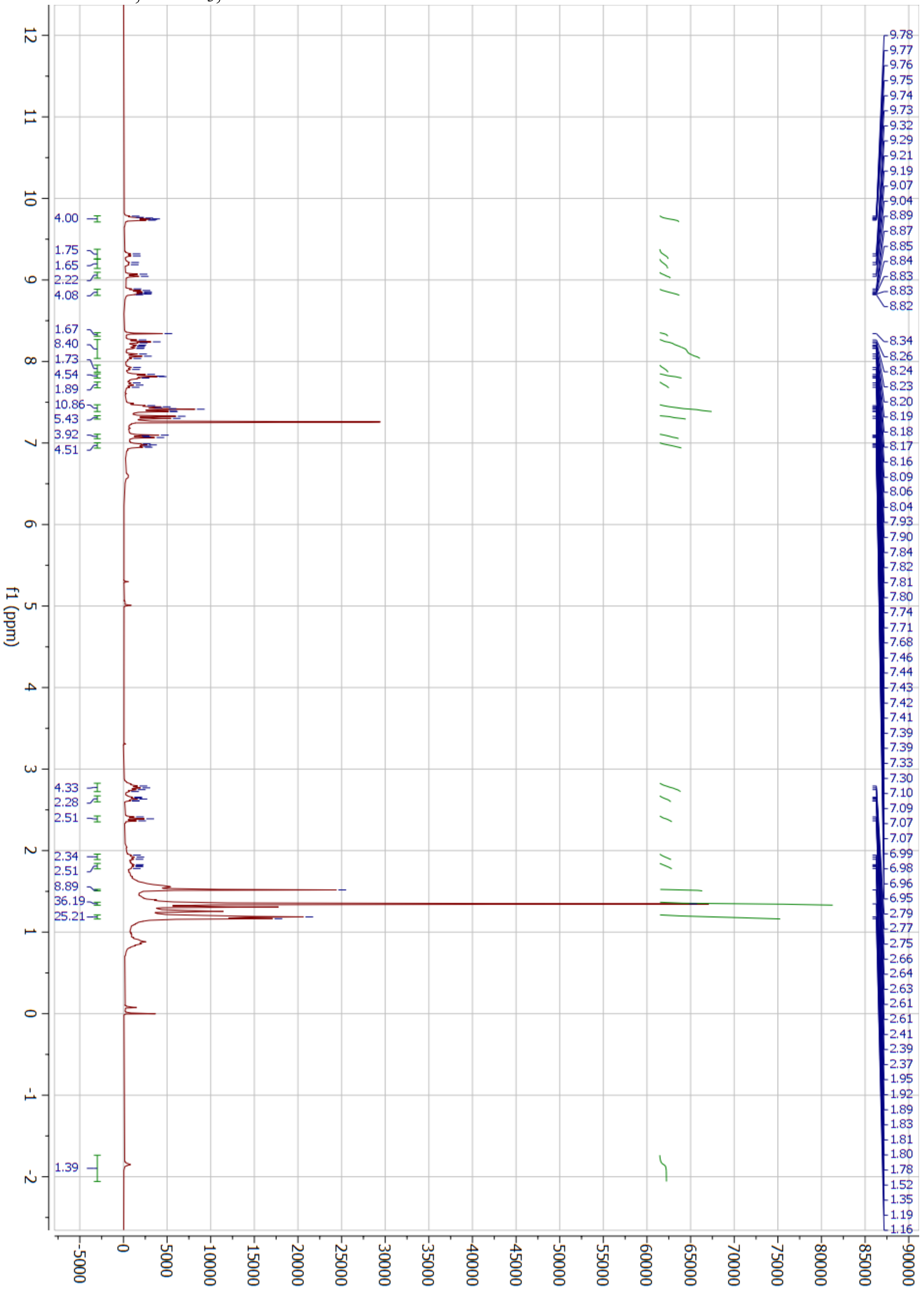

12: MALDI-MS

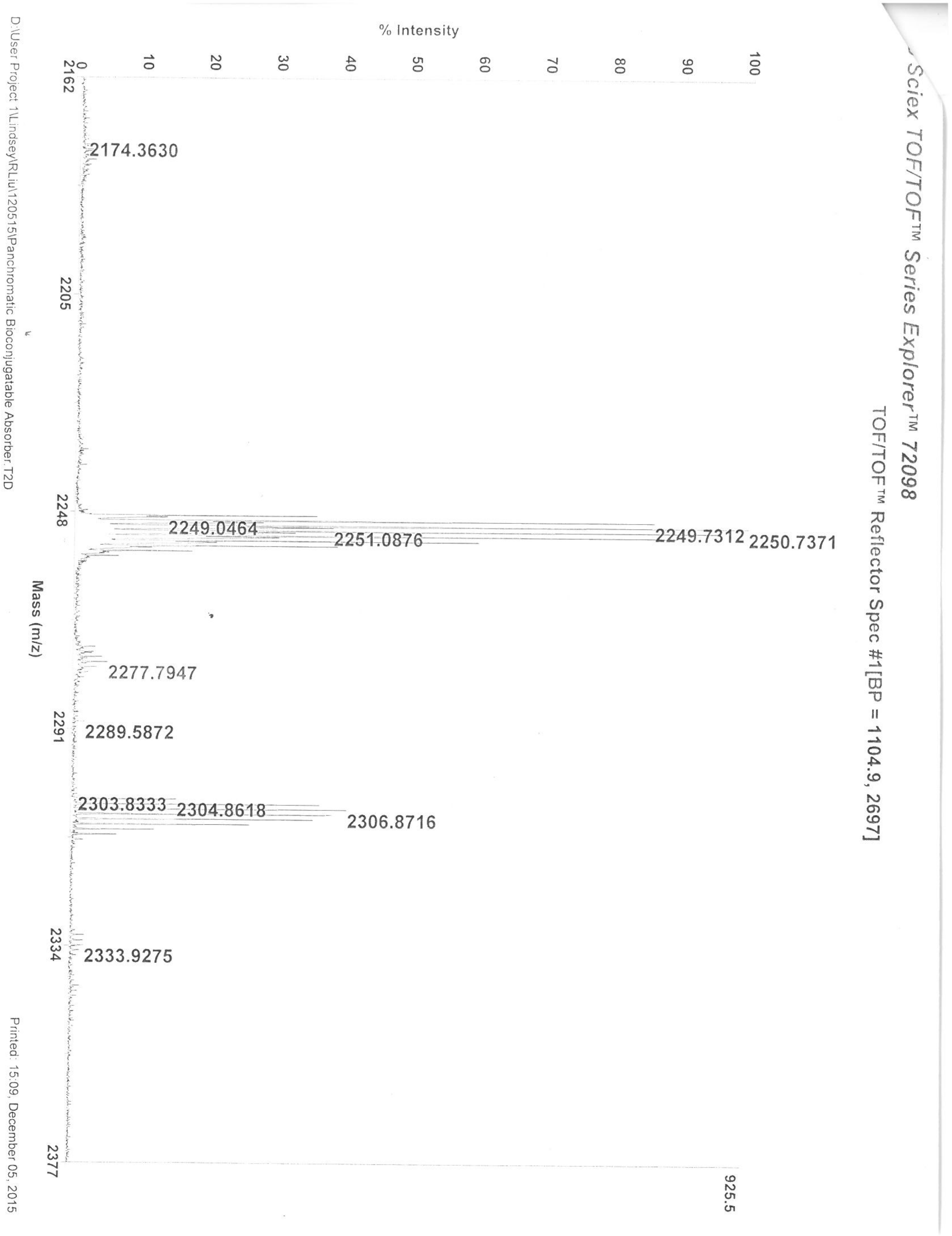

Sciex TOF/TOF™ Series Explorer™ 72098
TOF/TOF™ Reflector Spec #1[BP = 1104.9, 2697]
% Intensity
0
10
20
30
40
50
60
70
80
90
100
2174.3630
2249.0464
2251.0876
2249.7312
2250.7371
2277.7947
2289.5872
2303.8333
2304.8618
2306.8716
2333.9275
2162
2205
2248
2291
2334
2377
Mass (m/z)
925.5
D:\User Project 1\Lindsey\RLiu\120515\Panchromatic Bioconjugatable Absorber.T2D
Printed: 15:09, December 05, 2015

12: Absorption spectrum (toluene)

Spectrum/Peak Report Date 6/7/2016 Time 14:13:07 Page 1 of 1

Method file : <untitled>
Information : Default Method
Data File : C:\Chem32\1\DATA\Rui\032516 RPER2T-POR-COOTBU.SD Created : 3/25/16 13:30:44

Overlaid Spectra:

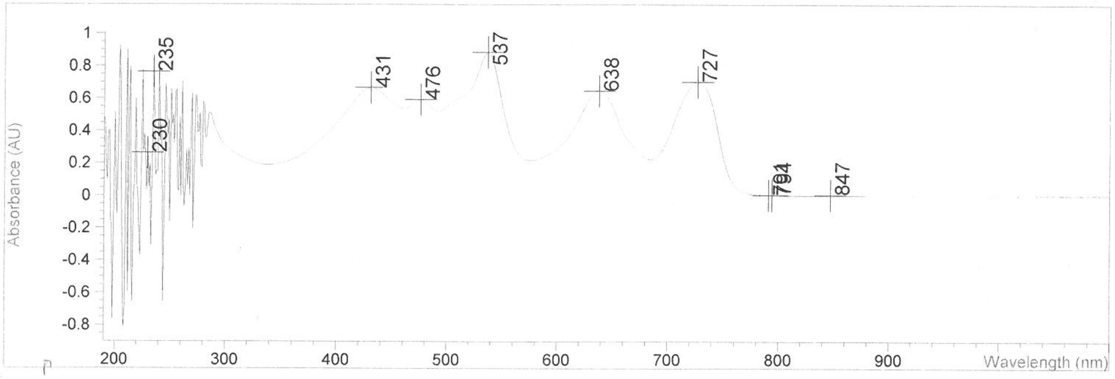

#	Name	Peaks(nm)	Abs(AU)
1		537.0	0.88411
1		235.0	0.76264
1		727.0	0.70325
1		431.0	0.66651
1		638.0	0.64723
1		476.0	0.59303
1		230.0	0.26232
1		794.0	5.4092E-3
1		791.0	5.2538E-3
1		847.0	4.7388E-3

Report generated by : Lindsey Lab Signature: ....

*** End Spectrum/Peak Report ***

13: ^{1}H NMR, $CDCl_3$, 300 MHz

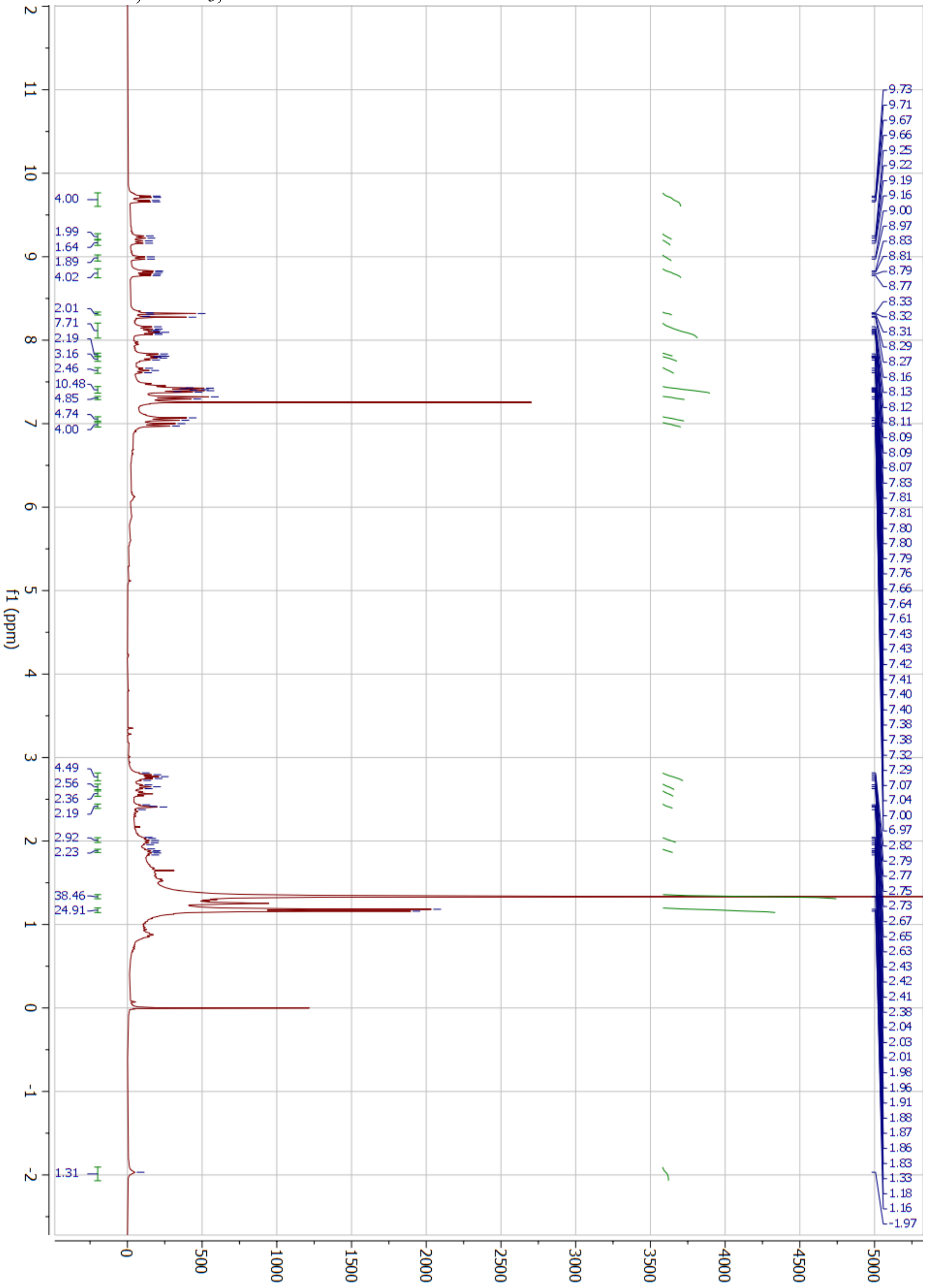

13: MALDI-MS

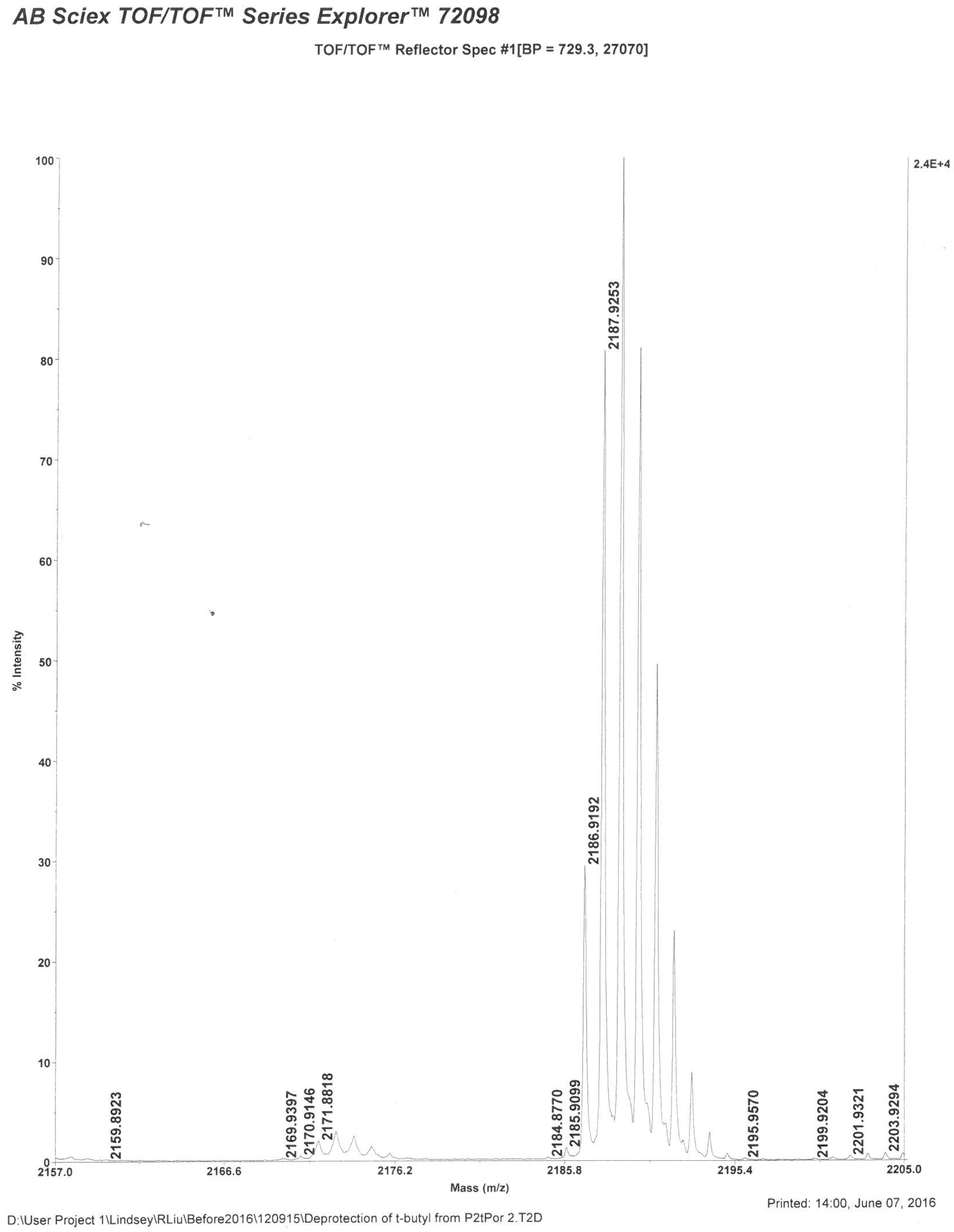

AB Sciex TOF/TOF™ Series Explorer™ 72098
TOF/TOF™ Reflector Spec #1[BP = 729.3, 27070]
2.4E+4
% Intensity
Mass (m/z)
2187.9253
2186.9192
2159.8923
2169.9397
2170.9146
2171.8818
2184.8770
2185.9099
2195.9570
2199.9204
2201.9321
2203.9294
2157.0
2166.6
2176.2
2185.8
2195.4
2205.0
Printed: 14:00, June 07, 2016
D:\User Project 1\Lindsey\RLiu\Before2016\120915\Deprotection of t-butyl from P2tPor 2.T2D

13: Absorption spectrum (toluene)

```
================================================================================
Spectrum/Peak Report                     Date 6/11/2016 Time 13:41:39  Page   1 of 1
================================================================================

Method file :          <untitled>
Information :          Default Method
Data File   :          C:\Chem32\1\DATA\Rui\120915 P2TPOR-COOH IN TOLUENE.SD     Created :
                       12/9/15 14:22:40

Overlaid Spectra:
```

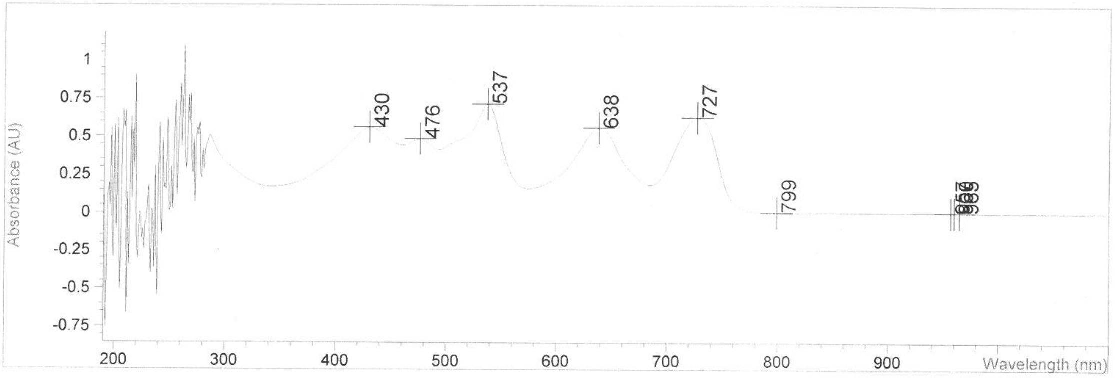


```
#  Name                 Peaks(nm)     Abs(AU)
---------------------------------------------
1                           537.0     0.71428
1                           727.0     0.62812
1                           430.0     0.56336
1                           638.0     0.55953
1                           476.0     0.48743
1                           799.0   6.5522E-3
1                           960.0   5.3821E-3
1                           957.0   5.3663E-3
1                           965.0   5.2128E-3
1                     000000000.0 -1.7977E308

Report generated by : Lindsey Lab                       Signature: ................
--------------------------------------------------------------------------------
                     *** End Spectrum/Peak Report ***
--------------------------------------------------------------------------------
```

14: ^{1}H NMR, $CDCl_3$, 300 MHz

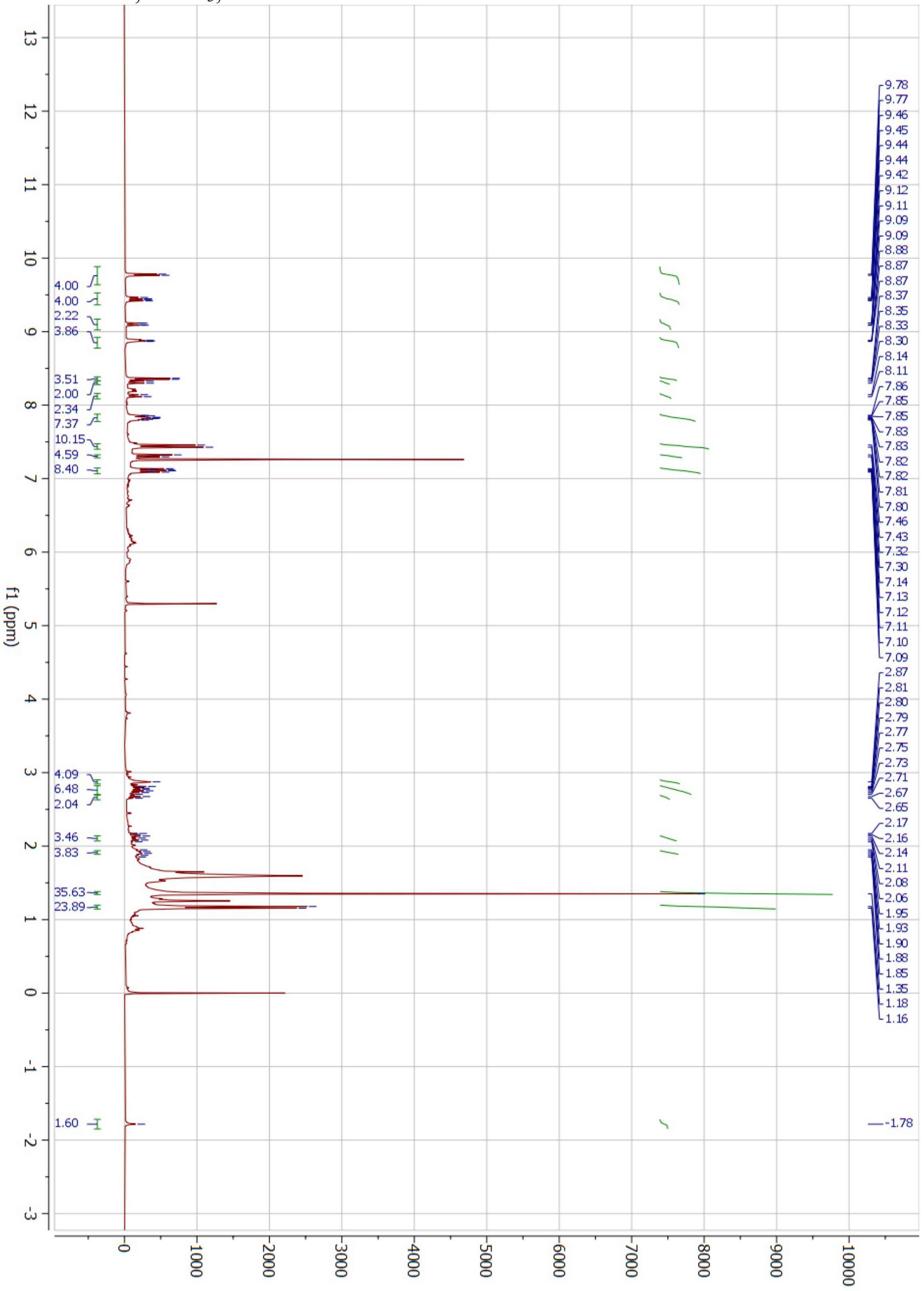

14: MALDI-MS

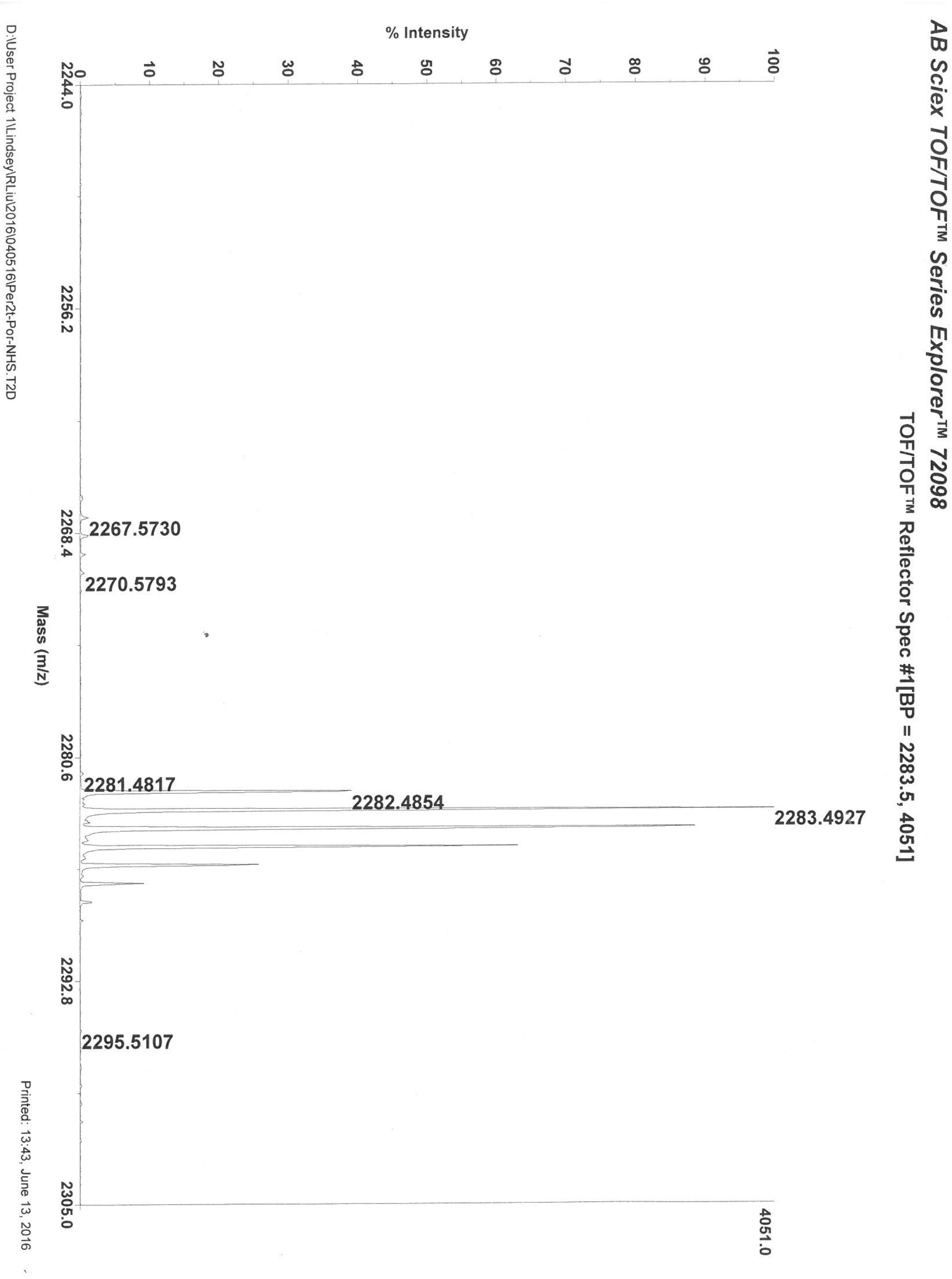

AB Sciex TOF/TOF™ Series Explorer™ 72098
TOF/TOF™ Reflector Spec #1[BP = 2283.5, 4051]
% Intensity
100
90
80
70
60
50
40
30
20
10
0
2244.0
2256.2
2268.4
2280.6
2292.8
2305.0
4051.0
Mass (m/z)
2267.5730
2270.5793
2281.4817
2282.4854
2283.4927
2295.5107
D:\User Project 1\Lindsey\RLiu\20160405\16\Per2t-Por-NHS.T2D
Printed: 13:43, June 13, 2016

20: ^{1}H NMR, $CDCl_3$, 600 MHz

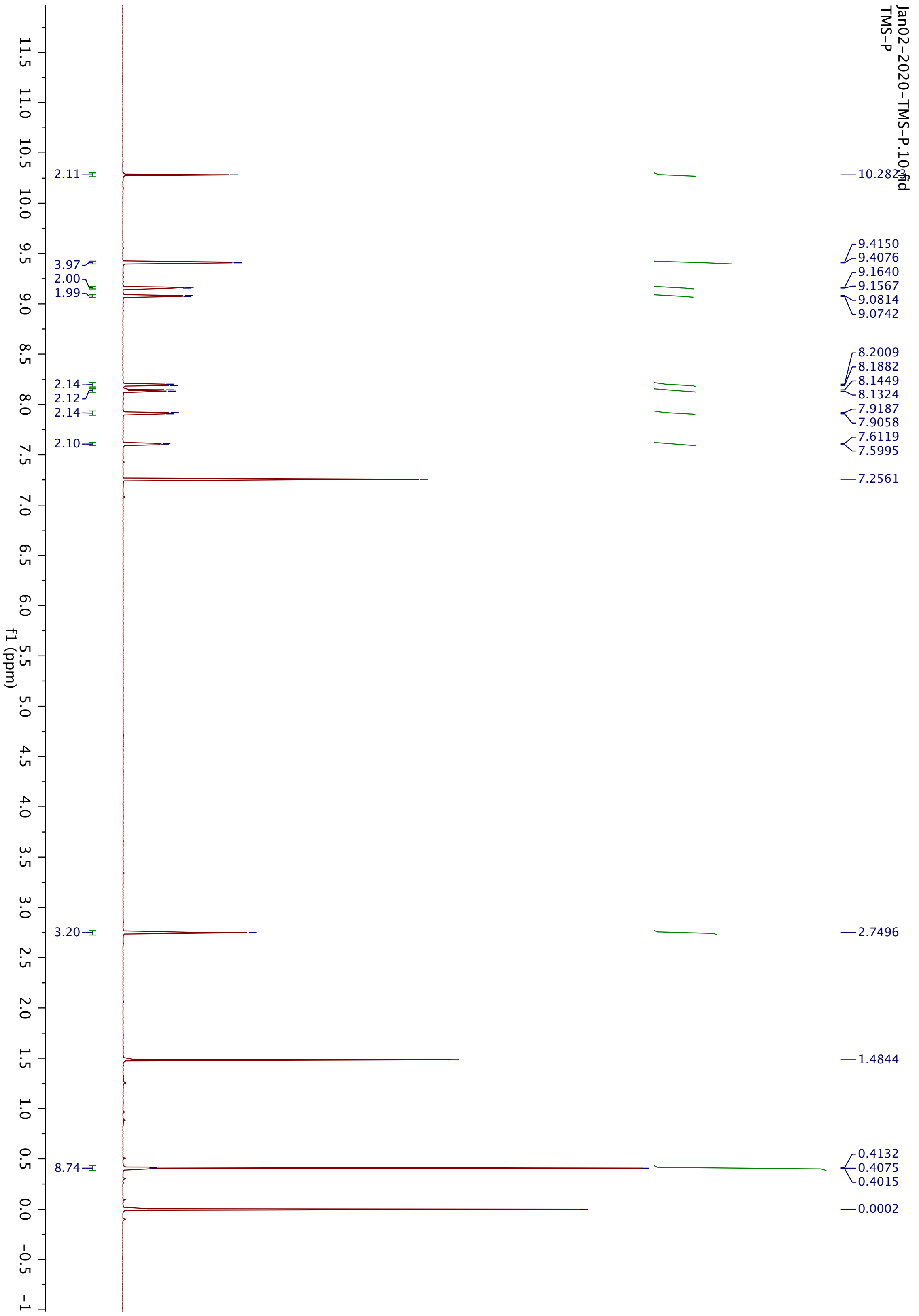

20: ^{13}C NMR, $CDCl_3$, 151 MHz

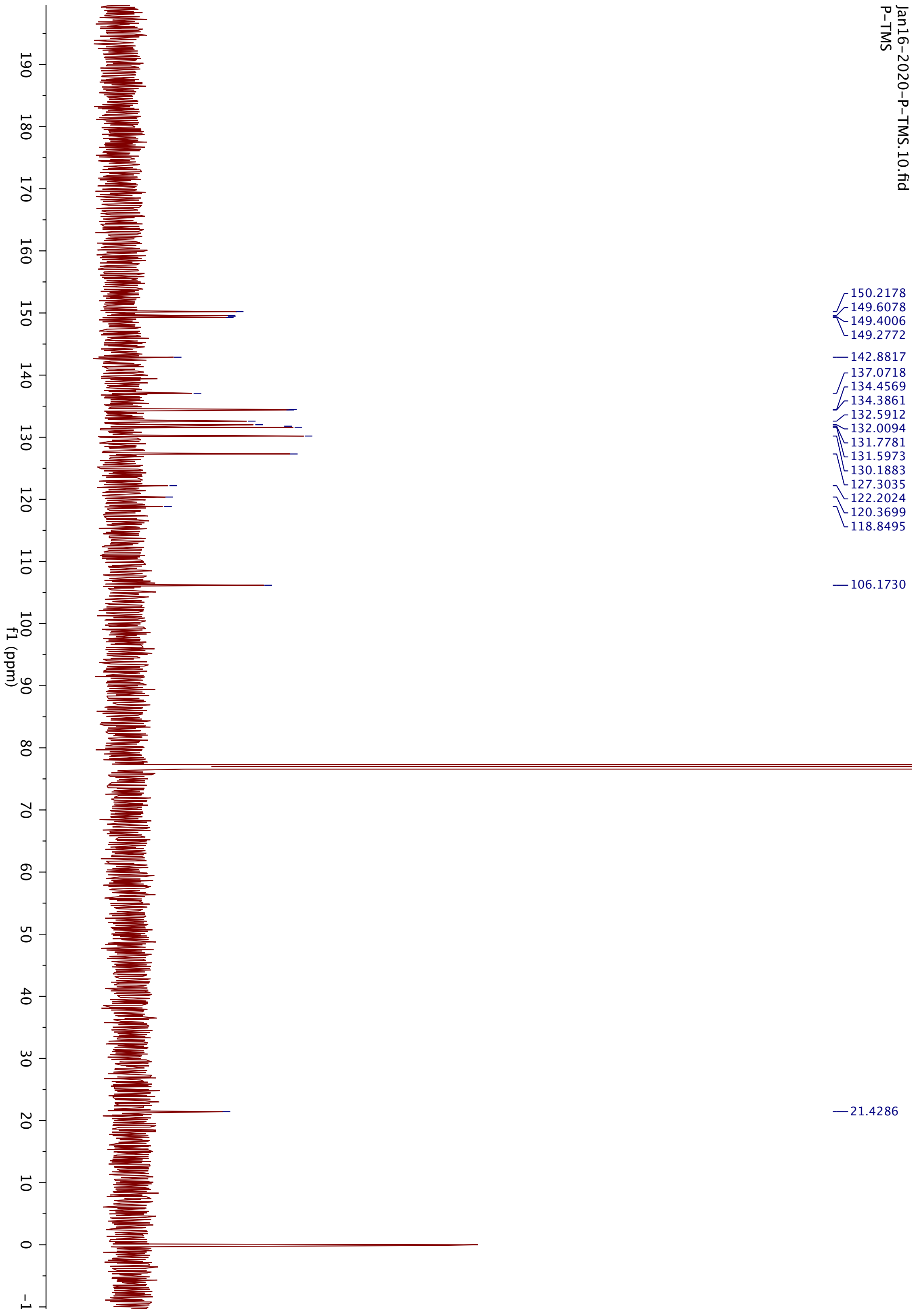

20: MALDI-MS

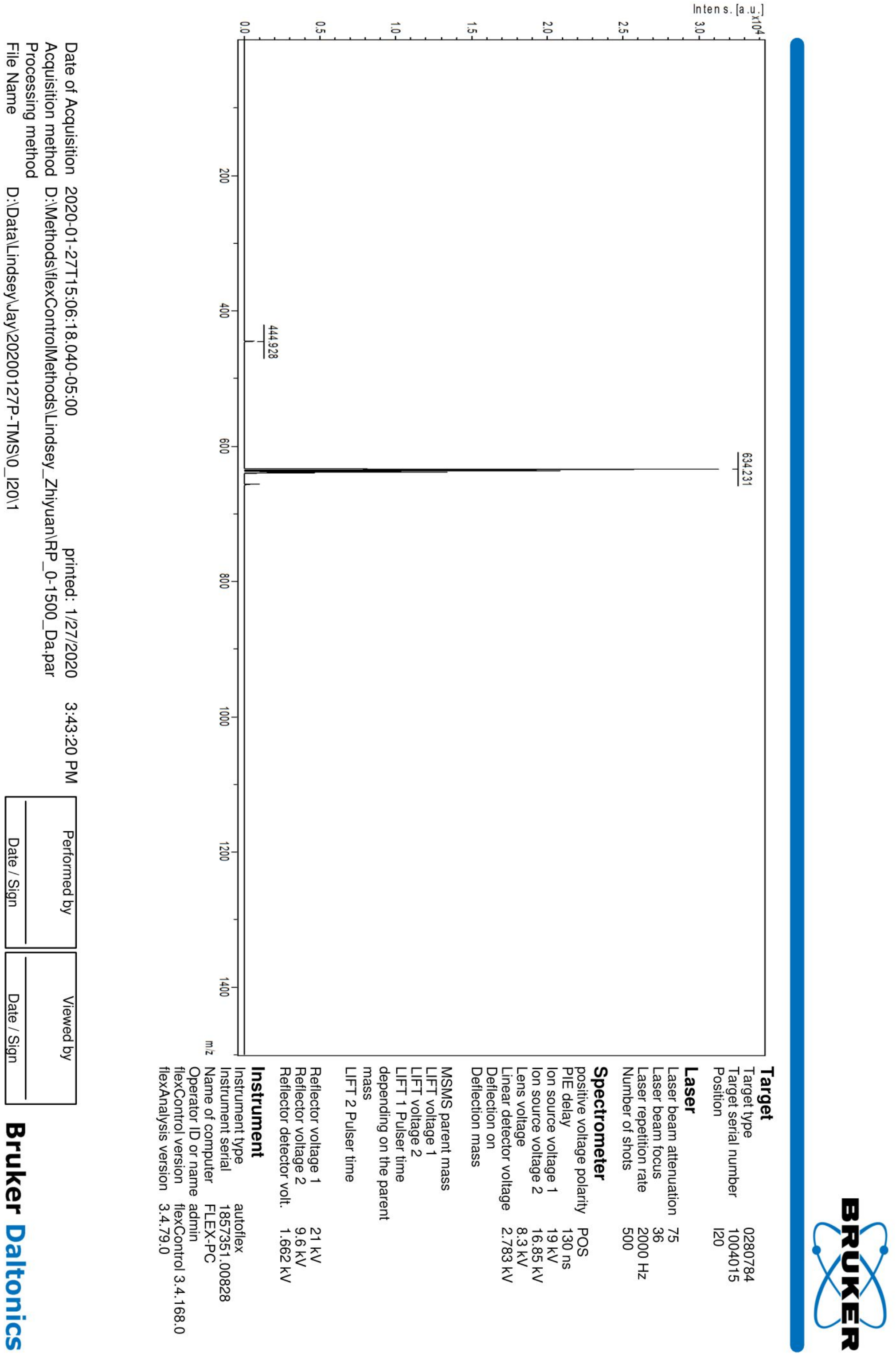

Inten s. [a.u.]
x10^4
3.0
2.5
2.0
1.5
1.0
0.5
0.0
200
400
600
800
1000
1200
1400
m/z
444.928
634.231
Date of Acquisition 2020-01-27T15:06:18.040-05:00 printed: 1/27/2020 3:43:20 PM
Acquisition method D:\Methods\flexControlMethods\Lindsey_Zhiyuan\RP_0-1500_Da.par
Processing method
File Name D:\Data\Lindsey\Jay\20200127P-TMS\0_I20\1
Performed by
Date / Sign
Viewed by
Date / Sign
Target
Target type 0280784
Target serial number 1004015
Position I20
Laser
Laser beam attenuation 75
Laser beam focus 36
Laser repetition rate 2000 Hz
Number of shots 500
Spectrometer
positive voltage polarity POS
PIE delay 130 ns
Ion source voltage 1 19 kV
Ion source voltage 2 16.85 kV
Lens voltage 8.3 kV
Linear detector voltage 2.783 kV
Deflection on
Deflection mass
MSMS parent mass
LIFT voltage 1
LIFT voltage 2
LIFT 1 Pulser time
depending on the parent mass
LIFT 2 Pulser time
Reflector voltage 1 21 kV
Reflector voltage 2 9.6 kV
Reflector detector volt. 1.662 kV
Instrument
Instrument type autoflex
Instrument serial 1857351.00828
Name of computer FLEX-PC
Operator ID or name admin
flexControl version flexControl 3.4.168.0
flexAnalysis version 3.4.79.0
Bruker Daltonics

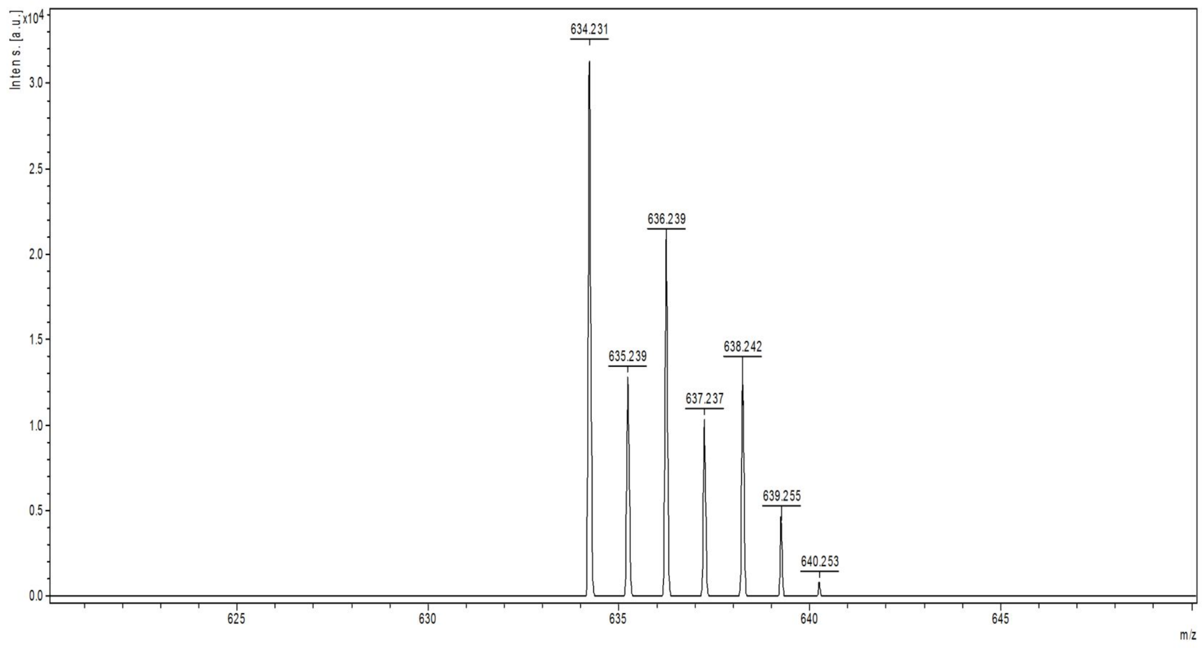

Target

Target type	0280784
Target serial number	1004015
Position	I20

Laser

Laser beam attenuation	75
Laser beam focus	36
Laser repetition rate	2000 Hz
Number of shots	500

Spectrometer

positive voltage polarity	POS
PIE delay	130 ns
Ion source voltage 1	19 kV
Ion source voltage 2	16.85 kV
Lens voltage	8.3 kV
Linear detector voltage	2.783 kV
Deflection on	
Deflection mass	
MSMS parent mass	
LIFT voltage 1	
LIFT voltage 2	
LIFT 1 Pulser time depending on the parent mass	
LIFT 2 Pulser time	
Reflector voltage 1	21 kV
Reflector voltage 2	9.6 kV
Reflector detector volt.	1.662 kV

Instrument

Instrument type	autoflex
Instrument serial	1857351.00828
Name of computer	FLEX-PC
Operator ID or name	admin
flexControl version	flexControl 3.4.168.0
flexAnalysis version	3.4.79.0

Date of Acquisition 2020-01-27T15:06:18.040-05:00 printed: 1/27/2020 3:42:57 PM
Acquisition method D:\Methods\flexControlMethods\Lindsey_Zhiyuan\RP_0-1500_Da.par
Processing method
File Name D:\Data\Lindsey\Jay\20200127P-TMS\0_I20\1

Performed by	Viewed by
Date / Sign	Date / Sign

Bruker Daltonics

20: Absorption spectrum (toluene)

Spectrum/Peak Report   Date 9/23/2022 Time 12:01:14 Page 1 of 1

Method file : <method not saved>
Information : Default Method
Data File : C:\Chem32\1\DATA\JayRong\Solar_Cell\ABS for paper\P-TMS_1.SD
Created : 1/17/20 15:33:49

Compound 20

Overlaid Spectra:

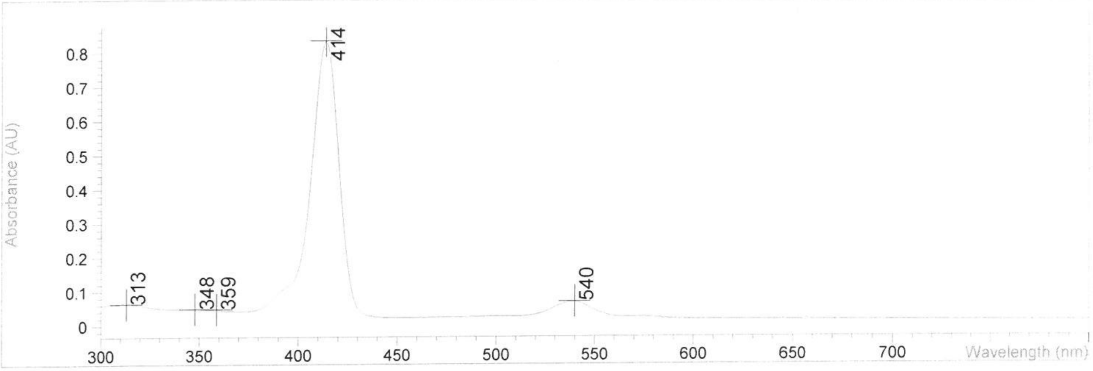

#	Name	Peaks(nm)	Abs(AU)
1		414.0	0.83416
1		540.0	6.8727E-2
1		313.0	6.4114E-2
1		348.0	4.8937E-2
1		359.0	4.7909E-2

Report generated by : jon   Signature: ....

*** End Spectrum/Peak Report ***

21: ^{1}H NMR, $CDCl_3$, 600 MHz

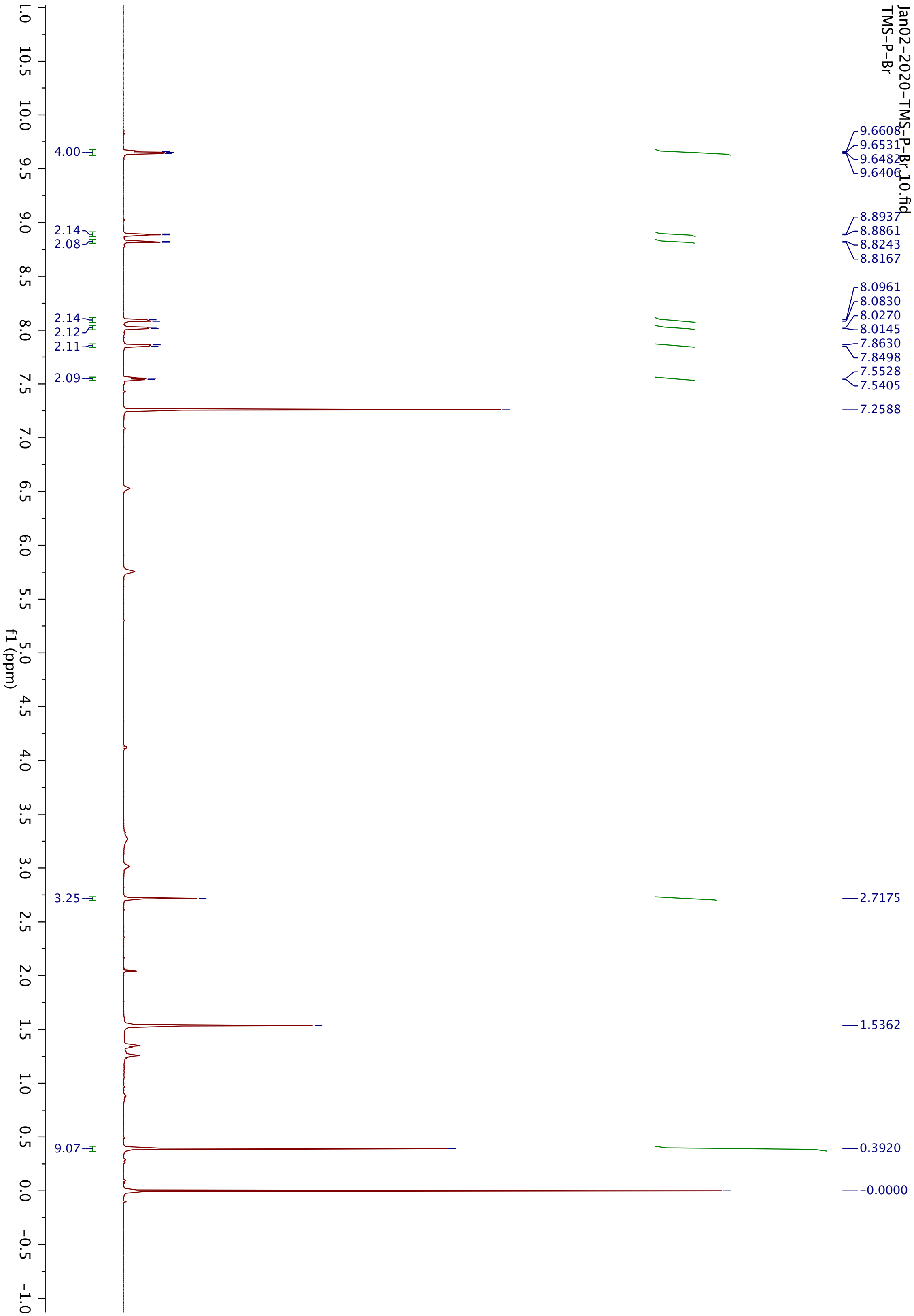

21: MALDI-MS

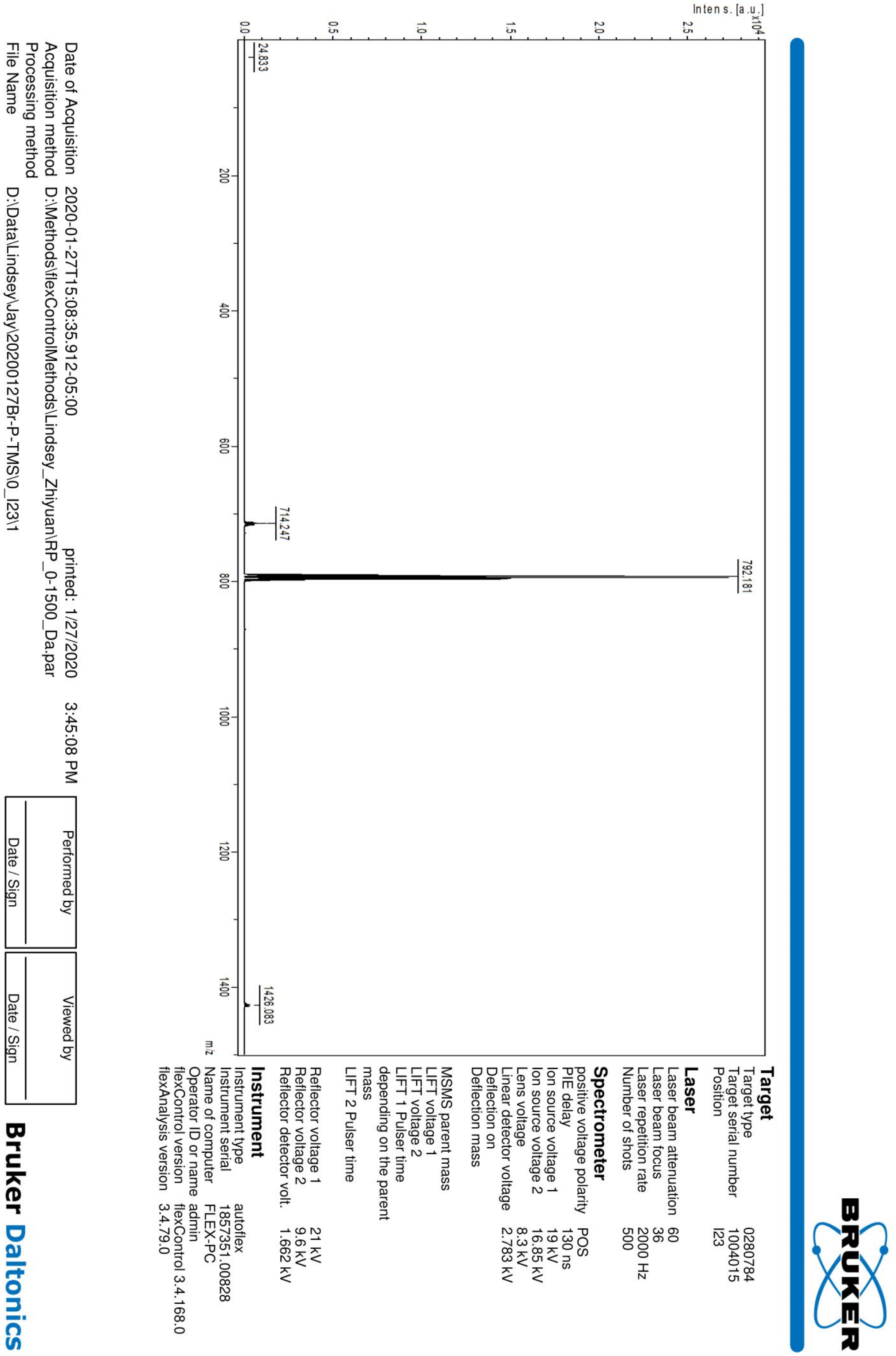

Inten s. [a.u.]
x10^4
0.0
0.5
1.0
1.5
2.0
2.5
24.833
714.247
792.181
1426.083
200
400
600
800
1000
1200
1400
m/z
Target
Target type 0280784
Target serial number 1004015
Position I23
Laser
Laser beam attenuation 60
Laser beam focus 36
Laser repetition rate 2000 Hz
Number of shots 500
Spectrometer
positive voltage polarity POS
PIE delay 130 ns
Ion source voltage 1 19 kV
Ion source voltage 2 16.85 kV
Lens voltage 8.3 kV
Linear detector voltage 2.783 kV
Deflection on
Deflection mass
MSMS parent mass
LIFT voltage 1
LIFT voltage 2
LIFT 1 Pulser time
depending on the parent mass
LIFT 2 Pulser time
Reflector voltage 1 21 kV
Reflector voltage 2 9.6 kV
Reflector detector volt. 1.662 kV
Instrument
Instrument type autoflex
Instrument serial 1857351.00828
Name of computer FLEX-PC
Operator ID or name admin
flexControl version flexControl 3.4.168.0
flexAnalysis version 3.4.79.0
Date of Acquisition 2020-01-27T15:08:35.912-05:00
printed: 1/27/2020 3:45:08 PM
Acquisition method D:\Methods\flexControlMethods\Lindsey_Zhiyuan\RP_0-1500_Da.par
Processing method
File Name D:\Data\Lindsey\Jay\20200127Br-P-TMS\0_I23\1
Performed by
Date / Sign
Viewed by
Date / Sign
BRUKER
Bruker Daltonics

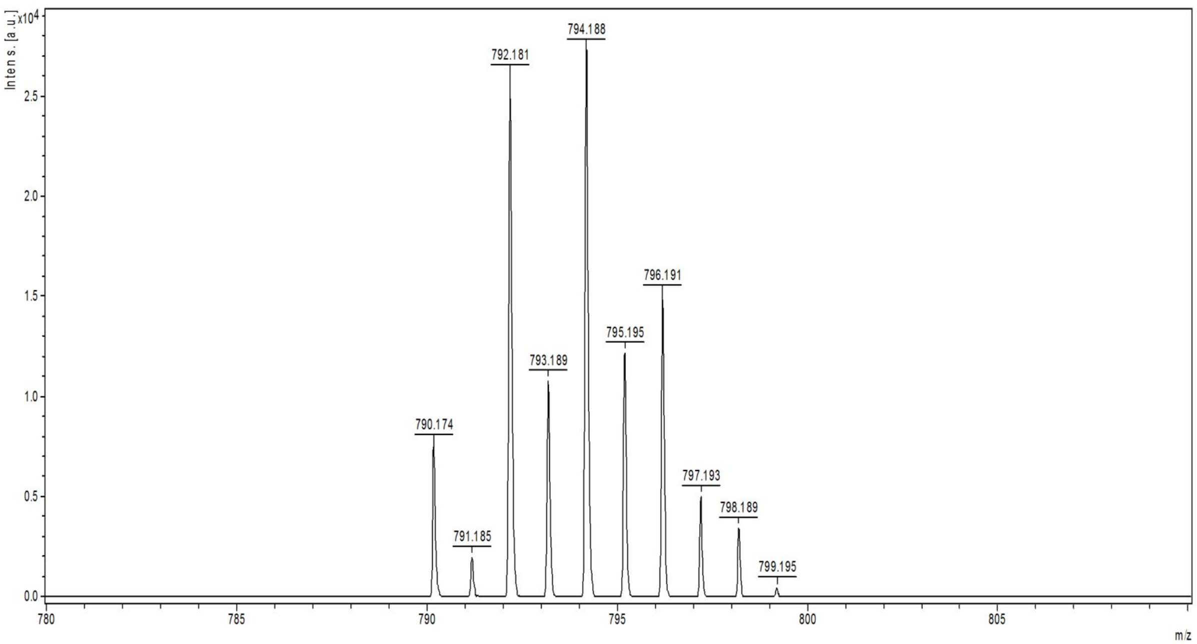

Target

Target type	0280784
Target serial number	1004015
Position	I23

Laser

Laser beam attenuation	60
Laser beam focus	36
Laser repetition rate	2000 Hz
Number of shots	500

Spectrometer

positive voltage polarity	POS
PIE delay	130 ns
Ion source voltage 1	19 kV
Ion source voltage 2	16.85 kV
Lens voltage	8.3 kV
Linear detector voltage	2.783 kV
Deflection on	
Deflection mass	
MSMS parent mass	
LIFT voltage 1	
LIFT voltage 2	
LIFT 1 Pulser time depending on the parent mass	
LIFT 2 Pulser time	
Reflector voltage 1	21 kV
Reflector voltage 2	9.6 kV
Reflector detector volt.	1.662 kV

Instrument

Instrument type	autoflex
Instrument serial	1857351.00828
Name of computer	FLEX-PC
Operator ID or name	admin
flexControl version	flexControl 3.4.168.0
flexAnalysis version	3.4.79.0

Date of Acquisition 2020-01-27T15:08:35.912-05:00 printed: 1/27/2020 3:44:46 PM
Acquisition method D:\Methods\flexControlMethods\Lindsey_Zhiyuan\RP_0-1500_Da.par
Processing method
File Name D:\Data\Lindsey\Jay\20200127Br-P-TMS\0_I23\1

Performed by	Viewed by
Date / Sign	Date / Sign

Bruker Daltonics

21: Absorption spectrum (toluene)

Spectrum/Peak Report Date 9/23/2022 Time 12:00:48 Page 1 of 1

Method file : <method not saved>
Information : Default Method
Data File : C:\Chem32\1\DATA\JayRong\Solar_Cell\ABS for paper\BR-P-TMS_1.SD
Created : 1/17/20 15:31:04

Compound 21

Overlaid Spectra:

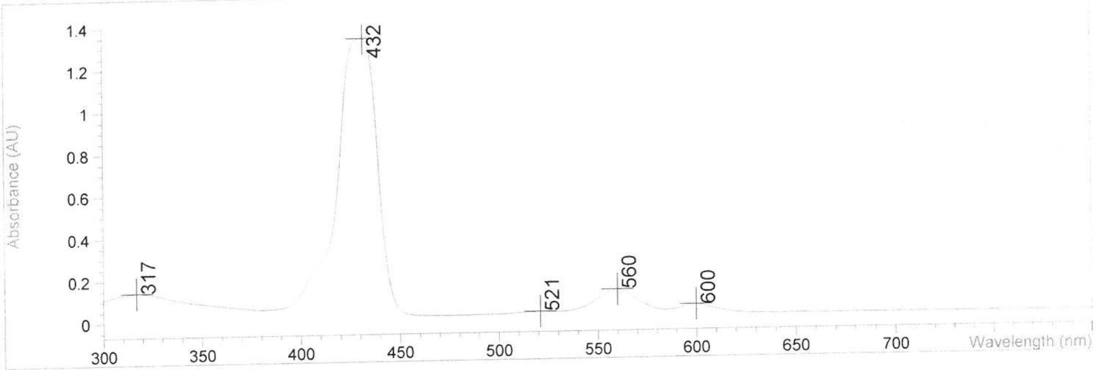

#	Name	Peaks(nm)	Abs(AU)
1		432.0	1.33510
1		317.0	0.14086
1		560.0	0.12602
1		600.0	4.9747E-2
1		521.0	2.6982E-2

Report generated by : jon Signature: ....

*** End Spectrum/Peak Report ***

22: ^{1}H NMR, $CDCl_3$, 600 MHz

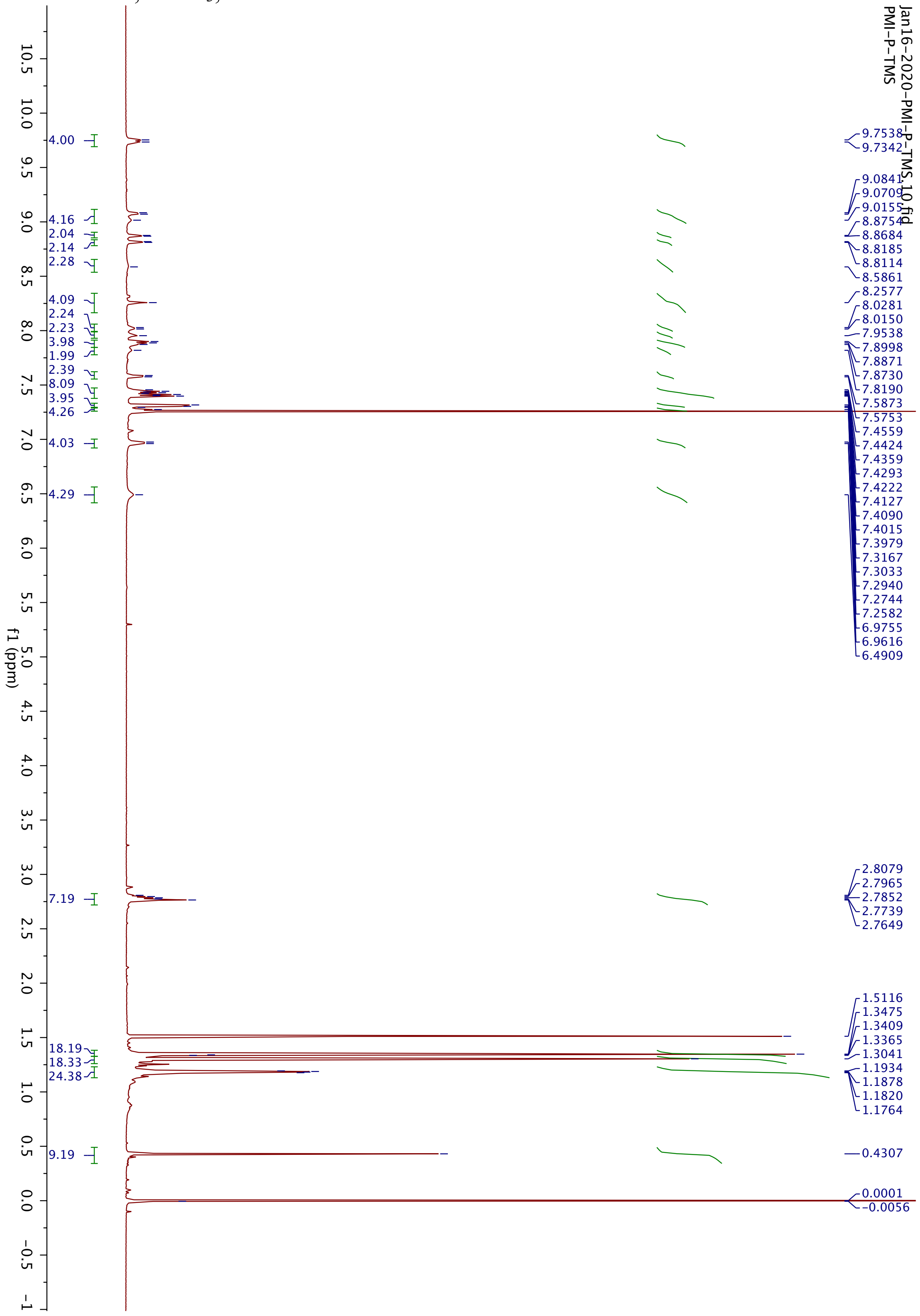

22: MALDI-MS

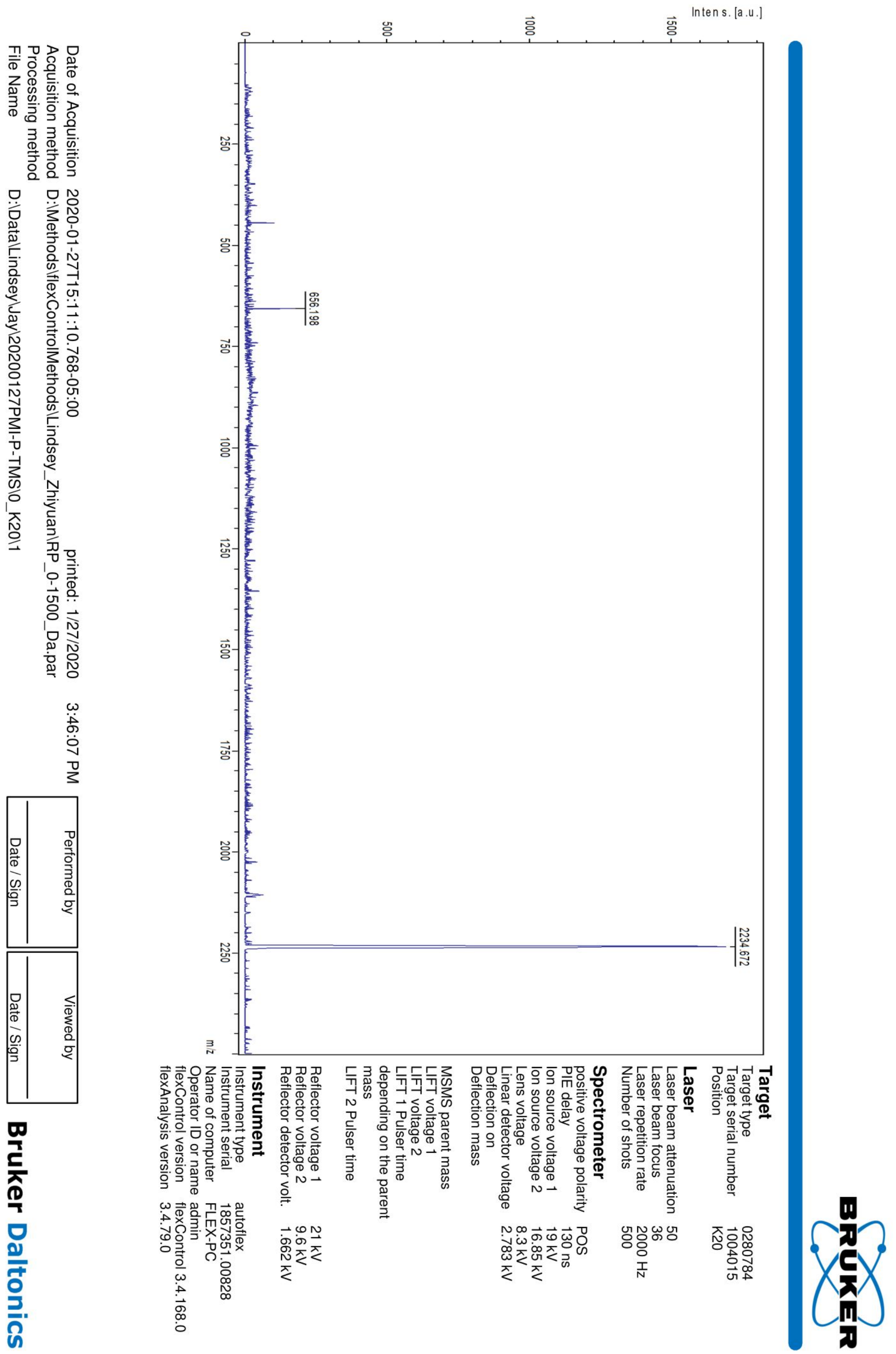

Intens. [a.u.]
656.198
2234.672
m/z
Target
Target type 0280784
Target serial number 1004015
Position K20
Laser
Laser beam attenuation 50
Laser beam focus 36
Laser repetition rate 2000 Hz
Number of shots 500
Spectrometer
positive voltage polarity POS
PIE delay 130 ns
Ion source voltage 1 19 kV
Ion source voltage 2 16.85 kV
Lens voltage 8.3 kV
Linear detector voltage 2.783 kV
Deflection on
Deflection mass
MSMS parent mass
LIFT voltage 1
LIFT voltage 2
LIFT 1 Pulser time
depending on the parent mass
LIFT 2 Pulser time
Reflector voltage 1 21 kV
Reflector voltage 2 9.6 kV
Reflector detector volt. 1.662 kV
Instrument
Instrument type autoflex
Instrument serial 1857351.00828
Name of computer FLEX-PC
Operator ID or name admin
flexControl version flexControl 3.4.168.0
flexAnalysis version 3.4.79.0
Date of Acquisition 2020-01-27T15:11:10.768-05:00
printed: 1/27/2020 3:46:07 PM
Acquisition method D:\Methods\flexControlMethods\Lindsey_Zhiyuan\RP_0-1500_Da.par
Processing method
File Name D:\Data\Lindsey\Jay\20200127PMI-P-TMS\0_K20\1
Performed by
Date / Sign
Viewed by
Date / Sign
Bruker Daltonics

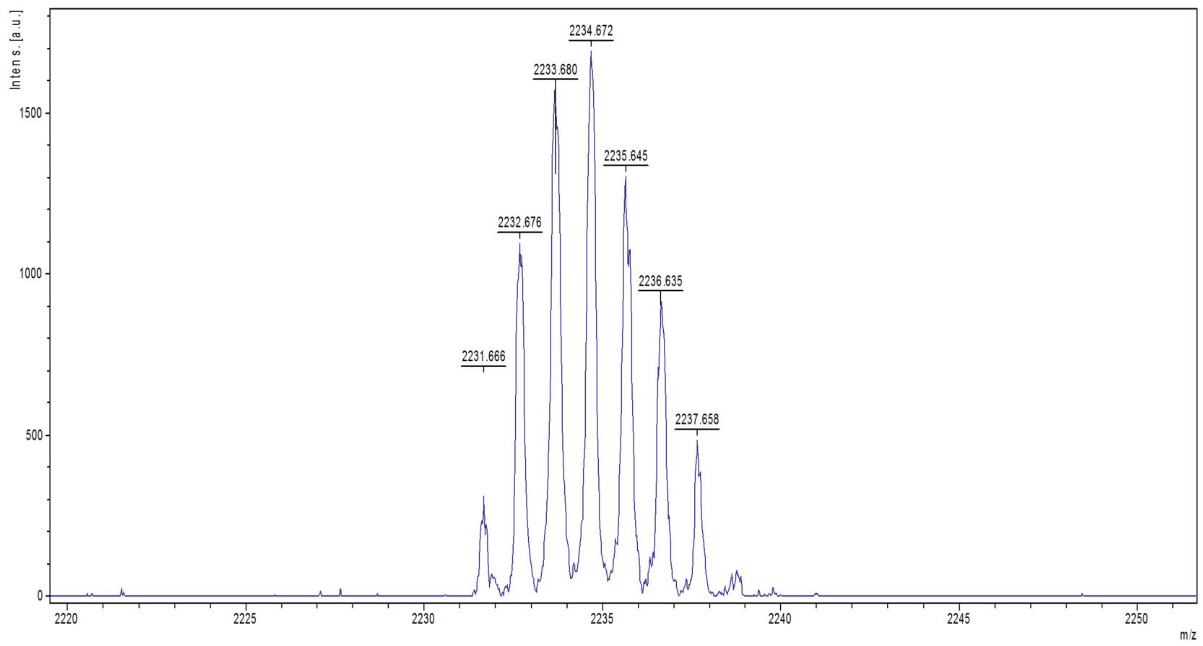

Target

Target type	0280784
Target serial number	1004015
Position	K20

Laser

Laser beam attenuation	50
Laser beam focus	36
Laser repetition rate	2000 Hz
Number of shots	500

Spectrometer

positive voltage polarity	POS
PIE delay	130 ns
Ion source voltage 1	19 kV
Ion source voltage 2	16.85 kV
Lens voltage	8.3 kV
Linear detector voltage	2.783 kV
Deflection on	
Deflection mass	
MSMS parent mass	
LIFT voltage 1	
LIFT voltage 2	
LIFT 1 Pulser time depending on the parent mass	
LIFT 2 Pulser time	
Reflector voltage 1	21 kV
Reflector voltage 2	9.6 kV
Reflector detector volt.	1.662 kV

Instrument

Instrument type	autoflex
Instrument serial	1857351.00828
Name of computer	FLEX-PC
Operator ID or name	admin
flexControl version	flexControl 3.4.168.0
flexAnalysis version	3.4.79.0

Date of Acquisition	2020-01-27T15:11:10.768-05:00	printed: 1/27/2020	3:45:59 PM
Acquisition method	D:\Methods\flexControlMethods\Lindsey_Zhiyuan\RP_0-1500_Da.par		
Processing method			
File Name	D:\Data\Lindsey\Jay\20200127PMI-P-TMS\0_K20\1		

Performed by	Viewed by
Date / Sign	Date / Sign

Bruker Daltonics

22: Absorption spectrum (toluene)

```
=================================================================================
Spectrum/Peak Report                            Date 9/23/2022 Time 12:01:49  Page   1 of 1
=================================================================================

Method file :          <method not saved>
Information :          Default Method
Data File   :          C:\Chem32\1\DATA\JayRong\Solar_Cell\ABS for paper\PMI-P-TMS_1.SD
                         Created : 1/17/20 15:21:06
```

compound 22

Overlaid Spectra:

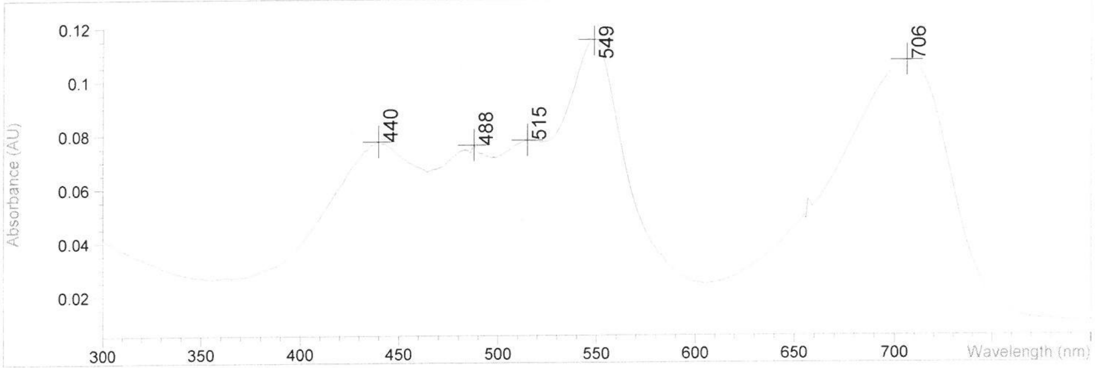

#	Name	Peaks(nm)	Abs(AU)
1		549.0	0.11524
1		706.0	0.10732
1		515.0	7.7922E-2
1		440.0	7.7526E-2
1		488.0	7.6225E-2

Report generated by : jon   Signature: ....

*** End Spectrum/Peak Report ***

23: ^{1}H NMR, $CDCl_3$, 600 MHz

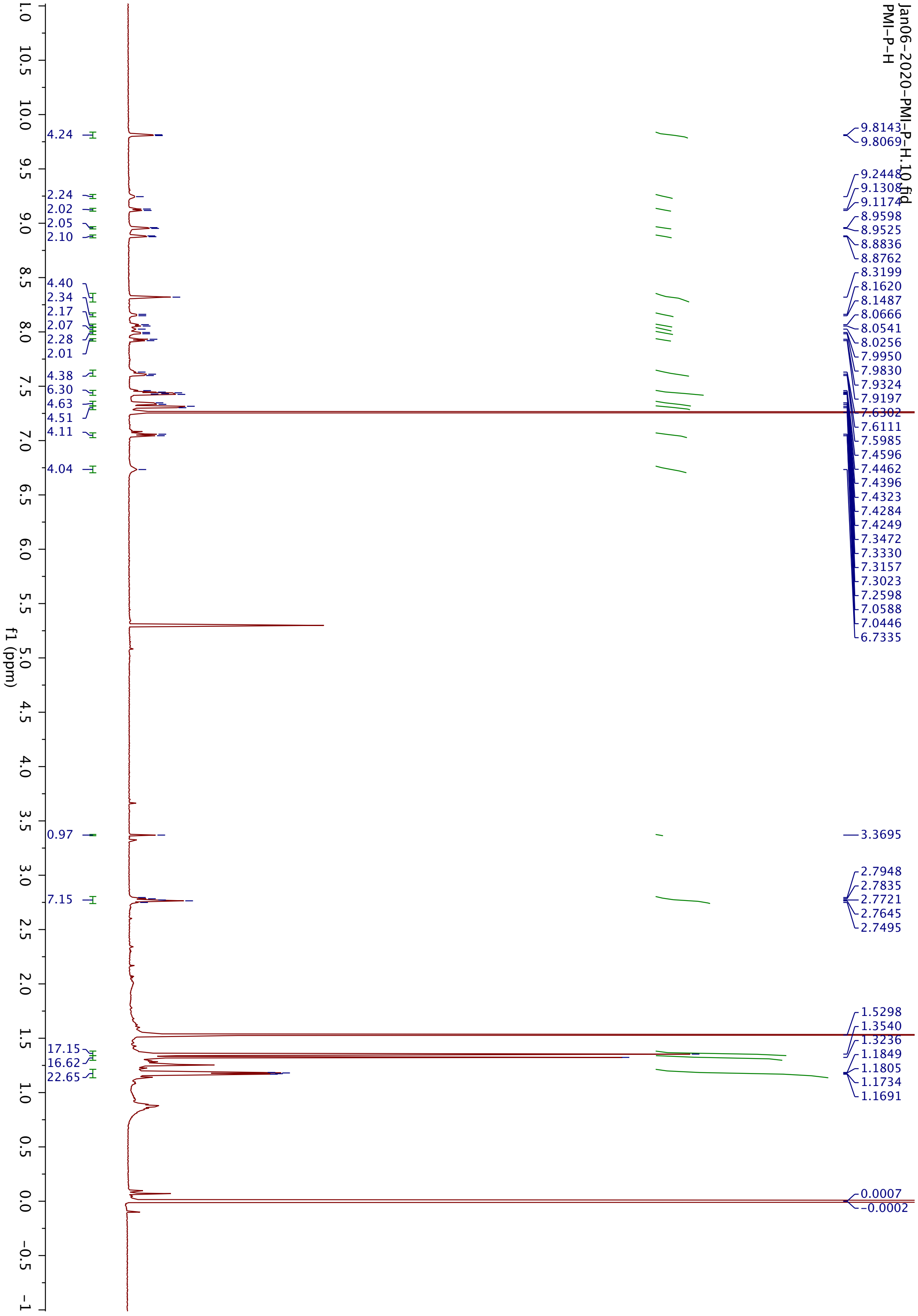

23: MALDI-MS

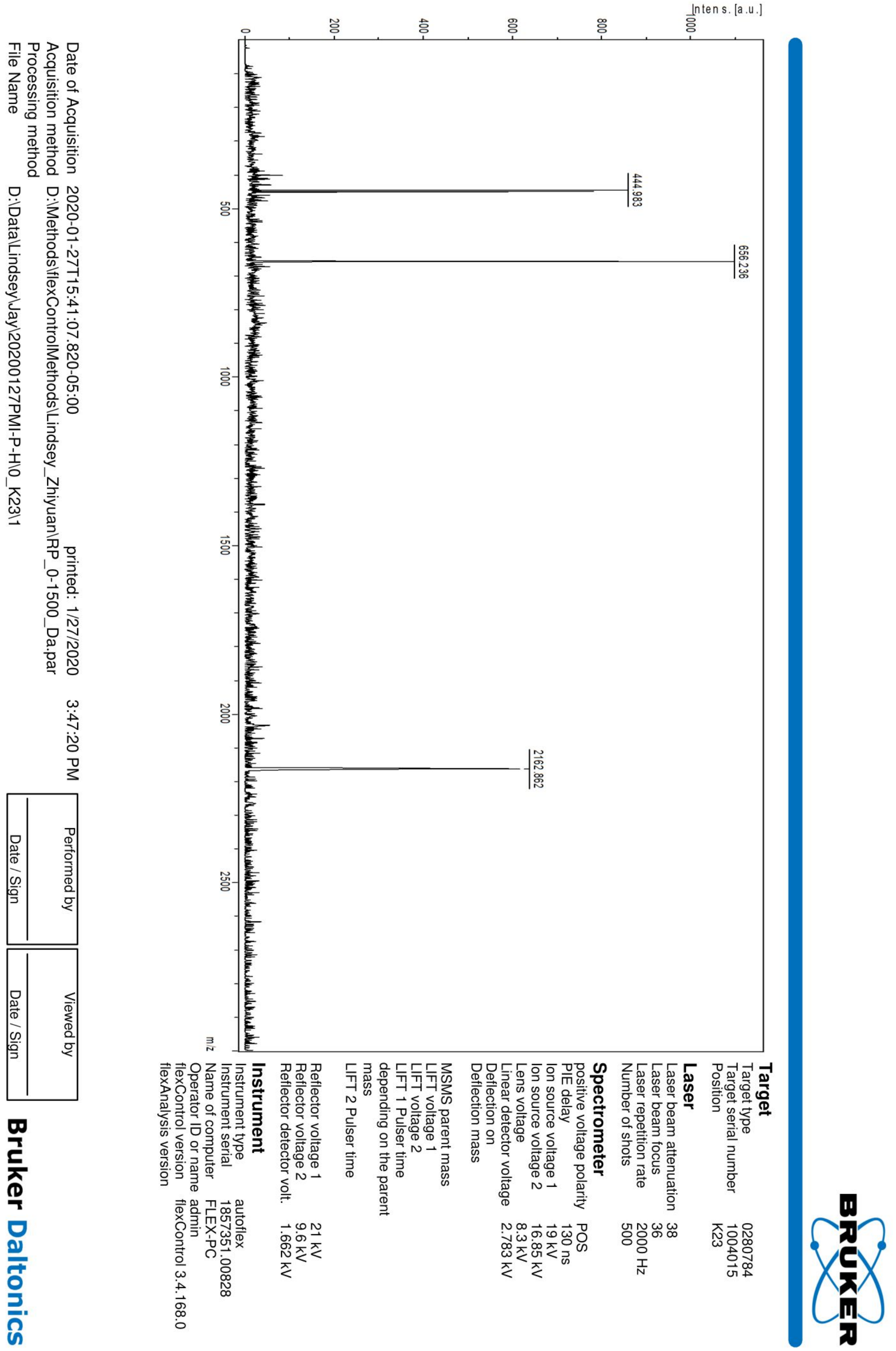

Intens. [a.u.]
1000
800
600
400
200
0
444.983
656.236
2162.862
500
1000
1500
2000
2500
m/z
Target
Target type 0280784
Target serial number 1004015
Position K23
Laser
Laser beam attenuation 38
Laser beam focus 36
Laser repetition rate 2000 Hz
Number of shots 500
Spectrometer
positive voltage polarity POS
PIE delay 130 ns
Ion source voltage 1 19 kV
Ion source voltage 2 16.85 kV
Lens voltage 8.3 kV
Linear detector voltage 2.783 kV
Deflection on
Deflection mass
MSMS parent mass
LIFT voltage 1
LIFT voltage 2
LIFT 1 Pulser time
depending on the parent mass
LIFT 2 Pulser time
Reflector voltage 1 21 kV
Reflector voltage 2 9.6 kV
Reflector detector volt. 1.662 kV
Instrument
Instrument type autoflex
Instrument serial 1857351.00828
Name of computer FLEX-PC
Operator ID or name admin
flexControl version flexControl 3.4.168.0
flexAnalysis version
Date of Acquisition 2020-01-27T15:41:07.820-05:00 printed: 1/27/2020 3:47:20 PM
Acquisition method D:\Methods\flexControlMethods\Lindsey_Zhiyuan\RP_0-1500_Da.par
Processing method
File Name D:\Data\Lindsey\Jay\20200127PMI-P-H\0_K23\1
Performed by
Date / Sign
Viewed by
Date / Sign
Bruker Daltonics

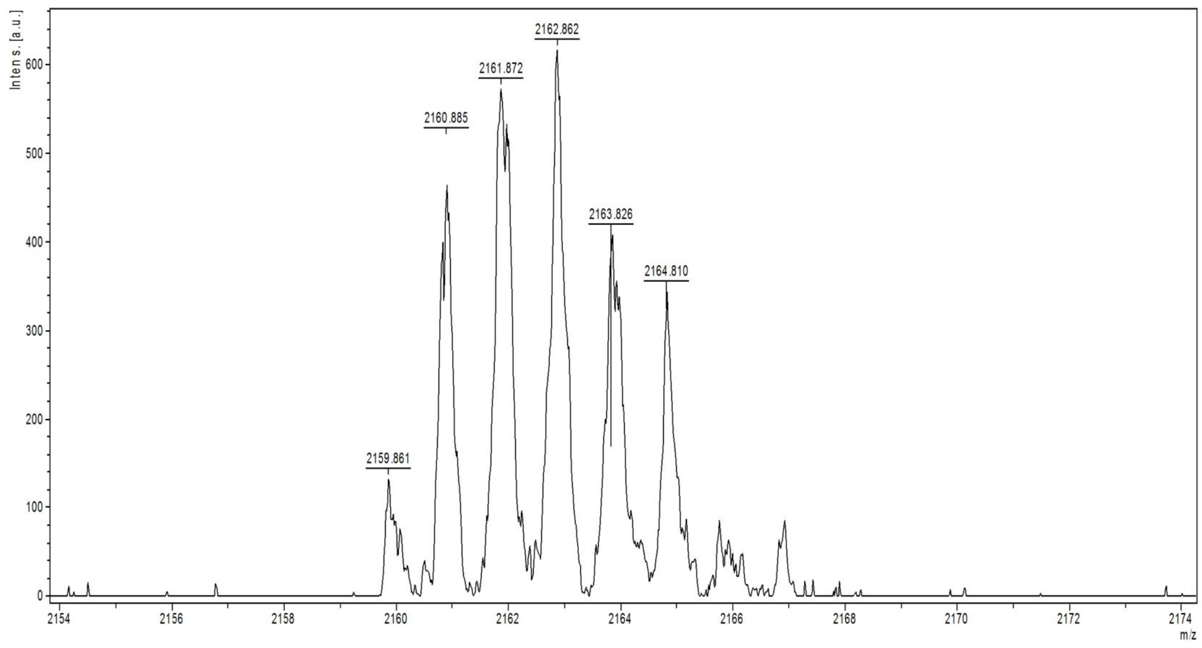

Target

Target type	0280784
Target serial number	1004015
Position	K23

Laser

Laser beam attenuation	38
Laser beam focus	36
Laser repetition rate	2000 Hz
Number of shots	500

Spectrometer

positive voltage polarity	POS
PIE delay	130 ns
Ion source voltage 1	19 kV
Ion source voltage 2	16.85 kV
Lens voltage	8.3 kV
Linear detector voltage	2.783 kV
Deflection on	
Deflection mass	
MSMS parent mass	
LIFT voltage 1	
LIFT voltage 2	
LIFT 1 Pulser time depending on the parent mass	
LIFT 2 Pulser time	
Reflector voltage 1	21 kV
Reflector voltage 2	9.6 kV
Reflector detector volt.	1.662 kV

Instrument

Instrument type	autoflex
Instrument serial	1857351.00828
Name of computer	FLEX-PC
Operator ID or name	admin
flexControl version	flexControl 3.4.168.0
flexAnalysis version	

Date of Acquisition 2020-01-27T15:41:07.820-05:00 printed: 1/27/2020 3:47:10 PM
Acquisition method D:\Methods\flexControlMethods\Lindsey_Zhiyuan\RP_0-1500_Da.par
Processing method
File Name D:\Data\Lindsey\Jay\20200127PMI-P-H\0_K23\1

Performed by	Viewed by
Date / Sign	Date / Sign

Bruker Daltonics

23: Absorption spectrum (toluene)

Spectrum/Peak Report Date 9/23/2022 Time 12:01:55 Page 1 of 1

Method file : <method not saved>
Information : Default Method
Data File : C:\Chem32\1\DATA\JayRong\Solar_Cell\ABS for paper\PMI-P-H_1.SD
Created : 1/17/20 15:27:08

Compound 23

Overlaid Spectra:

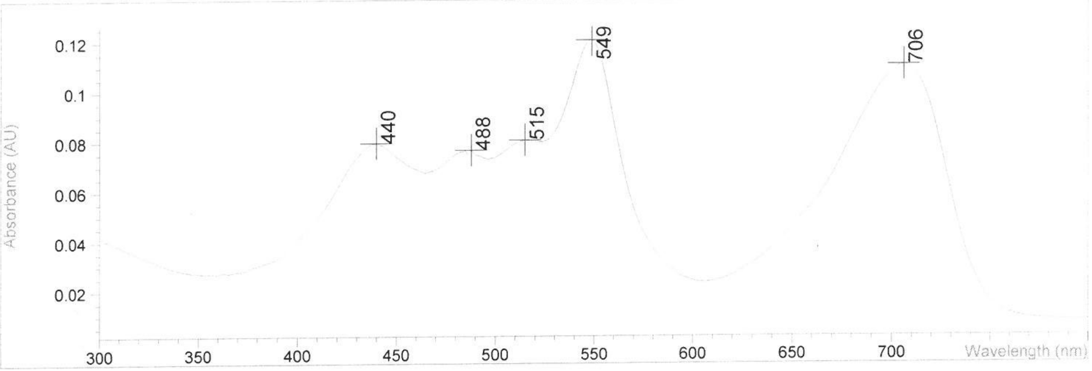

#	Name	Peaks(nm)	Abs(AU)
1		549.0	0.12052
1		706.0	0.11034
1		515.0	8.0578E-2
1		440.0	7.9451E-2
1		488.0	7.6578E-2

Report generated by : jon Signature: ....

*** End Spectrum/Peak Report ***

26: ^{1}H NMR, $CDCl_3$, 600 MHz

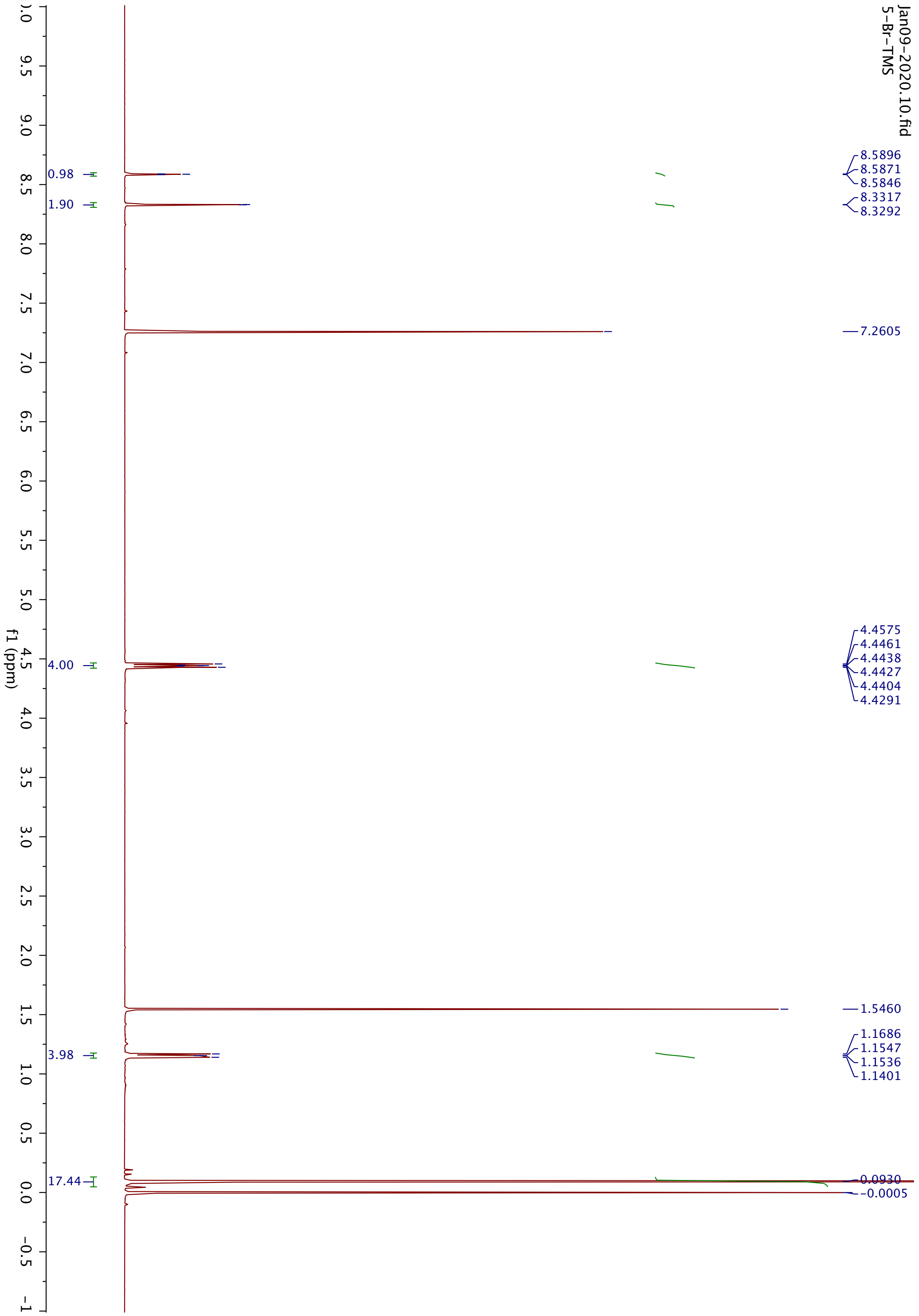

26: ^{13}C NMR, $CDCl_3$, 151 MHz

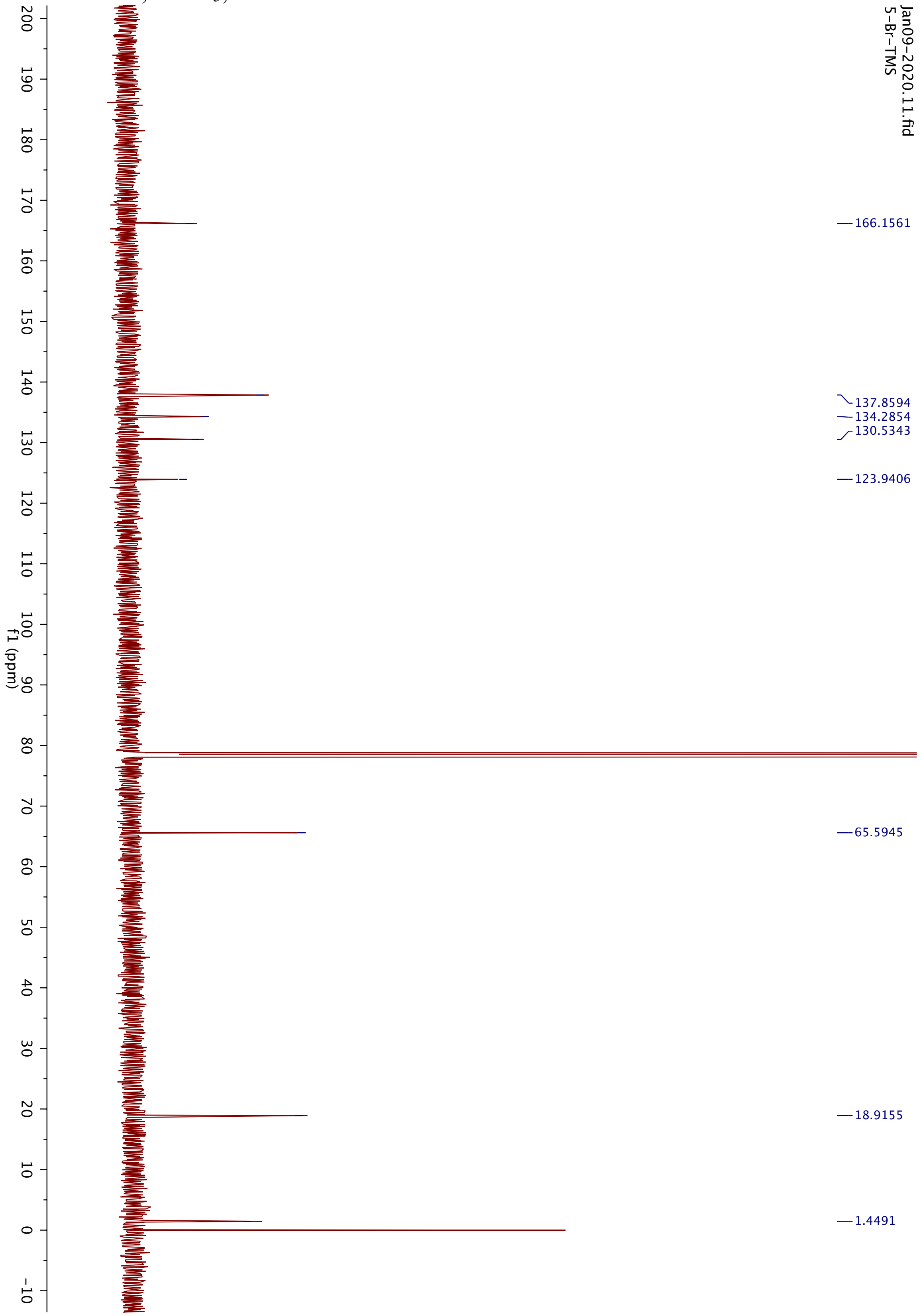

26: ESI-MS

NC STATE UNIVERSITY

Molecular Education, Technology and
Research Innovation Center
Raleigh, NC 27695
https://research.ncsu.edu/metric/

Br-I-TMS Experimental and Theoretical Isotopic Distribution for $C_{18}H_{29}BrO_4Si_2$ [M-H]$^-$

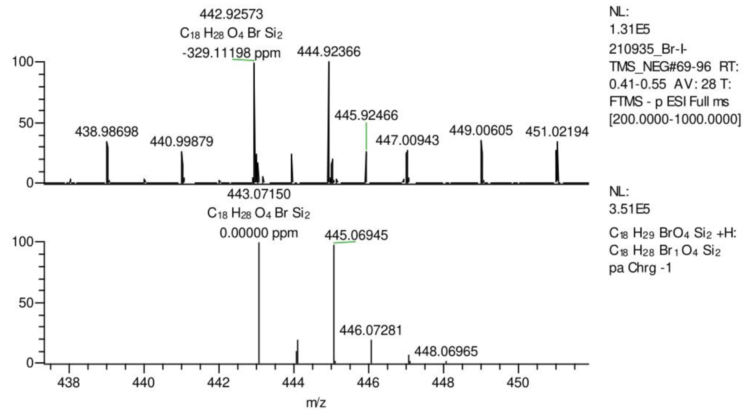

Br-I-TMS Scan 338-352 m/z

210935_Br-I-TMS_NEG #69-96 RT: 0.41-0.55 AV: 28 NL: 2.66E7
T: FTMS - p ESI Full ms [200.0000-1000.0000]

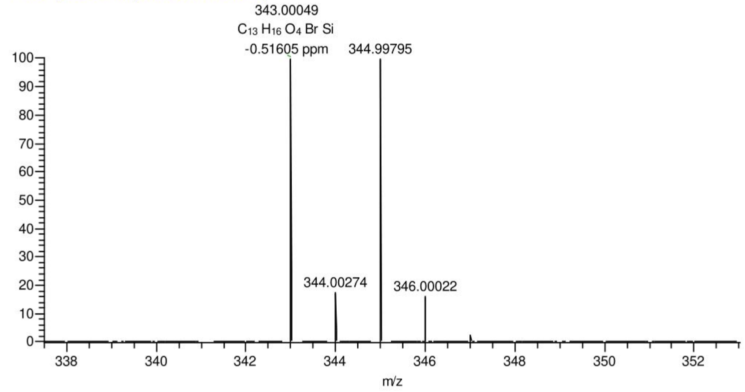

Page **1** of **1**

27: ^{1}H NMR, $CDCl_3$, 600 MHz

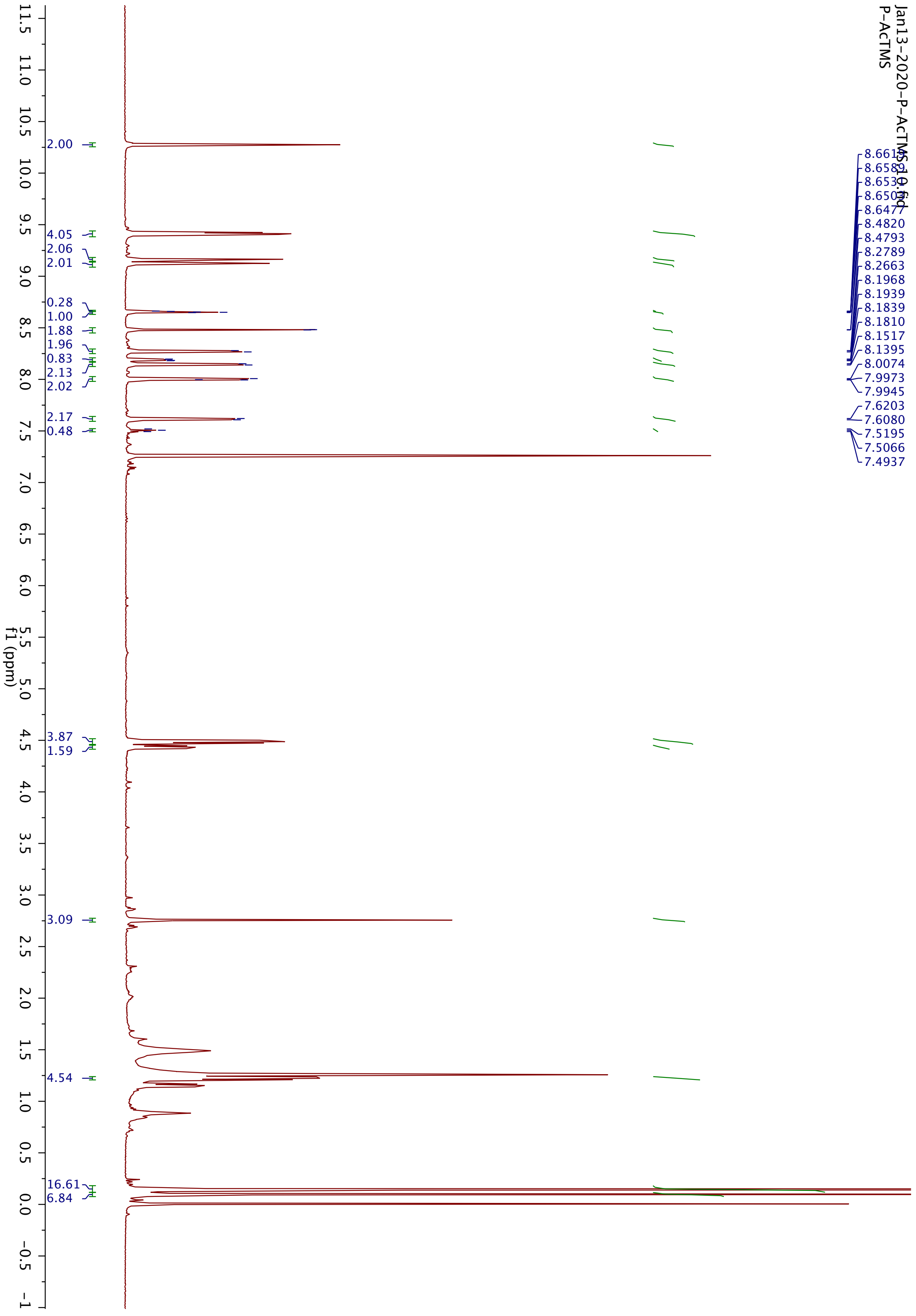

27: MALDI-MS

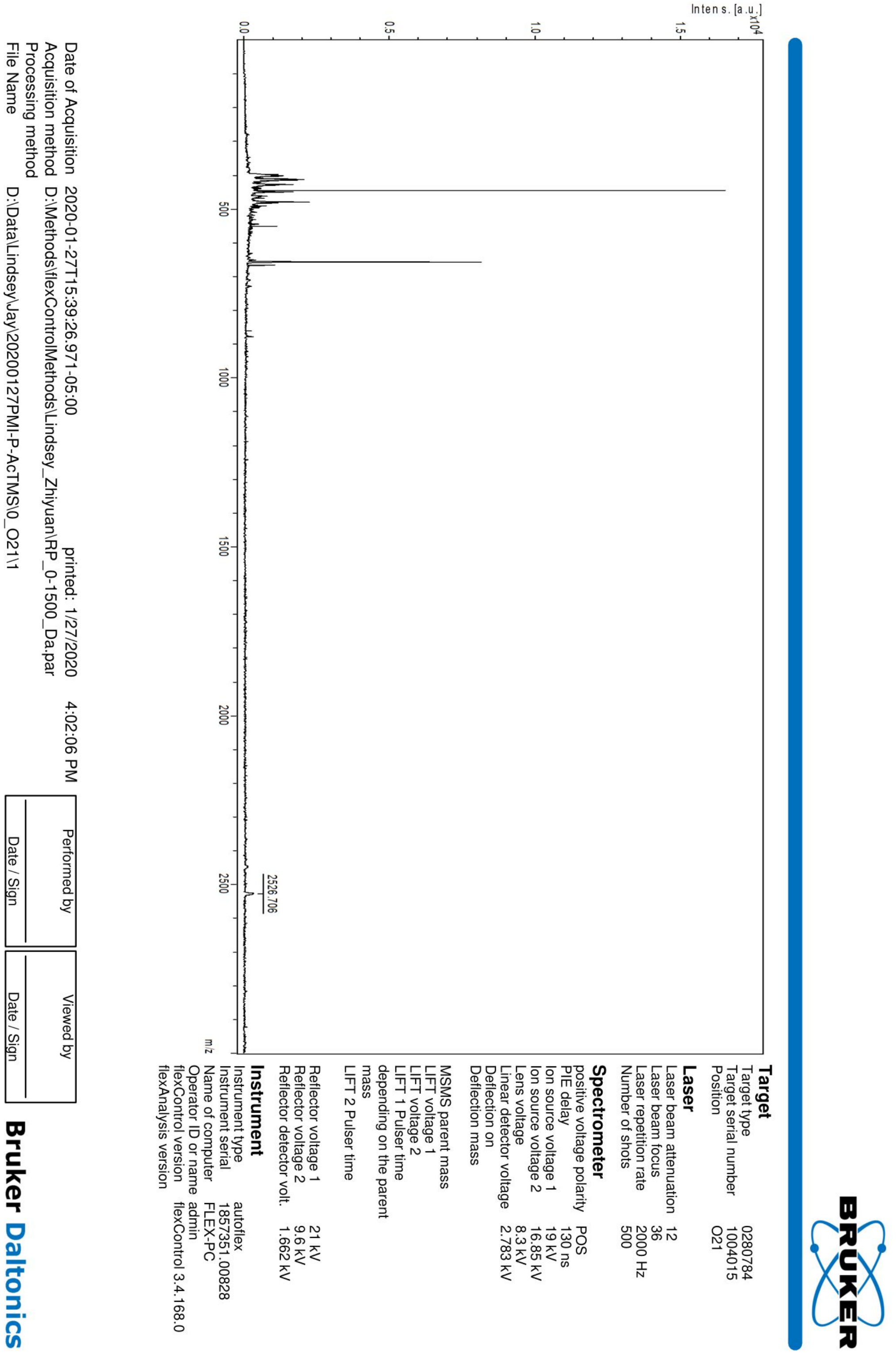

Inten s. [a.u.]
x10^4
1.5
1.0
0.5
0.0
500
1000
1500
2000
2500
m/z
2526.706
Target
Target type 0280784
Target serial number 1004015
Position O21
Laser
Laser beam attenuation 12
Laser beam focus 36
Laser repetition rate 2000 Hz
Number of shots 500
Spectrometer
positive voltage polarity POS
PIE delay 130 ns
Ion source voltage 1 19 kV
Ion source voltage 2 16.85 kV
Lens voltage 8.3 kV
Linear detector voltage 2.783 kV
Deflection on
Deflection mass
MSMS parent mass
LIFT voltage 1
LIFT voltage 2
LIFT 1 Pulser time
depending on the parent mass
LIFT 2 Pulser time
Reflector voltage 1 21 kV
Reflector voltage 2 9.6 kV
Reflector detector volt. 1.662 kV
Instrument
Instrument type autoflex
Instrument serial 1857351.00828
Name of computer FLEX-PC
Operator ID or name admin
flexControl version flexControl 3.4.168.0
flexAnalysis version
Date of Acquisition 2020-01-27T15:39:26.971-05:00 printed: 1/27/2020 4:02:06 PM
Acquisition method D:\Methods\flexControlMethods\Lindsey_Zhiyuan\RP_0-1500_Da.par
Processing method
File Name D:\Data\Lindsey\Jay\20200127PMI-P-AcTMS\0_O21\1
Performed by
Date / Sign
Viewed by
Date / Sign
Bruker Daltonics

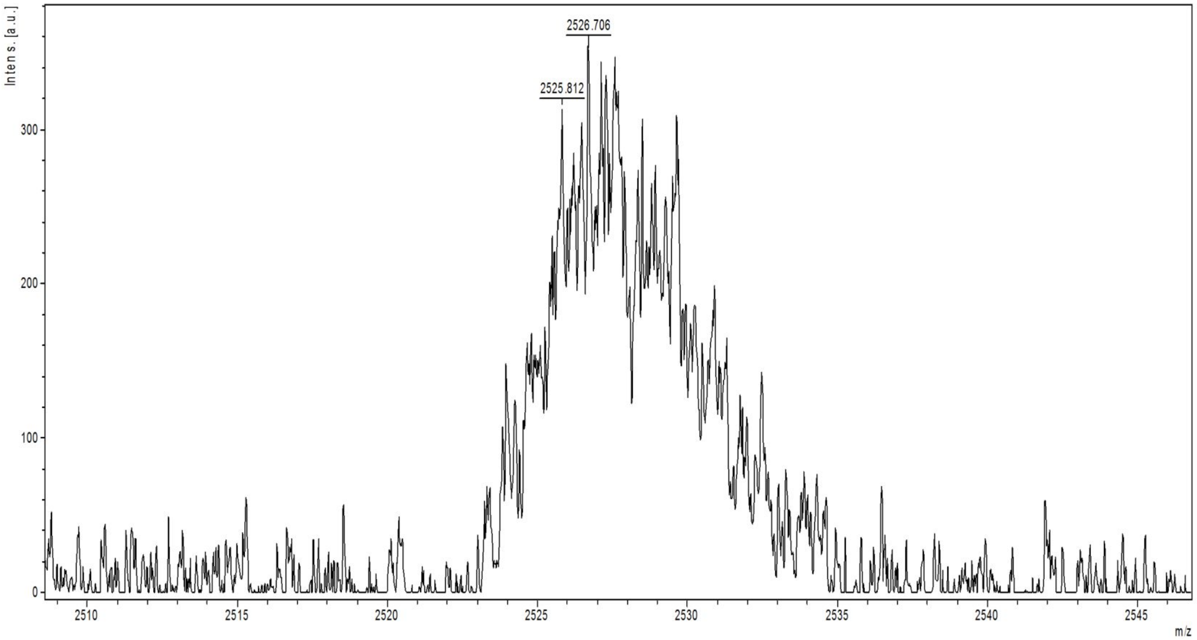

Target

Target type	0280784
Target serial number	1004015
Position	O21

Laser

Laser beam attenuation	12
Laser beam focus	36
Laser repetition rate	2000 Hz
Number of shots	500

Spectrometer

positive voltage polarity	POS
PIE delay	130 ns
Ion source voltage 1	19 kV
Ion source voltage 2	16.85 kV
Lens voltage	8.3 kV
Linear detector voltage	2.783 kV
Deflection on	
Deflection mass	
MSMS parent mass	
LIFT voltage 1	
LIFT voltage 2	
LIFT 1 Pulser time depending on the parent mass	
LIFT 2 Pulser time	
Reflector voltage 1	21 kV
Reflector voltage 2	9.6 kV
Reflector detector volt.	1.662 kV

Instrument

Instrument type	autoflex
Instrument serial	1857351.00828
Name of computer	FLEX-PC
Operator ID or name	admin
flexControl version	flexControl 3.4.168.0
flexAnalysis version	

Date of Acquisition	2020-01-27T15:39:26.971-05:00 printed: 1/27/2020 4:02:51 PM
Acquisition method	D:\Methods\flexControlMethods\Lindsey_Zhiyuan\RP_0-1500_Da.par
Processing method	
File Name	D:\Data\Lindsey\Jay\20200127PMI-P-AcTMS\0_O21\1

Performed by	Viewed by
Date / Sign	Date / Sign

Bruker Daltonics

27: Absorption spectrum (toluene)

```
===================================================================================
Spectrum/Peak Report                           Date 9/23/2022 Time 12:02:02  Page   1 of 1
===================================================================================


Method file :          <method not saved>
Information :          Default Method
Data File   :          C:\Chem32\1\DATA\JayRong\Solar_Cell\ABS for paper\PMI-P-ACTMS_1.SD
                           Created : 1/17/20 15:16:12

Overlaid Spectra:
```

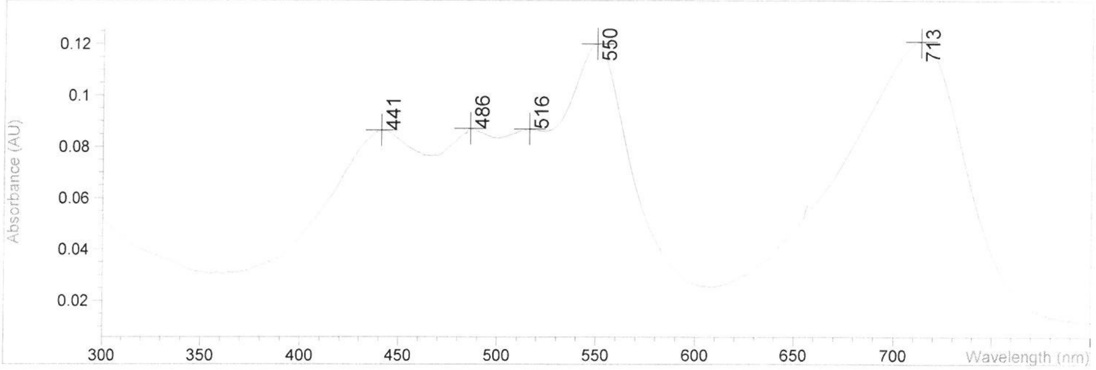

#	Name	Peaks(nm)	Abs(AU)
1		713.0	0.12108
1		550.0	0.12002
1		486.0	8.7032E-2
1		516.0	8.6899E-2
1		441.0	8.6447E-2

Report generated by : jon   Signature: ....

*** End Spectrum/Peak Report ***

28: Absorption spectrum (toluene)

Spectrum/Peak Report   Date 9/24/2022 Time 15:26:47 Page 1 of 1

Method file : <method not saved>
Information : Default Method
Data File : C:\Chem32\1\DATA\Masa\PERYLENE TRIAD 28 J8 (75).SD Created : 9/24/22 13:10:57

Overlaid Spectra:

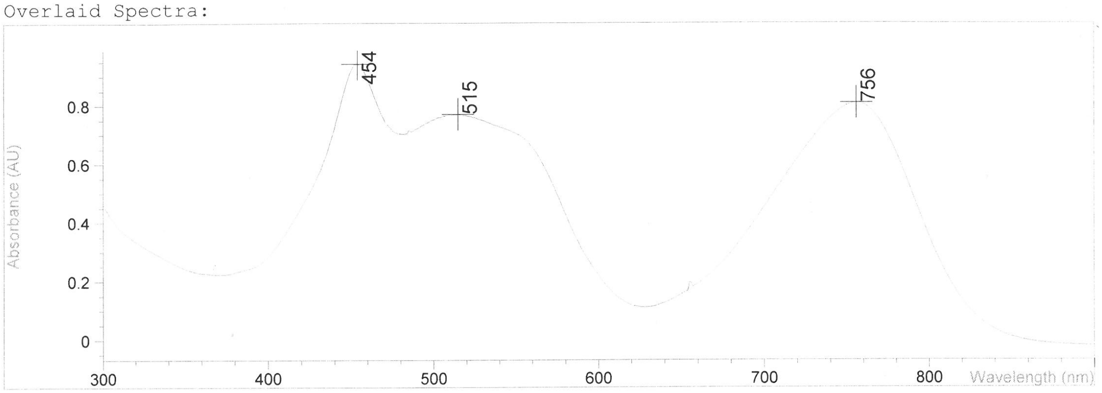

#	Name	Peaks(nm)	Abs(AU)
1		454.0	0.94268
1		756.0	0.80966
1		515.0	0.77060

Report generated by : jon   Signature: ....

*** End Spectrum/Peak Report ***

29: MALDI-MS

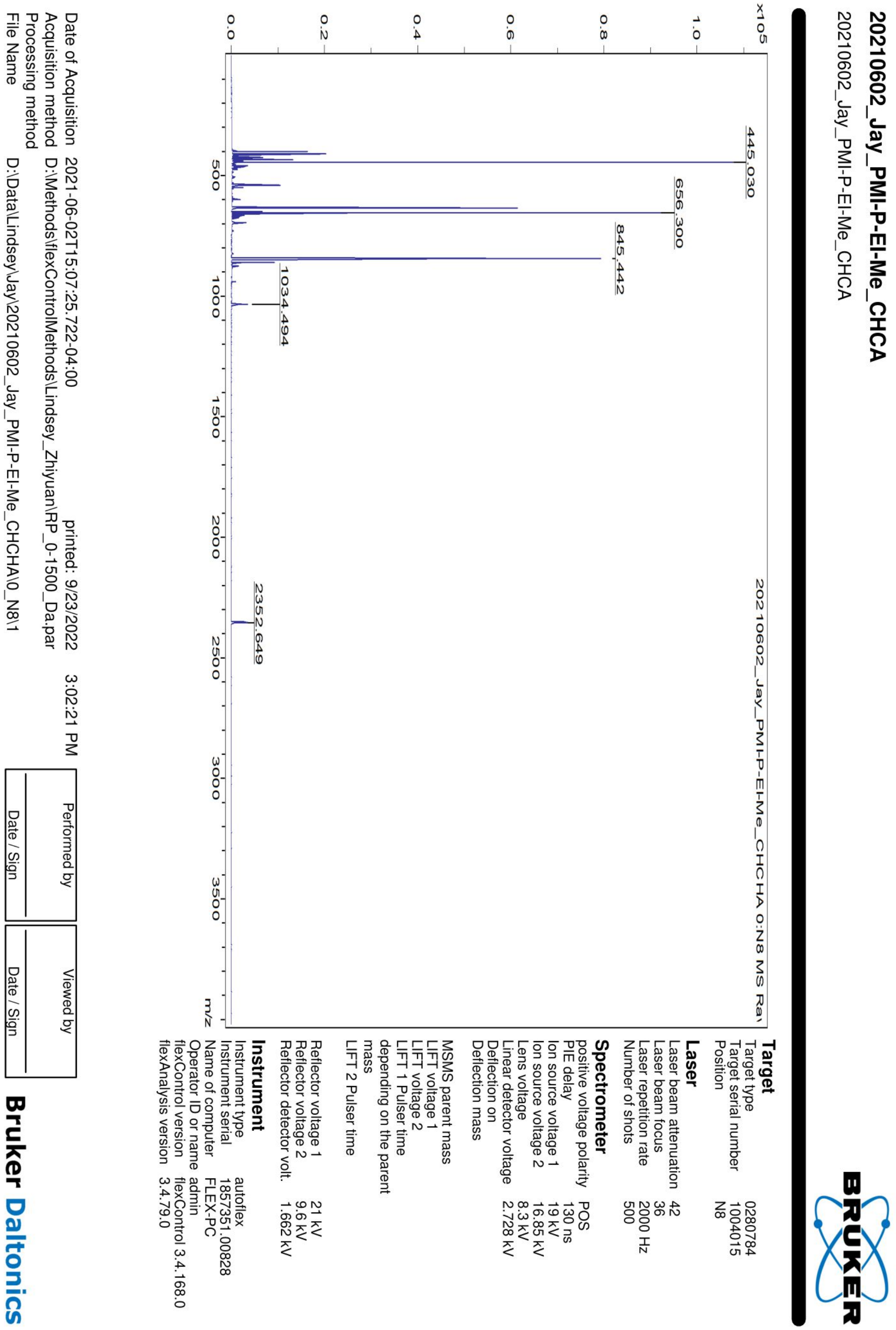

20210602_Jay_PMI-P-EI-Me_CHCA
20210602_Jay_PMI-P-EI-Me_CHCA
Intens. [a.u.]
x10^5
1.0
0.8
0.6
0.4
0.2
0.0
445.030
656.300
845.442
1034.494
2352.649
20210602_Jay_PMI-P-EI-Me_CHCHA 0:N8 MS Ra
500
1000
1500
2000
2500
3000
3500
m/z
Target
Target type 0280784
Target serial number 1004015
Position N8
Laser
Laser beam attenuation 42
Laser beam focus 36
Laser repetition rate 2000 Hz
Number of shots 500
Spectrometer
positive voltage polarity POS
PIE delay 130 ns
Ion source voltage 1 19 kV
Ion source voltage 2 16.85 kV
Lens voltage 8.3 kV
Linear detector voltage 2.728 kV
Deflection on
Deflection mass
MSMS parent mass
LIFT voltage 1
LIFT voltage 2
LIFT 1 Pulser time
depending on the parent mass
LIFT 2 Pulser time
Reflector voltage 1 21 kV
Reflector voltage 2 9.6 kV
Reflector detector volt. 1.662 kV
Instrument
Instrument type autoflex
Instrument serial 1857351.00828
Name of computer FLEX-PC
Operator ID or name admin
flexControl version flexControl 3.4.168.0
flexAnalysis version 3.4.79.0
Date of Acquisition 2021-06-02T15:07:25.722-04:00
Acquisition method D:\Methods\flexControlMethods\Lindsey_Zhiyuan\RP_0-1500_Da.par
Processing method
File Name D:\Data\Lindsey\Jay\20210602_Jay_PMI-P-EI-Me_CHCHA\0_N8\1
printed: 9/23/2022 3:02:21 PM
Performed by
Date / Sign
Viewed by
Date / Sign
BRUKER
Bruker Daltonics

20210602_Jay_PMI-P-EI-Me_CHCA

20210602_Jay_PMI-P-EI-Me_CHCA

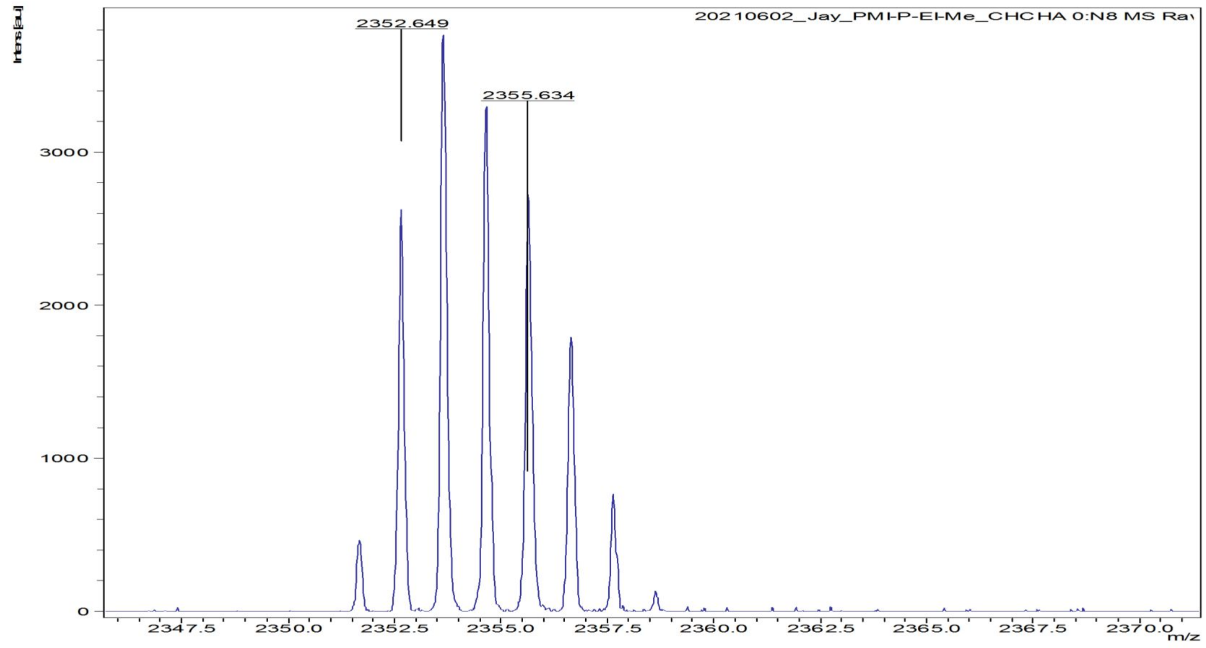

Target

Target type	0280784
Target serial number	1004015
Position	N8

Laser

Laser beam attenuation	42
Laser beam focus	36
Laser repetition rate	2000 Hz
Number of shots	500

Spectrometer

positive voltage polarity	POS
PIE delay	130 ns
Ion source voltage 1	19 kV
Ion source voltage 2	16.85 kV
Lens voltage	8.3 kV
Linear detector voltage	2.728 kV
Deflection on	
Deflection mass	
MSMS parent mass	
LIFT voltage 1	
LIFT voltage 2	
LIFT 1 Pulser time depending on the parent mass	
LIFT 2 Pulser time	
Reflector voltage 1	21 kV
Reflector voltage 2	9.6 kV
Reflector detector volt.	1.662 kV

Instrument

Instrument type	autoflex
Instrument serial	1857351.00828
Name of computer	FLEX-PC
Operator ID or name	admin
flexControl version	flexControl 3.4.168.0
flexAnalysis version	3.4.79.0

Date of Acquisition 2021-06-02T15:07:25.722-04:00 printed: 9/23/2022 3:02:55 PM
Acquisition method D:\Methods\flexControlMethods\Lindsey_Zhiyuan\RP_0-1500_Da.par
Processing method
File Name D:\Data\Lindsey\Jay\20210602_Jay_PMI-P-EI-Me_CHCHA\0_N8\1

Performed by	Viewed by
Date / Sign	Date / Sign

Bruker Daltonics

30: ^{1}H NMR, $CDCl_3$, 600 MHz

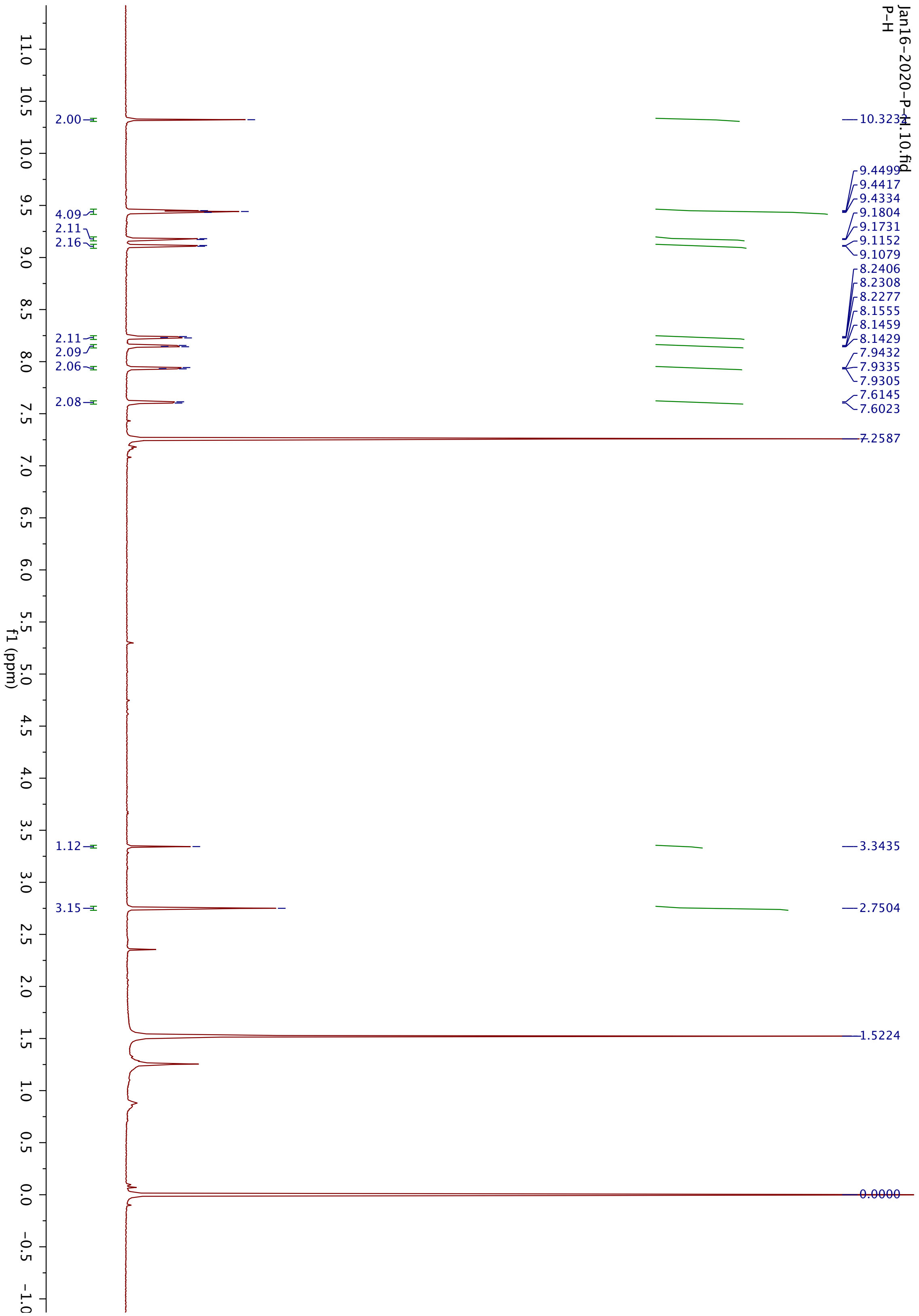

30: MALDI-MS

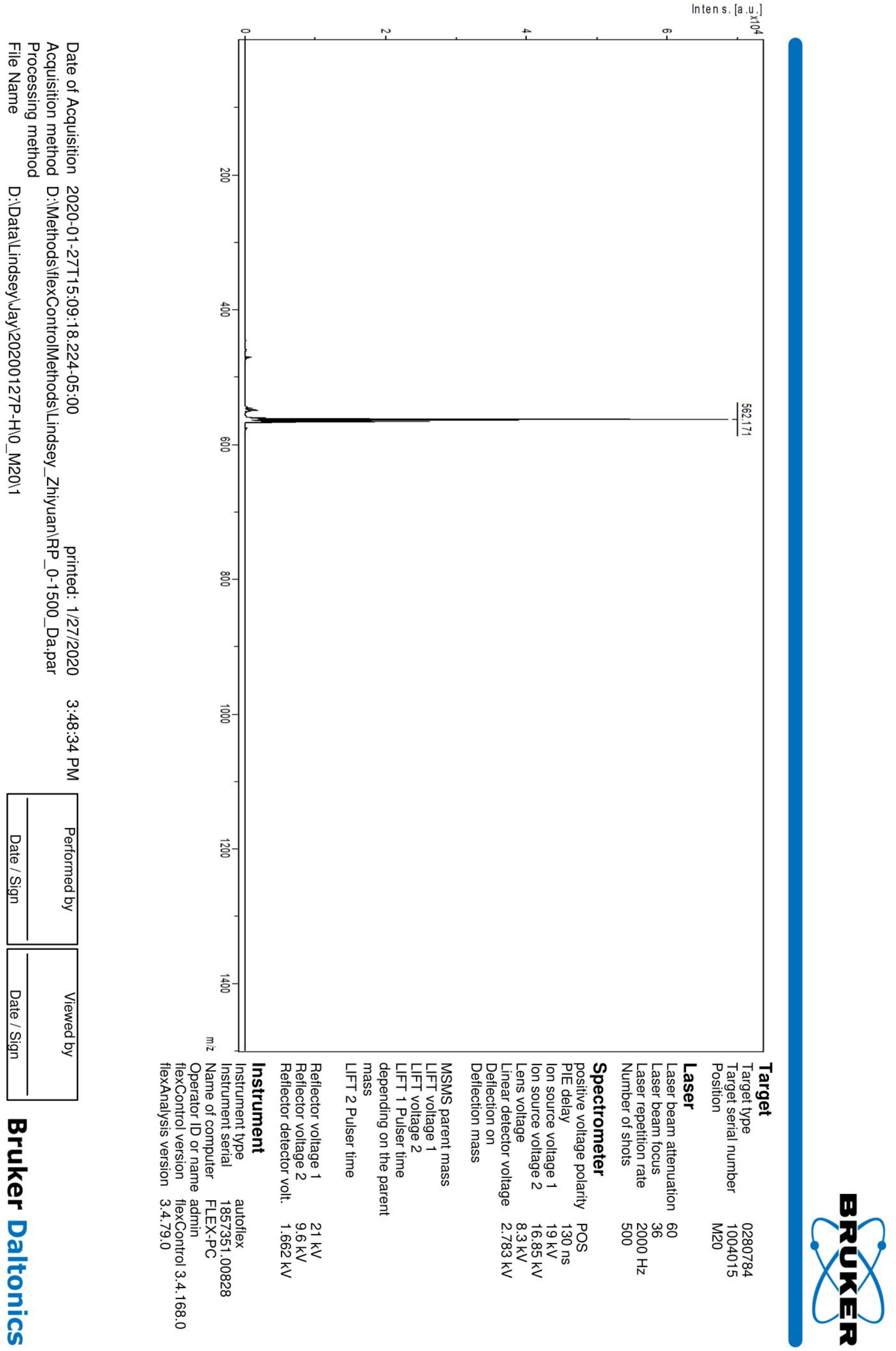
Inten s. [a.u.]
x10^4
562.171
m/z
Date of Acquisition 2020-01-27T15:09:18.224-05:00
Acquisition method D:\Methods\flexControlMethods\Lindsey_Zhiyuan\RP_0-1500_Da.par
Processing method
File Name D:\Data\Lindsey\Jay\20200127P-H\0_M20\1
printed: 1/27/2020 3:48:34 PM
Performed by
Date / Sign
Viewed by
Date / Sign
Bruker Daltonics
Target
Target type 0280784
Target serial number 1004015
Position M20
Laser
Laser beam attenuation 60
Laser beam focus 36
Laser repetition rate 2000 Hz
Number of shots 500
Spectrometer
positive voltage polarity POS
PIE delay 130 ns
Ion source voltage 1 19 kV
Ion source voltage 2 16.85 kV
Lens voltage 8.3 kV
Linear detector voltage 2.783 kV
Deflection on
Deflection mass
MSMS parent mass
LIFT voltage 1
LIFT voltage 2
LIFT 1 Pulser time
depending on the parent mass
LIFT 2 Pulser time
Reflector voltage 1 21 kV
Reflector voltage 2 9.6 kV
Reflector detector volt. 1.662 kV
Instrument
Instrument type autoflex
Instrument serial 1857351.00828
Name of computer FLEX-PC
Operator ID or name admin
flexControl version flexControl 3.4.168.0
flexAnalysis version 3.4.79.0

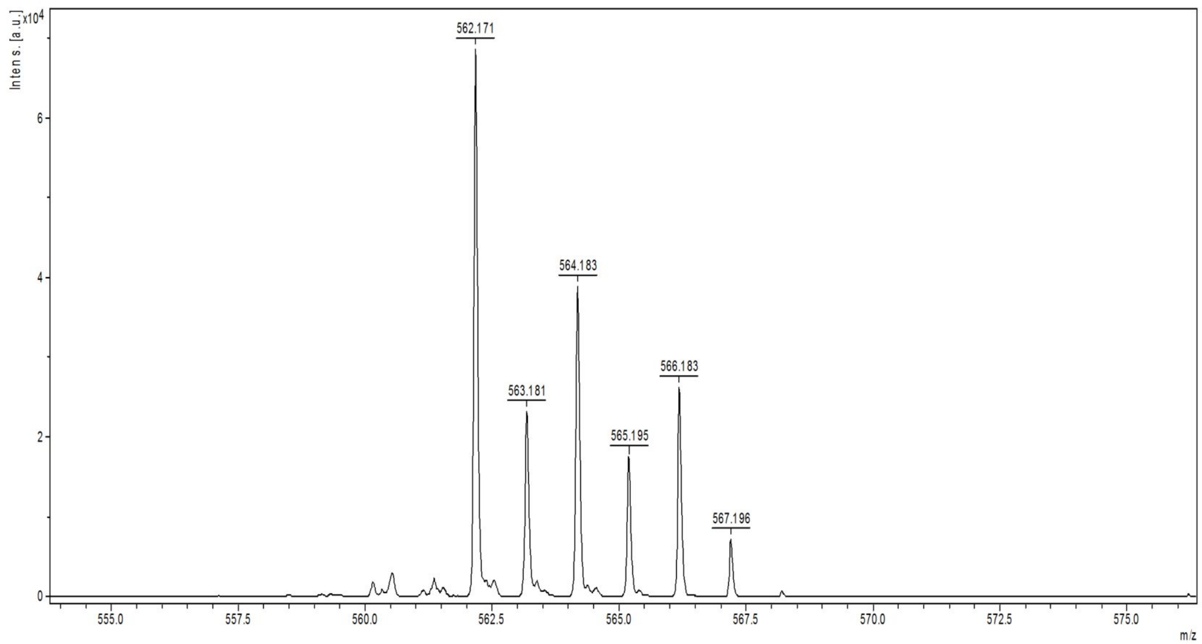

Target

Target type	0280784
Target serial number	1004015
Position	M20

Laser

Laser beam attenuation	60
Laser beam focus	36
Laser repetition rate	2000 Hz
Number of shots	500

Spectrometer

positive voltage polarity	POS
PIE delay	130 ns
Ion source voltage 1	19 kV
Ion source voltage 2	16.85 kV
Lens voltage	8.3 kV
Linear detector voltage	2.783 kV
Deflection on	
Deflection mass	
MSMS parent mass	
LIFT voltage 1	
LIFT voltage 2	
LIFT 1 Pulser time depending on the parent mass	
LIFT 2 Pulser time	
Reflector voltage 1	21 kV
Reflector voltage 2	9.6 kV
Reflector detector volt.	1.662 kV

Instrument

Instrument type	autoflex
Instrument serial	1857351.00828
Name of computer	FLEX-PC
Operator ID or name	admin
flexControl version	flexControl 3.4.168.0
flexAnalysis version	3.4.79.0

Date of Acquisition	2020-01-27T15:09:18.224-05:00 printed: 1/27/2020 3:48:27 PM
Acquisition method	D:\Methods\flexControlMethods\Lindsey_Zhiyuan\RP_0-1500_Da.par
Processing method	
File Name	D:\Data\Lindsey\Jay\20200127P-H\0_M20\1

Performed by	Viewed by
Date / Sign	Date / Sign

Bruker Daltonics

30: Absorption spectrum (toluene)

```
==============================================================================
Spectrum/Peak Report                      Date 9/23/2022 Time 12:01:24  Page   1 of 1
==============================================================================


Method file :         <method not saved>
Information :         Default Method
Data File   :         C:\Chem32\1\DATA\JayRong\Solar_Cell\ABS for paper\P-H_1.SD
                      Created : 1/17/20 15:36:25
```

Compound 30

Overlaid Spectra:

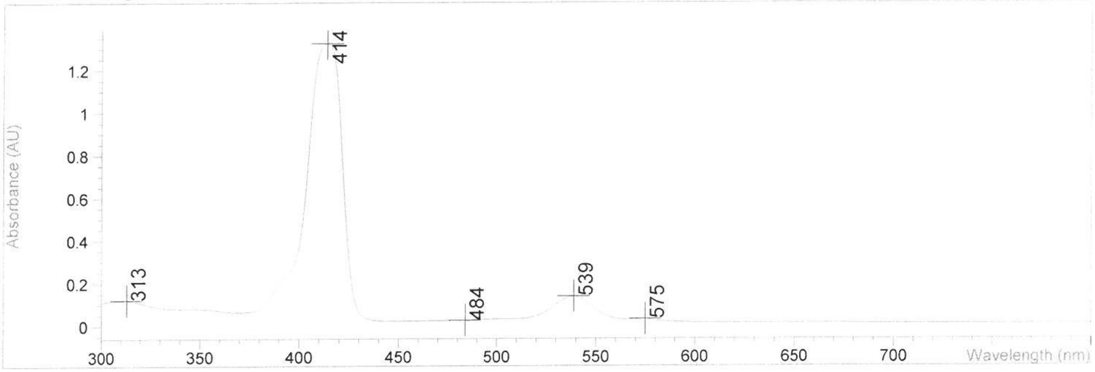

#	Name	Peaks(nm)	Abs(AU)
1		414.0	1.32440
1		539.0	0.13791
1		313.0	0.12170
1		575.0	3.1120E-2
1		484.0	2.5795E-2

Report generated by : jon   Signature: ....

```
------------------------------------------------------------------------------
                    *** End Spectrum/Peak Report ***
------------------------------------------------------------------------------
```

31: ^{1}H NMR, $CDCl_3$, 600 MHz

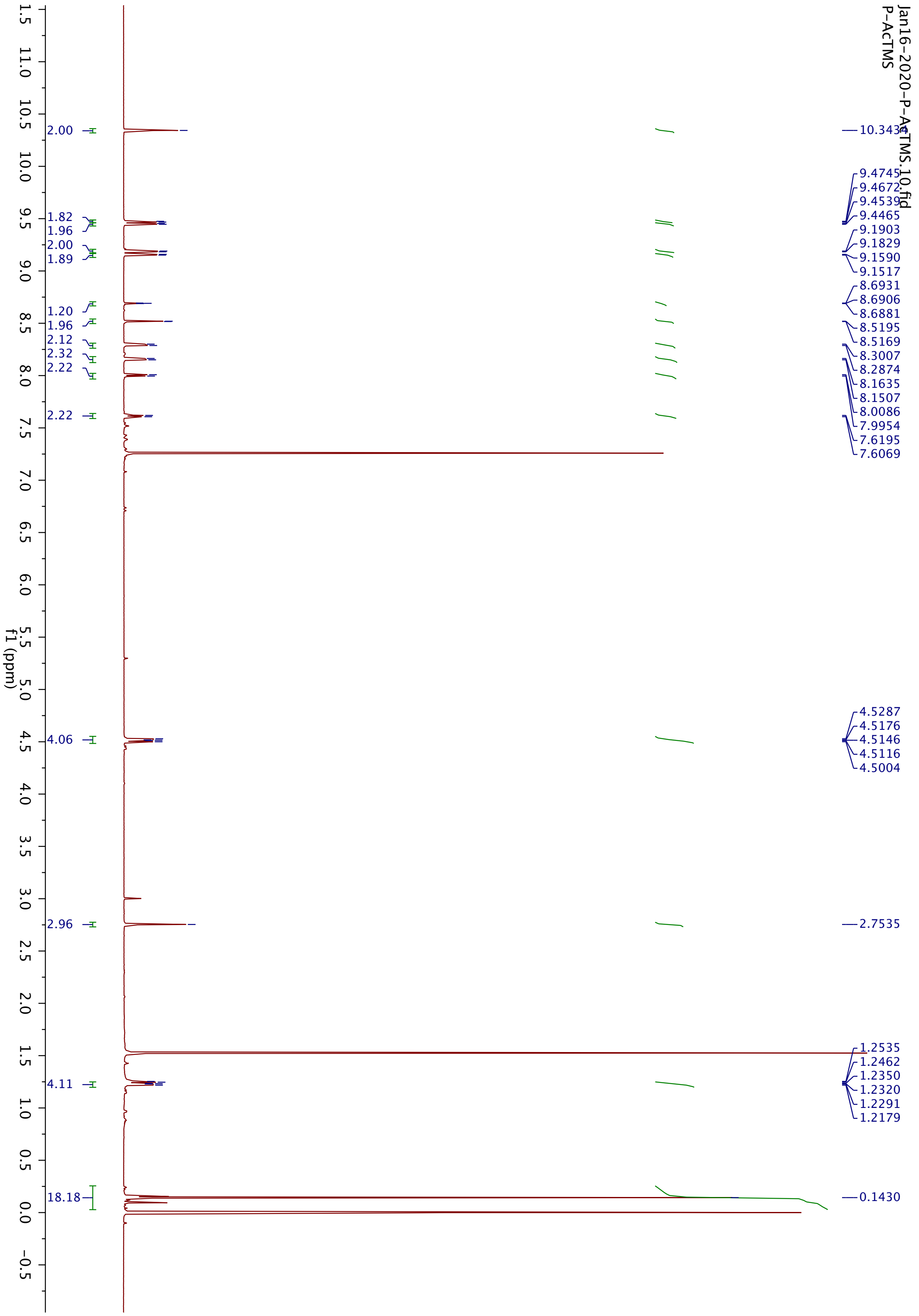

31: MALDI-MS

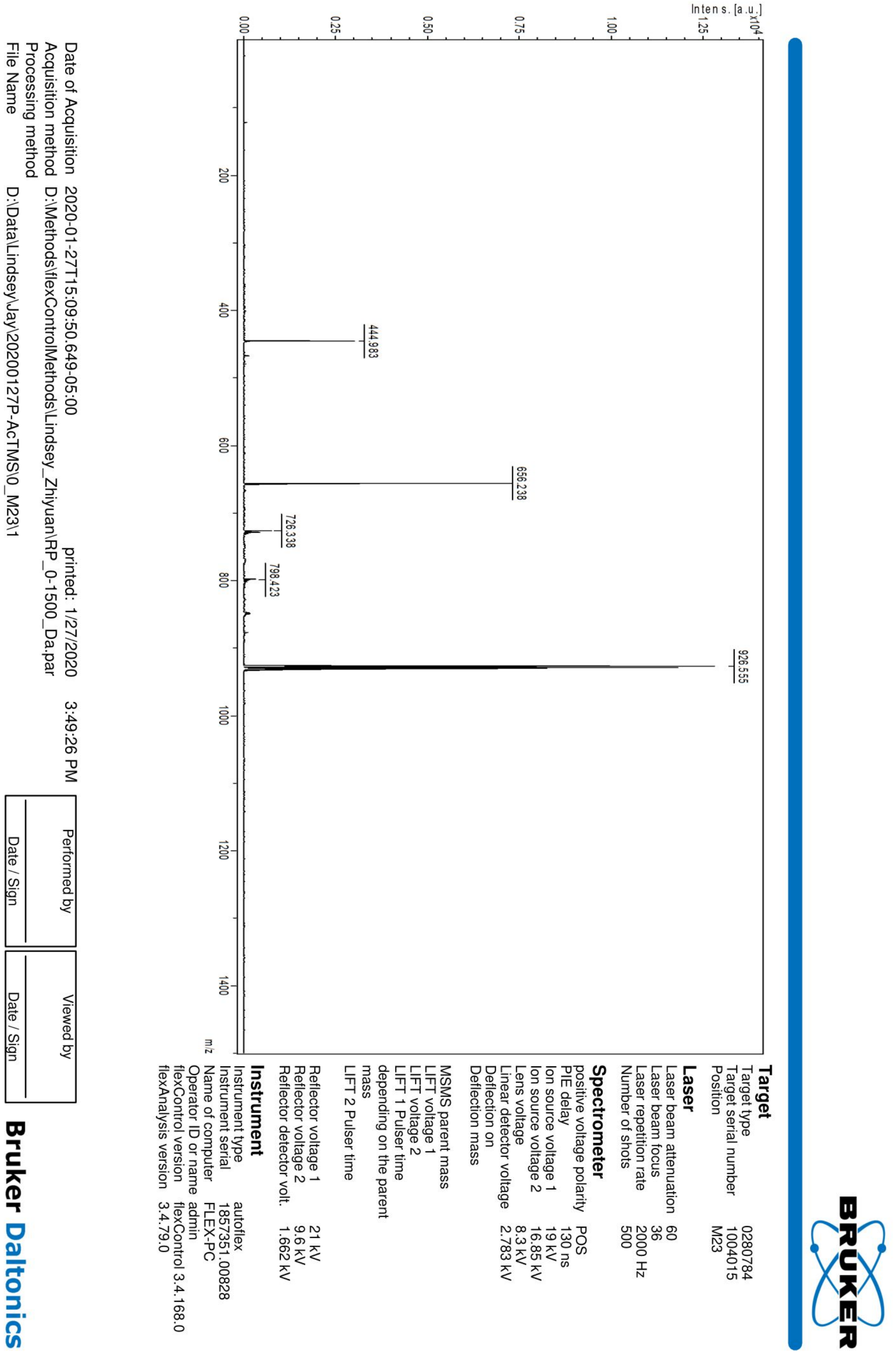

Intens. [a.u.]
x10^4
1.25
1.00
0.75
0.50
0.25
0.00
200
400
600
800
1000
1200
1400
m/z
444.983
656.238
726.338
798.423
926.55
Target
Target type 0280784
Target serial number 1004015
Position M23
Laser
Laser beam attenuation 60
Laser beam focus 36
Laser repetition rate 2000 Hz
Number of shots 500
Spectrometer
positive voltage polarity POS
PIE delay 130 ns
Ion source voltage 1 19 kV
Ion source voltage 2 16.85 kV
Lens voltage 8.3 kV
Linear detector voltage 2.783 kV
Deflection on
Deflection mass
MSMS parent mass
LIFT voltage 1
LIFT voltage 2
LIFT 1 Pulser time
depending on the parent mass
LIFT 2 Pulser time
Reflector voltage 1 21 kV
Reflector voltage 2 9.6 kV
Reflector detector volt. 1.662 kV
Instrument
Instrument type autoflex
Instrument serial 1857351.00828
Name of computer FLEX-PC
Operator ID or name admin
flexControl version flexControl 3.4.168.0
flexAnalysis version 3.4.79.0
Date of Acquisition 2020-01-27T15:09:50.649-05:00 printed: 1/27/2020 3:49:26 PM
Acquisition method D:\Methods\flexControlMethods\Lindsey_Zhiyuan\RP_0-1500_Da.par
Processing method
File Name D:\Data\Lindsey\Jay\20200127P-AcTMS\0_M23\1
Performed by
Date / Sign
Viewed by
Date / Sign
Bruker Daltonics

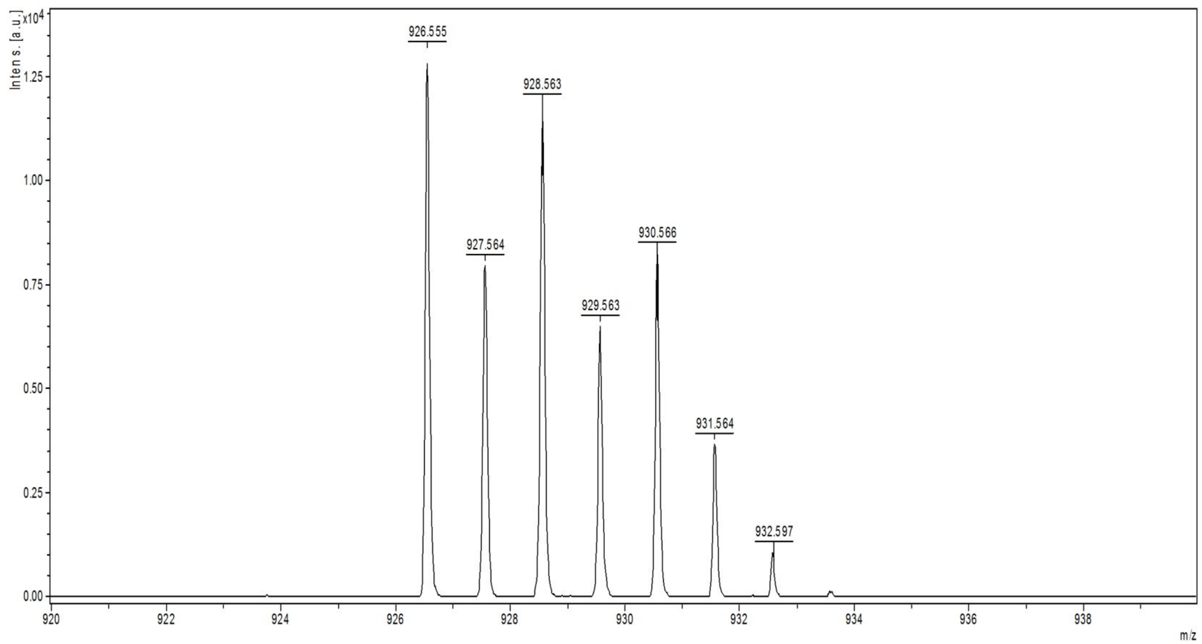

Target

Target type	0280784
Target serial number	1004015
Position	M23

Laser

Laser beam attenuation	60
Laser beam focus	36
Laser repetition rate	2000 Hz
Number of shots	500

Spectrometer

positive voltage polarity	POS
PIE delay	130 ns
Ion source voltage 1	19 kV
Ion source voltage 2	16.85 kV
Lens voltage	8.3 kV
Linear detector voltage	2.783 kV
Deflection on	
Deflection mass	
MSMS parent mass	
LIFT voltage 1	
LIFT voltage 2	
LIFT 1 Pulser time depending on the parent mass	
LIFT 2 Pulser time	
Reflector voltage 1	21 kV
Reflector voltage 2	9.6 kV
Reflector detector volt.	1.662 kV

Instrument

Instrument type	autoflex
Instrument serial	1857351.00828
Name of computer	FLEX-PC
Operator ID or name	admin
flexControl version	flexControl 3.4.168.0
flexAnalysis version	3.4.79.0

Date of Acquisition	2020-01-27T15:09:50.649-05:00 printed: 1/27/2020 3:49:19 PM
Acquisition method	D:\Methods\flexControlMethods\Lindsey_Zhiyuan\RP_0-1500_Da.par
Processing method	
File Name	D:\Data\Lindsey\Jay\20200127P-AcTMS\0_M23\1

Performed by	Viewed by
Date / Sign	Date / Sign

Bruker Daltonics

31: Absorption spectrum (toluene)

Spectrum/Peak Report Date 9/23/2022 Time 12:01:24 Page 1 of 1

Method file : <method not saved>
Information : Default Method
Data File : C:\Chem32\1\DATA\JayRong\Solar_Cell\ABS for paper\P-H_1.SD
Created : 1/17/20 15:36:25

Compound 30

Overlaid Spectra:

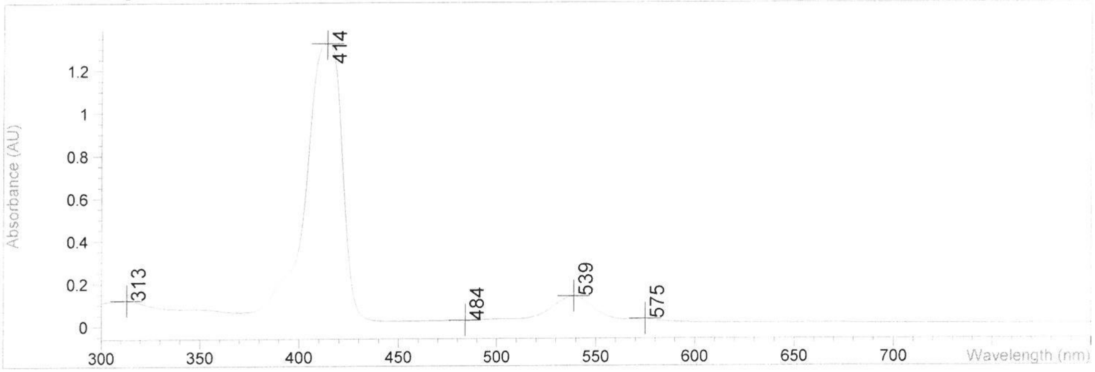

#	Name	Peaks (nm)	Abs (AU)
1		414.0	1.32440
1		539.0	0.13791
1		313.0	0.12170
1		575.0	3.1120E-2
1		484.0	2.5795E-2

Report generated by : jon Signature: ....

*** End Spectrum/Peak Report ***

32: ^{1}H NMR, $CDCl_3$, 600 MHz

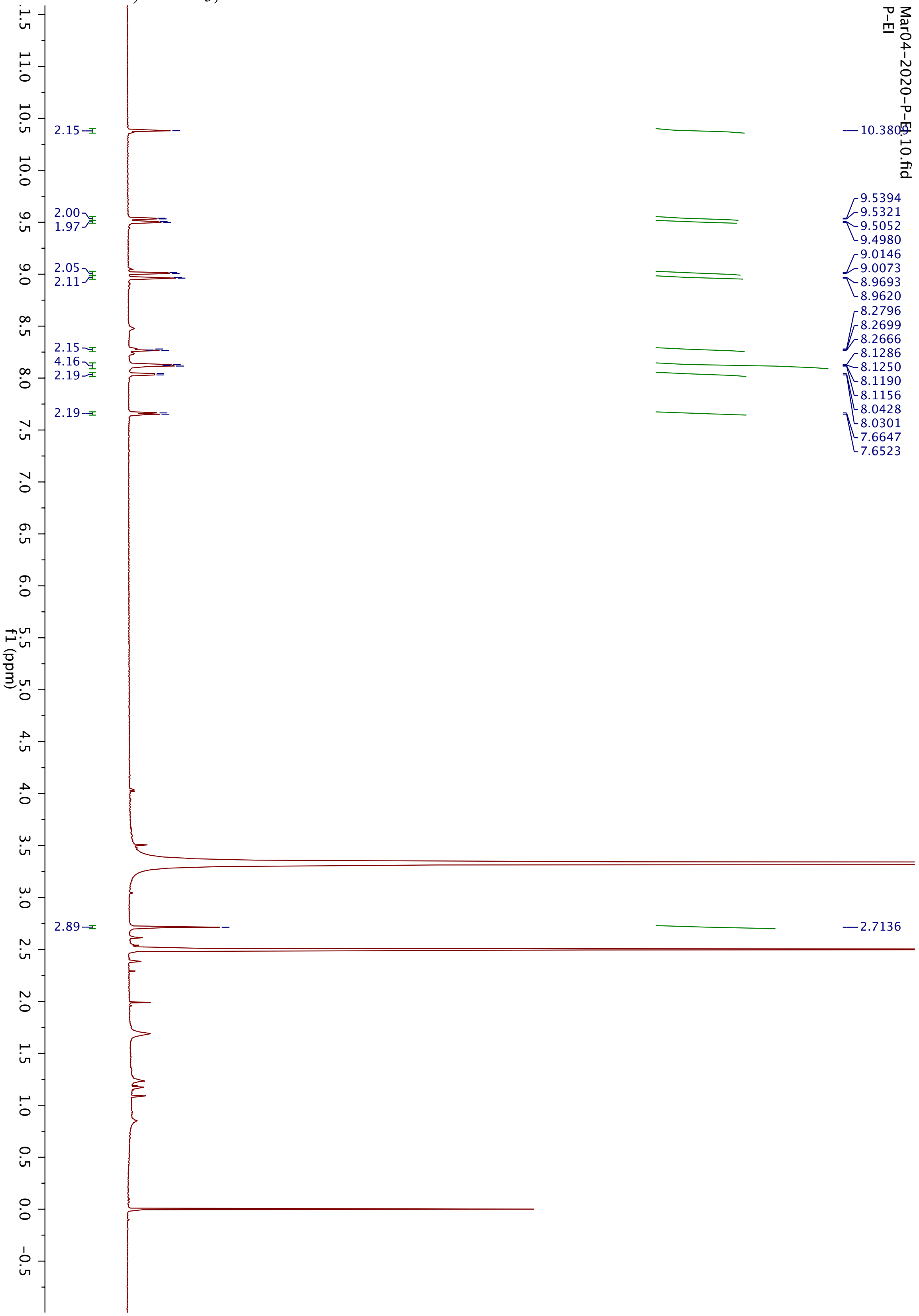

32: Absorption spectrum (DMSO)

Spectrum/Peak Report   Date 9/24/2022 Time 15:27:30   Page 1 of 1

Method file : <method not saved>
Information : Default Method
Data File : C:\Chem32\1\DATA\Masa\J37 PORPHYRIN DMSO.SD   Created : 9/23/22 19:47:19

Overlaid Spectra:

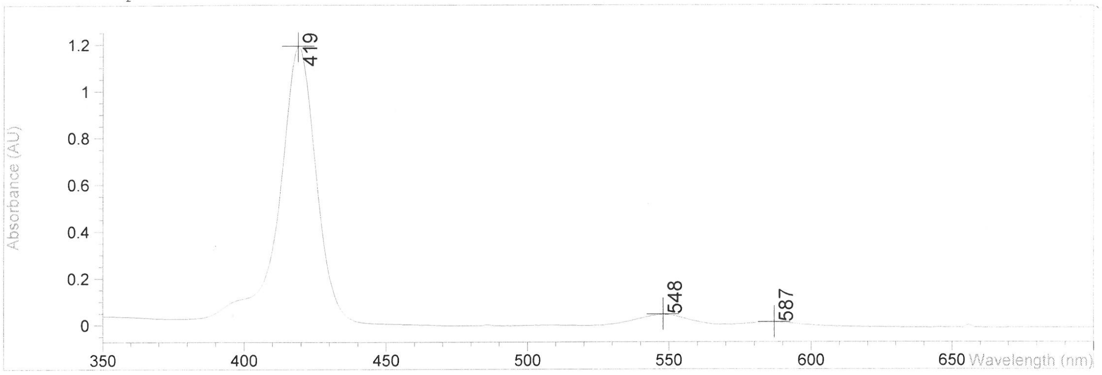

#	Name	Peaks(nm)	Abs(AU)
1		419.0	1.19270
1		548.0	4.6544E-2
1		587.0	1.3256E-2

Report generated by : jon   Signature: ....

*** End Spectrum/Peak Report ***

32: ESI-MS

NC STATE UNIVERSITY
Molecular Education, Technology and
Research Innovation Center
Raleigh, NC 27695
https://research.ncsu.edu/metric/

ZnP-EI Experimental and Theoretical Isotopic Distribution for $C_{43}H_{26}N_4O_4Zn$ [M+H]$^+$

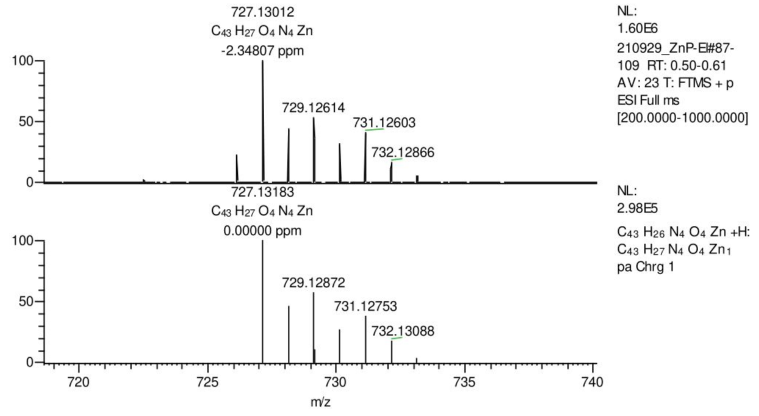

ZnP-EI Experimental and Theoretical Isotopic Distribution for $C_{43}H_{26}N_4O_4Zn$ [M-H]$^-$

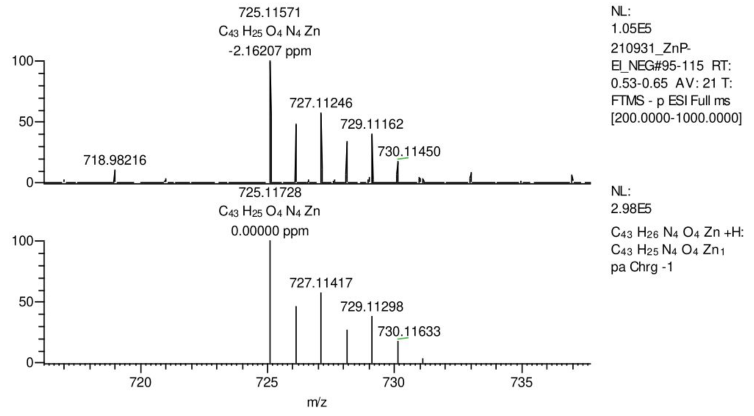

Page **1** of **1**
